# Improved predictive models for acute kidney injury with IDEA: Intraoperative Data Embedded Analytics

**DOI:** 10.1371/journal.pone.0214904

**Published:** 2019-04-04

**Authors:** Lasith Adhikari, Tezcan Ozrazgat-Baslanti, Matthew Ruppert, R. W. M. A. Madushani, Srajan Paliwal, Haleh Hashemighouchani, Feng Zheng, Ming Tao, Juliano M. Lopes, Xiaolin Li, Parisa Rashidi, Azra Bihorac

**Affiliations:** 1 Division of Nephrology, Hypertension and Renal Transplantation, Department of Medicine, University of Florida, Gainesville, FL, United States of America; 2 Precision and Intelligent Systems in Medicine (PrismaP), University of Florida, Gainesville, FL, United States of America; 3 Department of Electrical and Computer Engineering, University of Florida, Gainesville, FL, United States of America; 4 Biomedical Engineering Department, University of Florida, Gainesville, FL, United States of America; Universita degli Studi di Pisa, ITALY

## Abstract

**Background:**

Acute kidney injury (AKI) is a common complication after surgery that is associated with increased morbidity and mortality. The majority of existing perioperative AKI risk prediction models are limited in their generalizability and do not fully utilize intraoperative physiological time-series data. Thus, there is a need for intelligent, accurate, and robust systems to leverage new information as it becomes available to predict the risk of developing postoperative AKI.

**Methods:**

A retrospective single-center cohort of 2,911 adults who underwent surgery at the University of Florida Health between 2000 and 2010 was utilized for this study. Machine learning and statistical analysis techniques were used to develop perioperative models to predict the risk of developing AKI during the first three days after surgery, first seven days after surgery, and overall (after surgery during the index hospitalization). The improvement in risk prediction was examined by incorporating intraoperative physiological time-series variables. Our proposed model enriched a preoperative model that produced a probabilistic AKI risk score by integrating intraoperative statistical features through a machine learning stacking approach inside a random forest classifier. Model performance was evaluated using the area under the receiver operating characteristic curve (AUC), accuracy, and Net Reclassification Improvement (NRI).

**Results:**

The predictive performance of the proposed model is better than the preoperative data only model. The proposed model had an AUC of 0.86 (accuracy of 0.78) for the seven-day AKI outcome, while the preoperative model had an AUC of 0.84 (accuracy of 0.76). Furthermore, by integrating intraoperative features, the algorithm was able to reclassify 40% of the false negative patients from the preoperative model. The NRI for each outcome was AKI at three days (8%), seven days (7%), and overall (4%).

**Conclusions:**

Postoperative AKI prediction was improved with high sensitivity and specificity through a machine learning approach that dynamically incorporated intraoperative data.

## Introduction

Acute kidney injury (AKI) is one of the most common, yet underdiagnosed, postoperative complications with lasting consequences [1, 2]. It is associated with an increase in mortality, short- and long-term morbidity, chronic kidney disease, and cardiovascular disease [3–7]. An episode of postoperative AKI imposes an average hospital cost increase of $9000, even after adjusting for all other complications [8, 9]. The implementation of existing clinical guidelines for prevention and treatment of AKI is often hindered by the inability to accurately and timely assess the risk for AKI while accounting for the dynamic nature of the pathophysiological events during surgery.

With the advancement of digitalization in clinical medicine and the widespread availability of electronic health records (EHR), a number of predictive models have been developed to estimate the risk for AKI in different clinical settings, including after surgery [10, 11]. A majority of the existing AKI risk models are limited to preoperative factors [12], applicable only to a specific surgery type [13, 14]. Some of the available online prognostic calculators [15] are designed for intensive care unit (ICU) patients only, and do not take any surgical features into account. While preoperative models for AKI mainly rely on patients’ pre-existing health conditions and general risks associated with the type of surgical procedure, there is a wealth of intraoperative data reflecting the acute physiological responses to the stresses of surgery that is being ignored. Although recent studies have demonstrated an association between AKI and low intraoperative hemoglobin and hypotension [7, 16, 17], there is still a lack of comprehensive postoperative AKI prediction models that dynamically integrate physiologic intraoperative data with preoperative information.

Thus, there is a need for intelligent, accurate, and robust systems that are able to leverage temporal information related to patients’ physiological changes during surgery. We have recently developed and validated a machine learning algorithm, *MySurgeryRisk*, which predicts preoperative risk for major postoperative complications, including AKI, using EHR data [18]. The aim of this study was to develop and validate a dynamic machine-learning algorithm that readjusts the preoperative risk for AKI, using physiological time series and other data collected during surgery, to provide a personalized risk panel for AKI with both preoperative and immediate postoperative risk assessments.

## Materials and methods

This study was approved by the University of Florida Institutional Review Board and Privacy Office as an exempt study with a waiver of informed consent. Transparent Reporting of a multivariable prediction model for Individual Prognosis Or Diagnosis (TRIPOD) recommendations were followed under the Type 2a analysis category (random split sample development and validation) (S1 Table) [19].

### Data source

The University of Florida Integrated Data Repository was used as an honest broker to assemble a single center longitudinal perioperative cohort for all patients admitted to the University of Florida Health for longer than 24 hours following any type of operative procedure between January 1, 2000 and November 30, 2010 by integrating electronic health records with other clinical, administrative, and public databases as previously described [12]. The resulting dataset included detailed information on patient demographics, diagnoses, procedures, outcomes, comprehensive hospital charges, hospital characteristics, insurance status, laboratory, pharmacy, and blood bank data as well as detailed intraoperative physiologic and monitoring data for the cohort.

### Participants

We identified patients 18 years of age or older that were admitted to the hospital for longer than 24 hours following any type of inpatient operative procedure. If patients underwent multiple surgeries, only the first surgery was used in our analysis. Patients with end stage renal disease prior to admission (n = 1,935) and patients with missing serum creatinine values during hospitalization (n = 6,636) were excluded from our analysis. From the remaining cohort there were 2,911 patients who had complete intraoperative data for all vital signs, laboratory values, and medications.

### Outcomes

The main outcome was the development of postoperative AKI within the first seven days after surgery. The secondary analysis modeled a) the risk for the development of AKI within the first three days after surgery, and b) the risk of developing postoperative AKI at any point during the hospitalization for the index surgery. AKI was defined using the consensus Kidney Disease: Improving Global Outcomes (KDIGO) criteria as at least a 50% or 0.3 mg/dl increase in serum creatinine relative to the reference creatinine [20]. Reference creatinine was determined based on the availability of measured creatinine prior to admission. The minimum serum creatinine value was used if results were available within seven days of the index hospitalization. If not available, the median serum creatinine value obtained within 8–365 days prior to admission was used. For patients without a prior creatinine value within the year prior to admission and no history of chronic kidney disease, an estimated reference serum creatinine was used [21–23]. The estimated reference serum creatinine was calculated by solving the abbreviated “Modification of Diet in Renal Disease” equation for creatinine, assuming a glomerular filtration rate of 75 ml/minute/1.73 m^2^. After the first seven days of the index hospitalization, the minimum serum creatinine from the preceding seven days was used as the reference creatinine [12, 24]. Patients with chronic kidney disease and end stage renal disease prior to admission were identified using the validated combination of The International Classification of Diseases, Ninth Revision, Clinical Modification (ICD-9-CM) codes [25, 26]. Exact dates were used to calculate the duration of mechanical ventilation and intensive care unit stay. A set of previously described criteria was used to annotate the remaining clinical outcomes [8].

### Predictor features

The preoperative risk assessment used demographic, socio-economic, administrative, clinical, pharmacy, and laboratory data available prior to surgery to derive 285 preoperative predictor features from 69 preoperative variables (S2 Table). Preoperative comorbidities were derived using up to 50 ICD-9-CM codes as binary variables and the Charlson Comorbidity Index [7, 18, 27, 28]. A reference estimated glomerular filtration rate (eGFR) was calculated for all patients using the reference serum creatinine, sex, race, and age [29]. The immediate postoperative risk reassessment used the following intraoperative variables: five physiologic time series (mean arterial blood pressure (MAP), systolic blood pressure, diastolic blood pressure, minimum alveolar concentration (MAC) of inhaled anesthetics, and heart rate (HR)), 21 repeated laboratory measures, and other discrete variables (intraoperative medications, duration of the operation, anesthesia type, etc.) (S2 Table).

### Sample size

Two thousand nine hundred eleven (2,911) patients were included in the cohort. The data was randomly split into 70% for training and 30% for validation. The algorithm was trained on the development cohort while results were reported from the validation cohort. By using 30% of the cohort for validation (n = 873), the overall sample size allows for a maximum width of the 95% confidence interval for the area under the receiver operating characteristic curve (AUC) of 0.08, when prevalence of AKI is between 30% and 40%.

### Predictive analytics workflow

**Fig 1:**
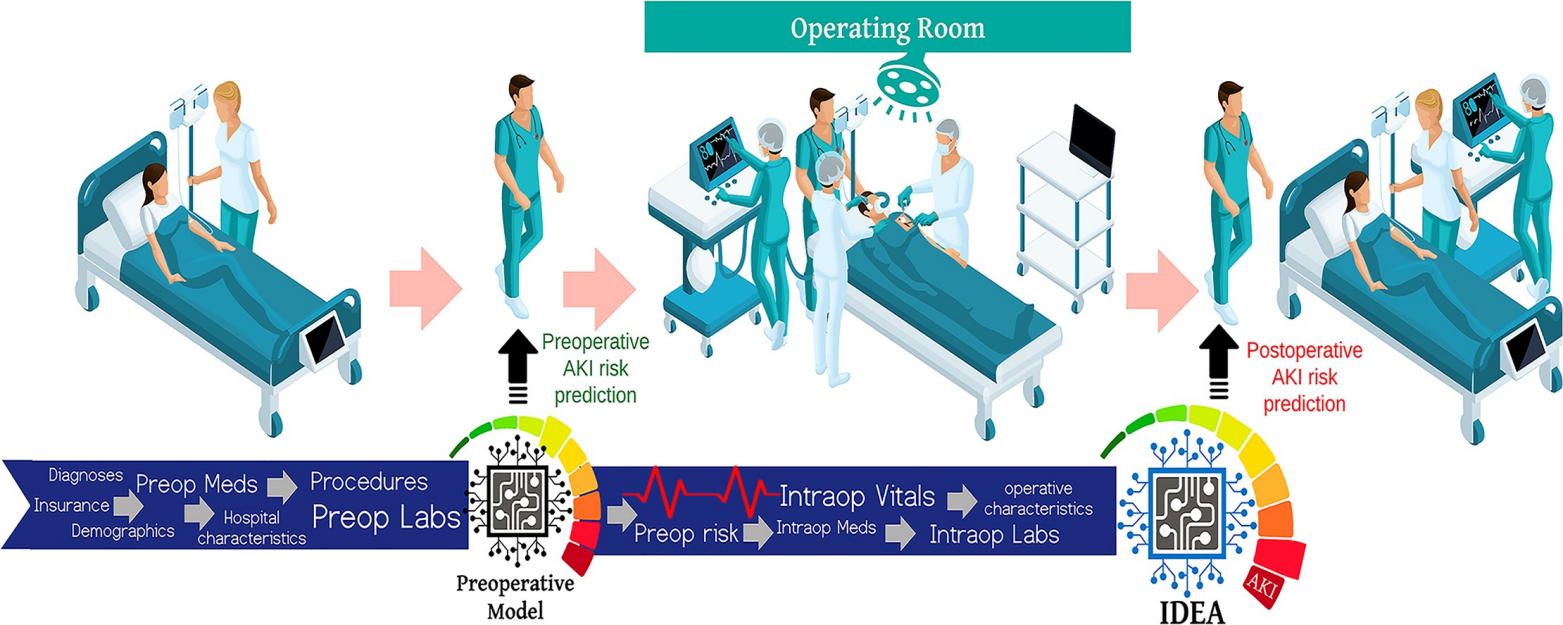
Clinical workflow of the Intraoperative Data Embedded Analytics (*IDEA*) algorithm for postoperative acute kidney injury prediction. Phase I, all available health care data from the electronic health record and other public datasets is fed into the preoperative model. The preoperative model calculates a risk score for postoperative acute kidney injury (AKI) and shares the risk score with the clinical team before the surgery. Phase II, the preoperative data is combined with intraoperative data and fed into the *IDEA* algorithm which calculates a risk score for postoperative acute kidney injury and shares the risk score with the clinical team after surgery.

**Fig 2:**
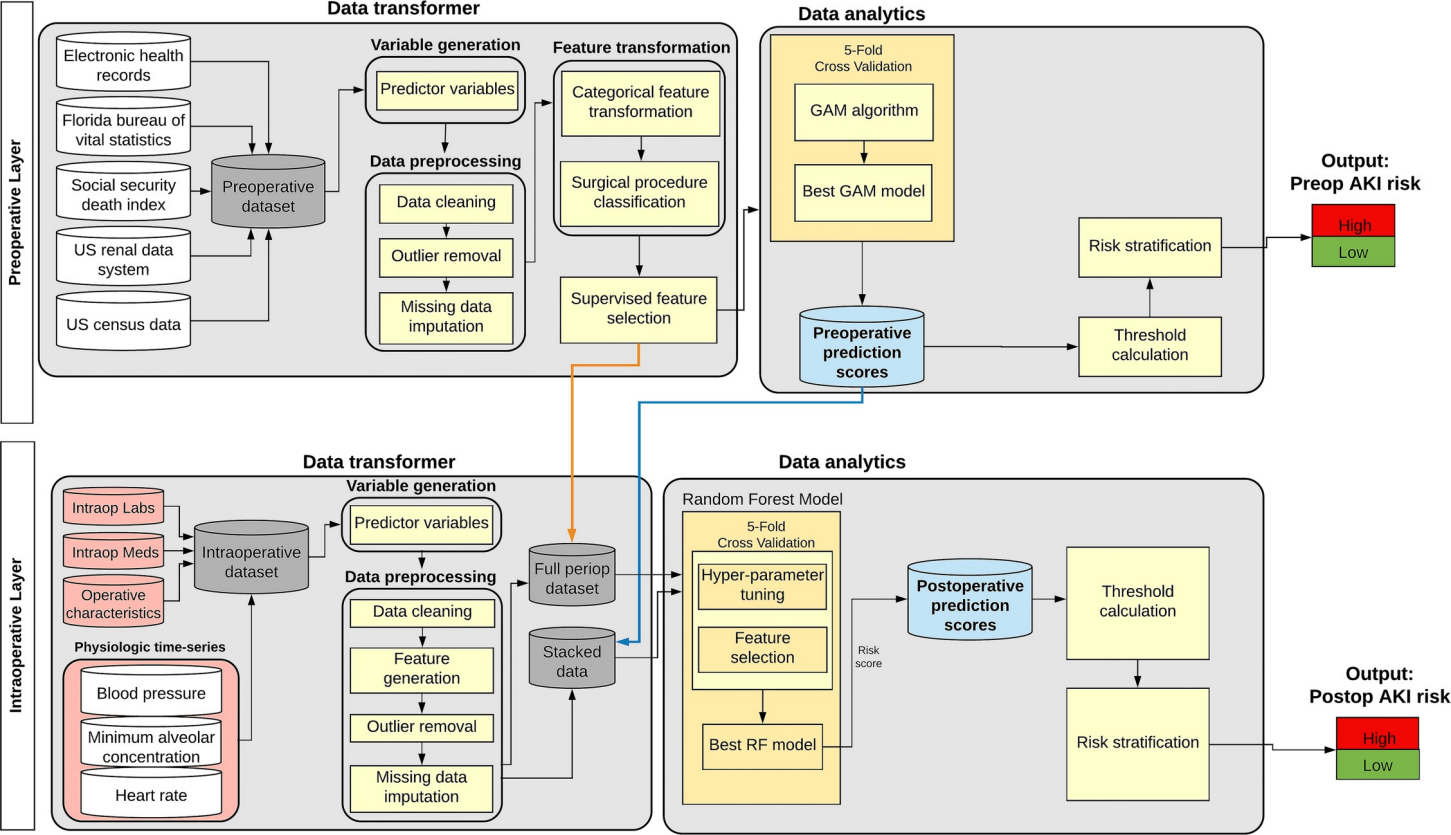
Conceptual diagram of the Intraoperative Data Embedded Analytics (*IDEA*) AKI prediction model. This diagram shows the aggregation of the data transformer, data engineering, and data analytics modules in the preoperative and intraoperative layers. The two layers can be integrated by either (1) stacking the preoperative prediction scores with the cleaned and feature engineered intraoperative data (blue arrow) or (2) obtaining the full perioperative dataset by merging all the clean features from both layers (orange arrow).

The proposed *IDEA* (Intraoperative Data Embedded Analytics) algorithm is conceptualized as a dynamic model that readjusts the preoperative risk for AKI using physiological time series and other data collected *during surgery*. The resulting adjusted postoperative risk is assessed immediately at the end of surgery. This flow simulates the clinical task faced by physicians involved in perioperative care where patients’ preoperative information is subsequently enriched by the influx of new data from the operating room. The final output produces *IDEA*, a personalized risk panel for AKI after surgery (Fig 1) with both preoperative and immediate postoperative risk assessments. The *IDEA* algorithm consists of two main layers, preoperative and intraoperative, (Fig 2) each containing two cores, *data transformer* and *data analytics*.

In the *data transformer core*, the algorithm transforms data from its native format into new complex variables that are optimized for use in predictive models (S2 Table). Data preprocessing was done using a set of automated rules to remove errors and outliers. Time series variables in the intraoperative layer were truncated to match the corresponding surgery start and stop times for each patient. Extreme values in the physiological time series data, defined as values outside the allowable range using expert opinion and medical knowledge [30], were replaced by the average of their five nearest neighbors. Outlier detection and removal was performed on laboratory results by replacing the top and bottom 1% of data using random uniform values generated from the 95%-99.5% and 0.5%-5% percentiles, respectively. Missing nominal variables were replaced with a distinct ‘‘missing” category, whereas missing continuous variables were replaced by the median value for a given variable [18, 31]. Categorical and nominal variables, with more than two levels, were further optimized by calculating conditional probabilities for a particular variable value (such as each surgeon’s ID number or each zip code in the dataset) to be associated with the occurrence of the complication using a separate dataset. The probabilities were calculated as the log of the ratio of the prevalence of a particular variable value among cases with one or more complications to cases without complications [28]. Surgical procedure codes were optimized using a forest of trees approach to reduce the 4-digit primary procedure ICD-9-CM codes that correspond to the anatomical location of surgery. Each node represents a group of procedures, with roots representing the most general groups of procedures and leaf nodes representing the specific procedures [28]. This grouping method reduces the number of discrete procedure codes from 318 to 174 and improves the analysis of low frequency procedures (S1 Methods). Statistical features were derived from the physiological time series data, which included minimum, maximum, mean, short- and long-term variability [32], time spent between different value ranges (determined by the number of standard deviations away from the mean value), and the percentage of time spent in each of the previously mentioned value ranges. Short- and long-term variability were computed using the base and residual signals [32]. The base signal represents the smoothed out version of the time series computed using a convolution filter, while the residual signal represents the difference between the original signal and base signal. The long-term variability is the standard deviation of the base signal and the short-term variability is the standard deviation of the residual signal. For repeated laboratory measurements, the percentage of abnormal values (the percentage of values outside the normal ranges from the Logical Observation Identifiers Names and Codes (LOINC) data table [https://loinc.org/downloads/]), value counts (number of laboratory results obtained during the surgery), and variances were derived (S1 Methods).

In the *data analytics core*, the *IDEA* algorithm was trained to calculate patient-level preoperative and immediate postoperative risk probabilities for AKI. In the first stage, the algorithm was trained to calculate preoperative risk probability for AKI using preoperative data only. Subsequently, the intraoperative data available at the end of surgery was used as an additional input for the algorithm to recalculate the postoperative risk probability for AKI. The preoperative risk probability was calculated using a generalized additive model (GAM) with logistic link function [18, 28, 31]. All models were adjusted for non-linearity of covariates using nonlinear risk functions estimated with thin plate regression splines [18, 28, 33]. The best GAM model was picked using a five-fold cross validation technique on 70% of the randomly selected training data cohort and the preoperative prediction scores were generated as the output.

Intraoperative Data Embedded Analytics (*IDEA*), employing a random forest classifier, was used to enrich the preoperative risk model with intraoperative data. Two approaches were compared, one where risk probability output from the GAM model was combined with the intraoperative data and used as input for the random forest (*stacked model*), and the other where all preoperative data was embedded with the intraoperative data as input for the random forest (*full model*). All intraoperative statistical features, along with the preoperative prediction scores, underwent a univariate analysis and only statistically significant (based on the F-test statistic) features were considered for the random forest model [34]. The feature selection and other hyper parameters in scikit-learn [35] random forest classifier (i.e., number of trees, maximum features for the best split, minimum number of samples required to be at a leaf node) were tuned simultaneously using a grid search technique with five-fold cross validation on the same 70% training data cohort (S1 Fig).

### Model validation

All of the models were validated using the 30% validation cohort of 873 patients (S1 Fig). The results were reported using 1000 nonparametric bootstrap replicates generated from the R boot package [36]. Using the prediction results obtained from the 1000 bootstrap cohorts, nonparametric confidence intervals for each of the performance metrics were calculated.

### Model performance

Each model’s discrimination was assessed using the area under receiver operating characteristic curve (AUC) and model accuracy by determining the fraction of correct classifications for each model. Stratification into high- and low-risk groups was done by calculating the optimal cut-off point based on the maximum Youden Index [37] computed during the training process for each outcome. Using the optimal thresholds for risk probabilities, a classification table was built from which sensitivity, specificity, and positive and negative predictive values were calculated for each model. Absolute risk was calculated as the percentage of cases for which acute kidney injury (AKI) occurred in low- and high-risk groups, respectively. Relative risk was calculated as the ratio of the absolute risk of AKI between high- and low-risk groups. The absolute risk was calculated for high- and low-risk groups for all three models and were compared using the R package “DTComPair” adjusting for multiple comparisons using the Bonferroni method. The Net Reclassification Improvement (NRI) index [38] was used to quantify how well the postoperative model reclassifies AKI patients compared to the preoperative model. Model calibration was tested using the Hosmer-Lemeshow statistic. Bootstrap sampling and nonparametric methods were used to obtain 95% confidence intervals for all performance measures. All analyses were performed using Python 2.7 [39], SciPy 1.0.0 [40], R 3.4 [41], and SAS 9.4 (Cary, NC) software.

## Results

### Participant baseline characteristics and outcomes

Among 2,911 patients who underwent inpatient surgery requiring a hospital admission of at least 24 hours in a quaternary-care academic center, 1,339 (46%) developed postoperative AKI prior to discharge. Of the patients that developed postoperative AKI, 1,163 (87%) had AKI onset within seven days of surgery. Only 176 (13%) of the AKI patients had late AKI onset (more than seven days after surgery), while 75% of the AKI patients had AKI onset within the first 72 hours after surgery (S3 Table).

The cohort included data from 129 surgeons with an average of 149 procedures per surgeon (Table 1). The acuity of the patient population was high, as 46% of surgeries were categorized as either non-elective or were associated with emergent or urgent hospital admission. Among the cohort, 62% of patients required ICU admission > 48 hours. The cohort had a median ICU length of stay of four days (25^th^-75^th^ percentiles two-nine days) and a median hospital length of stay of eleven days (25^th^-75^th^ percentiles seven-twenty days). The overall mortality was 4.8% at thirty days and 16% at one year after index admission. A wide range of comorbidities was documented on admission with cancer and diabetes mellitus being most prevalent. One fourth of the patients were from rural areas and 12% of the patients resided in neighborhoods with a household income below the poverty level [42]. The prevalence of examined complications ranged from 3% for wound complication to 62% for intensive care unit admission > 48 hours (Table 1 and S3 Table). Acute kidney injury, admission to ICU for > 48 hours, and mechanical ventilation for > 48 hours were the most common complications among all surgeries. The distribution of outcomes and preoperative clinical characteristics did not differ between training and validation cohorts.

**Table 1:** Preoperative clinical characteristics and outcomes of the cohort stratified by the occurrence of acute kidney injury within seven days after surgery.

		Acute Kidney Injury onset within seven days after surgery	
		No	Yes
	Overall cohort (N = 2,911)	(N = 1,748, 60%)	(N = 1,163, 40%)
**Demographic features**			
Age, median (25th-75th)	60 (49, 69)	58 (47, 67)	63 (52, 72)^a^
Male gender, n (%)	1760 (60)	1007 (58)	753 (65) ^a^
Race, n (%)			
White	2374 (82)	1440 (82)	934 (80)
African American	265 (9)	150 (9)	115 (10)
Hispanic	117 (4)	64 (4)	53 (5)
Missing	87 (3)	50 (3)	37 (3)
Other	68 (2)	44 (3)	24 (2)
Primary insurance, n (%)^a^			
Private	1208 (42)	797 (46)	411 (35)
Medicare	1204 (41)	635 (36)	569 (49)
Medicaid	340 (12)	198 (11)	142 (12)
Uninsured	159 (5)	118 (7)	41 (4)
**Socio-economic features**			
Neighborhood characteristics			
Rural area, n (%)	767 (27)	467 (27)	300 (26)
Total population, median (25th-75th)	19162 (10639, 30611)	18931 (10510, 30611)	19287 (11056, 30533)
Median income, median (25th-75th)	34372 (29980, 41410)	34328 (29854, 41410)	34459 (30084, 41410)
Total proportion of African-Americans (%), median (25th-75th)	9.6 (3.9, 17.6)	9.5 (3.9, 16.5)	9.6 (3.7, 19.5)
Total proportion of Hispanic (%), median (25th-75th)	4.3 (2.5, 6.8)	4.3 (2.6, 6.7)	4.1 (2.5, 7.1)
Distance from residency to hospital (km), median (25th-75th)	68 (29, 143)	61 (28, 132)	73 (31, 153)^a^
Population proportion below poverty (%), median (25th-75th)	12.0 (8.2, 17.4)	12.1 (8.3, 17.2)	11.8 (8.0, 17.4)
**Comorbidity features**			
Charlson's comorbidity index (CCI), median (25th-75th)	2 (1, 3)	1 (0, 3)	2 (1, 3)^a^
Chronic kidney disease, n (%)	346 (12)	73 (4)	273 (23)^a^
Cancer, n (%)	418 (15)	303 (17)	115 (10)^a^
Diabetes, n (%)	539 (19)	297 (17)	242 (21)^a^
Chronic pulmonary disease, n (%)	656 (23)	331 (19)	325 (28)^a^
Peripheral vascular disease, n (%)	692 (24)	334 (20)	358 (31)^a^
Cerebrovascular disease, n (%)	248 (9)	154 (9)	94 (8)
Congestive heart failure, n (%)	510 (18)	180 (10)	330 (28)^a^
Myocardial infarction, n (%)	308 (11)	140 (8)	168 (15)^a^
Liver disease, n (%)	393 (14)	170 (10)	223 (20)^a^
**Operative features**			
**Admission**			
Weekend admission, n (%)	472 (16)	255 (15)	217 (19)^a^
Admission source, n (%)^a^			
Outpatient setting	1753 (61)	1105 (64)	648 (56)
Emergency room	583 (20)	362 (21)	221 (19)
Transfer	560 (19)	268 (15)	292 (25)
Admission month (top 3 categories), n (%)			
September	279 (10)	168 (10)	111 (10)
October	273 (9)	174 (10)	99 (9)
June	254 (9)	162 (9)	92 (8)
Number of operating surgeons, n	129	112	82
Number of procedures per operating Surgeon, n (%)^a^			
First rank	422 (15)	181 (10)	241 (21)
Second rank	267 (9)	197 (11)	70 (6)
Third rank	258 (9)	129 (7)	129 (11)
Admitting service, n (%)^a^			
Surgery	2545 (87)	1588 (91)	957 (82)
Medicine	366 (13)	160 (9)	206 (18)
Emergent surgery, n (%)	1352 (46)	748 (43)	604 (52)^a^
Time between admission and operation (days)	0 (0, 2)	0 (0, 1)	1 (0, 4)^a^
Surgery type, n (%)^a^			
Cardiothoracic Surgery	1415 (49)	676 (39)	739 (64)
Non-Cardiac General Surgery	952 (33)	614 (35)	338 (29)
Neurologic Surgery	301 (10)	271 (16)	30 (3)
Specialty Surgeries^b^	243 (8)	187 (11)	56 (5)
**Admission day medications**			
Diuretics	600 (21)	285 (16)	315 (27)^a^
Bicarbonate	295 (10)	144 (8)	151 (13)^a^
Angiotensin-Converting-Enzyme Inhibitors	351 (12)	182 (10)	169 (15)^a^
Antiemetic	1413 (49)	946 (54)	467 (40)^a^
Betablockers	872 (30)	513 (29)	359 (31)
Statin	502 (17)	272 (16)	230 (20)^a^
Vasopressors or inotropes	424 (15)	201 (12)	223 (20)^a^
**Outcomes, n (%)**			
Acute kidney injury			
Onset within 3 days of surgery	998 (34)		998 (86)
Onset within 7 days of surgery	1163 (40)		1163 (100)
At any time after surgery	1339 (46)	176 (10)	1163 (100)
Worst stage of acute kidney injury^c^			
Stage 1	695 (52)		685 (59)
Stage 2	303 (23)		237 (20)
Stage 3	338 (25)		238 (20)
Renal replacement therapy	154 (12)		142 (12)
Intensive care unit admission > 48 hours	1800 (62)	894 (51)	906 (78)^a^
Mechanical ventilation > 48 hours	833 (29)	322 (18)	511 (44)^a^
Sepsis	266 (9)	99 (6)	167 (14)^a^
Wound complications	73 (3)	29 (2)	44 (4)^a^
Cardiovascular complications	425 (15)	168 (10)	257 (22)
Venous thromboembolism	133 (5)	61 (4)	72 (6)^a^

^a^ p-value < 0.05 when comparing patients with and without acute kidney injury.

^b^ Specialty surgery includes urological, orthopedics, gynecological, ear nose throat surgeries, ophthalmology and plastic surgery.

^c^ Percentages are among patients with acute kidney injury. For overall cohort, numbers for acute kidney injury at any time during hospitalization are reported. Stage 3 includes cases with and without dialysis.

### Intraoperative physiological time series and risk of acute kidney injury

Patients with AKI had more profound and persistent intraoperative hemodynamic changes. A comparison of statistical variables extracted from intraoperative physiological time series data showed that patients with AKI had significantly higher maximum values, greater short- and long-term variability, lower minimum values, and lower average base signals for systolic, diastolic, and mean arterial pressure (MAP) (Table 2). Similar trends were observed for heart rate, except for average base signal, which was higher in patients with AKI. The only significant variables for minimum alveolar concentration were maximum value and long-term variability, which were higher in AKI patients. There were similar patterns for the secondary outcomes with AKI onset during the first three days after surgery and postoperative AKI onset during any point of the hospitalization for the index surgery (S3 Table).

**Table 2:** Intraoperative clinical characteristics of the cohort stratified by the occurrence of acute kidney injury within seven days after surgery.

		Acute Kidney Injury onset within seven days after surgery	
		No	Yes
	Overall cohort (N = 2,911)	(N = 1,748, 60%)	(N = 1,163, 40%)
**Physiologic intraoperative time series variables, mean (SD)**			
**Systolic Blood Pressure (mmHg)**			
Maximum	225 (34)	224 (35)	227 (32)^a^
Minimum	41 (22)	44 (23)	37 (19)^a^
Average of base signal	107 (17)	110 (17)	102 (16)^a^
Long-term variability	20.27 (6.61)	19.47 (6.51)	21.48 (6.59)^a^
Short-term variability	7.86 (2.63)	7.72 (2.69)	8.07 (2.53)^a^
**Diastolic Blood Pressure (mmHg)**			
Maximum	134 (24)	133 (24)	136 (24)^a^
Minimum	20 (15)	21 (15)	18 (13)^a^
Average of base signal	60 (9)	61 (9)	57 (8)^a^
Long-term variability	11.02 (3.65)	10.83 (3.67)	11.31 (3.6)^a^
Short-term variability	4.89 (1.65)	4.78 (1.68)	5.05 (1.6)^a^
**Mean Blood Pressure (mmHg)**			
Maximum	168 (28)	167 (28)	170 (26)^a^
Minimum	22 (20)	25 (22)	19 (17)^a^
Average of base signal	76 (12)	78 (13)	72 (10)^a^
Long-term variability	14.5 (5.3)	14.2 (5.4)	14.9 (5.2)^a^
Short-term variability	6.0 (2.1)	5.9 (2.1)	6.2 (2.0)^a^
**Heart rate (beats/minute)**			
Maximum	149 (30)	147 (30)	152 (31)^a^
Minimum	38 (21)	41 (21)	34 (20)^a^
Average of base signal	84 (15)	83 (15)	85 (15)^a^
Long-term variability	14.84 (8.4)	13.87 (7.88)	16.29 (8.94)^a^
Short-term variability	5.29 (1.76)	5.12 (1.79)	5.56 (1.69)^a^
**Minimum alveolar concentration (%)**			
Maximum	2.63 (0.89)	2.56 (0.91)	2.73 (0.87)^a^
Minimum	0.02 (0.06)	0.02 (0.06)	0.02 (0.05)
Average of base signal	0.58 (0.17)	0.58 (0.17)	0.58 (0.18)
Long-term variability	0.35 (0.19)	0.33 (0.18)	0.37 (0.2)^a^
Short-term variability	0.09 (0.05)	0.09 (0.05)	0.09 (0.05)
**Intraoperative laboratory results, median (25th-75th)**			
**Arterial Blood Gas Panel**			
**pH**			
Maximum	7.38 (7.33, 7.43)	7.38 (7.34, 7.43)	7.38 (7.32, 7.43)
Mean	7.36 (7.32, 7.4)	7.36 (7.33, 7.41)	7.36 (7.32, 7.4)^a^
Minimum	7.34 (7.3, 7.39)	7.34 (7.31, 7.39)	7.34 (7.3, 7.38)^a^
**Partial pressure of carbon dioxide (mmHg)**			
Maximum	42.8 (38.4, 47.2)	42.8 (38.2, 47.0)	43.1 (38.8, 47.5)
Mean	40.8 (36.9, 45.2)	40.8 (36.7, 45.0)	40.9 (37.2, 45.4)^a^
Minimum	38.9 (34.6, 44.6)	38.8 (34.4, 44.4)	39.5 (34.8, 44.8)^a^
**Ratio of partial pressure arterial oxygen and fraction of inspired oxygen (mmHg)**	90 (75, 118)	92 (76, 122)	88 (74, 114)^a^
**Arterial Oxygen Content (mL/dL)**			
Maximum	15.3 (14.0, 16.9)	15.4 (14.1, 17.0)	15.2 (13.8, 16.9)^a^
Mean	14.7 (13.4, 16.3)	14.8 (13.5, 16.4)	14.5 (13.2, 16.1)^a^
Minimum	14.2 (12.5, 16.0)	14.3 (12.8, 16.1)	14.0 (12.3, 15.8)^a^
**Carboxyhemoglobin (%)**			
Maximum	2.2 (1.4, 3.0)	2.1 (1.3, 2.9)	2.3 (1.5, 3.1)^a^
Mean	2.0 (1.3, 2.8)	1.9 (1.2, 2.7)	2.1 (1.4, 2.9)^a^
Minimum	1.6 (1.1, 2.6)	1.5 (1.0,2.5)	1.8 (1.2, 2.7)^a^
**Methemoglobin (%)**			
Maximum	0.8 (0.6, 1.0)	0.8 (0.6, 0.9)	0.8 (0.6, 1.0)^a^
Mean	0.7 (0.5, 0.9)	0.7 (0.5, 0.8)	0.7 (0.6, 0.9)^a^
Minimum	0.6 (0.4, 0.8)	0.6 (0.4, 0.8)	0.6 (0.4, 0.9)^a^
**Complete Blood Count Panel**			
**White blood cells (thou/mm**^**3**^**)**			
Maximum	13.6 (9.9, 18.3)	13.3 (9.9, 17.8)	14.0 (9.8, 19.3)^a^
Mean	12.9 (9.3, 17.5)	12.5 (9.3, 16.9)	13.3 (9.2, 18.3)^a^
Minimum	12.1 (8.5, 16.9)	11.7 (8.5, 16.3)	12.6 (8.4, 17.7)^a^
**Red blood cells (million/mcL)**			
Maximum	3.59 (3.23, 3.97)	3.6 (3.25, 3.98)	3.57 (3.22, 3.94)
Mean	3.5 (3.16, 3.87)	3.52 (3.19, 3.9)	3.46 (3.12, 3.85)^a^
Minimum	3.42 (3.07, 3.83)	3.45 (3.11, 3.85)	3.38 (3.0, 3.8)^a^
**Hemoglobin (g/dL)**			
Maximum	10.8 (9.8,12.0)	10.8 (9.9,12.0)	10.8 (9.8,11.9)
Mean	10.3 (9.3,11.6)	10.45 (9.3,11.7)	10.2 (9.2,11.4)^a^
Minimum	10.6 (9.6,11.7)	10.6 (9.7,11.8)	10.5 (9.6,11.5)^a^
**Hematocrit (%)**			
Maximum	31.5 (28.7, 34.9)	31.6 (28.8, 35.5)	31.4 (28.5, 34.8)
Mean	30.8 (28.0, 34.1)	30.9 (28.2, 34.3)	30.6 (27.8, 33.8)^a^
Minimum	30.2 (27.1, 33.6)	30.5 (27.4, 33.9)	29.9 (26.7, 33.3)^a^
**Red cell distribution width (%)**			
Maximum	14.9 (13.9, 16.1)	14.6 (13.7, 15.7)	15.3 (14.4, 16.7)^a^
Mean	14.8 (13.8, 15.9)	14.5 (13.6, 15.5)	15.2 (14.4, 16.5)^a^
Minimum	14.7 (13.8,15.9)	14.4 (13.6, 15.5)	15.1 (14.2, 16.4)^a^
**Platelet count (thou/mm**^**3**^**)**			
Maximum	180 (133, 239)	197 (145, 253)	163 (117, 209)^a^
Mean	176 (128, 229)	190 (140, 245)	156 (110, 203)^a^
Minimum	171 (121, 223)	184 (135, 240)	151 (104, 198)^a^
**Lactic acid (mmol/L)**			
Maximum	2.5 (1.5, 4.2)	2.1 (1.3, 3.3)	3.4 (2.0, 5.2)^a^
Mean	2.2 (1.4, 3.6)	1.8 (1.2, 2.8)	2.8 (1.8, 4.6)^a^
Minimum	1.6 (1.1, 2.9)	1.4 (1.0, 2.4)	2.2 (1.3, 4.0)^a^
**Mean Platelet Volume (fL)**			
Maximum	8.0 (7.5, 8.7)	7.9 (7.4, 8.5)	8.2 (7.6, 8.8)^a^
Mean	7.9 (7.4, 8.5)	7.8 (7.3, 8.4)	8.1 (7.5, 8.7)^a^
Minimum	7.8 (7.3, 8.4)	7.7 (7.2, 8.3)	7.9 (7.4, 8.6)^a^
**Intraoperative medications, n (%)**			
Total administered intravenous fluids (mL)	2300 (1400, 3600)	2435 (1500, 3700)	2200 (1250, 3500)^a^
Total administered blood products (mL)	318 (0, 1250)	0 (0, 750)	750 (0, 2500)^a^
Diuretic (vs. No)	329 (11)	119 (7)	210 (18)^a^
Vasopressors (vs. No)	1942 (67)	1084 (62)	858 (74)^a^
**Other variables**			
General anesthesia, n (%)	2881 (99)	1728 (100)	1153 (99)
Duration of surgery (minute), median (25th-75th)	387 (294, 483)	357 (271, 449)	425 (340, 521)^a^
Surgery performed between 7 pm and 7 am, n (%)	394 (14)	210 (12)	184 (16)^a^
Total estimated blood loss (mL)	150 (150, 500)	150 (150, 500)	150 (150, 350)^a^`
Total urine output (mL)	650 (300, 1150)	600 (300, 1100)	700 (350, 1200)^a^

^a^ p-value < 0.05 when comparing patients with and without acute kidney injury.

**Fig 3:**
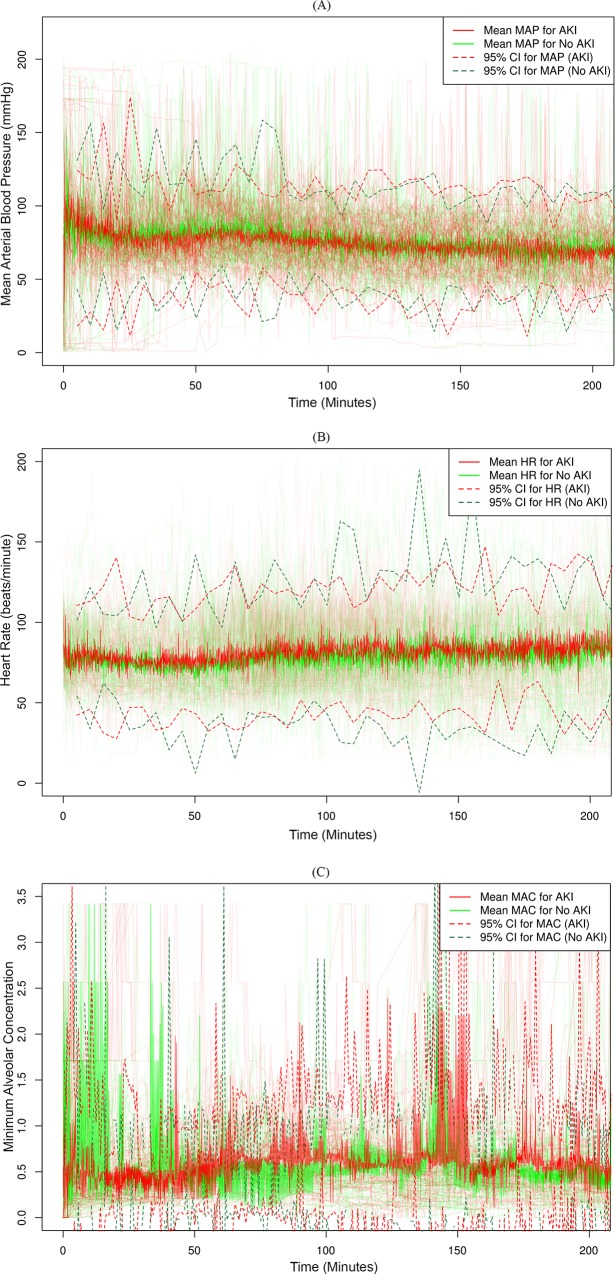
Intraoperative physiological time series variations stratified by the occurrence of acute kidney injury. Intraoperative physiological time series variations for 100 randomly selected patients during the first 200 minutes of surgery stratified by the occurrence of acute kidney injury (AKI) within the first seven days after surgery. Mean and 95% Confidence Interval (CI) stratified by the occurrence of AKI are shown for (A) intraoperative mean arterial blood pressure, MAP (mmHg), (B) intraoperative heart rate, HR (beats/minute), and (C) intraoperative mean alveolar concentration of anesthetics, MAC.

Patients with AKI had greater variation in physiological parameters measured during surgery (Fig 3).

**Fig 4:**
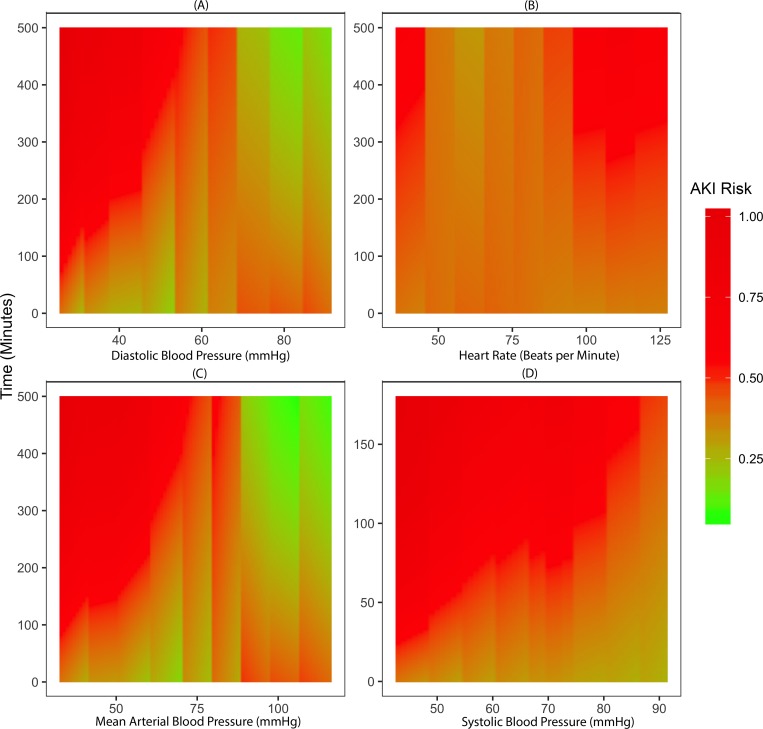
Association between risk of postoperative acute kidney injury and magnitude and duration of intraoperative physiological variations. The risk for the development of postoperative acute kidney injury (AKI) within the first seven days after surgery, as predicted by the postoperative stacked model, was aggregated across the entire cohort. The color represents the risk of developing postoperative AKI, where red is high-risk, and green is low-risk. The y-axis is time (minutes) and the x-axes are (A) diastolic blood pressure (mmHg), (B) systolic blood pressure (mmHg), (C) Mean arterial blood pressure (mmHg), and (D) Heart rate (beats/minute).

The severity and duration of intraoperative hypotension was directly correlated with the risk of developing postoperative AKI (Fig 4). Patients with prolonged periods of blood pressure within the low range of normal values, yet above the traditional threshold for hypotensive treatment (60–65 mmHg), still carried a higher risk for AKI (Fig 4A). Similarly, persistently elevated heart rate was associated with the risk of AKI (Fig 4D).

### Other intraoperative variables and risk of acute kidney injury

**Fig 5:**
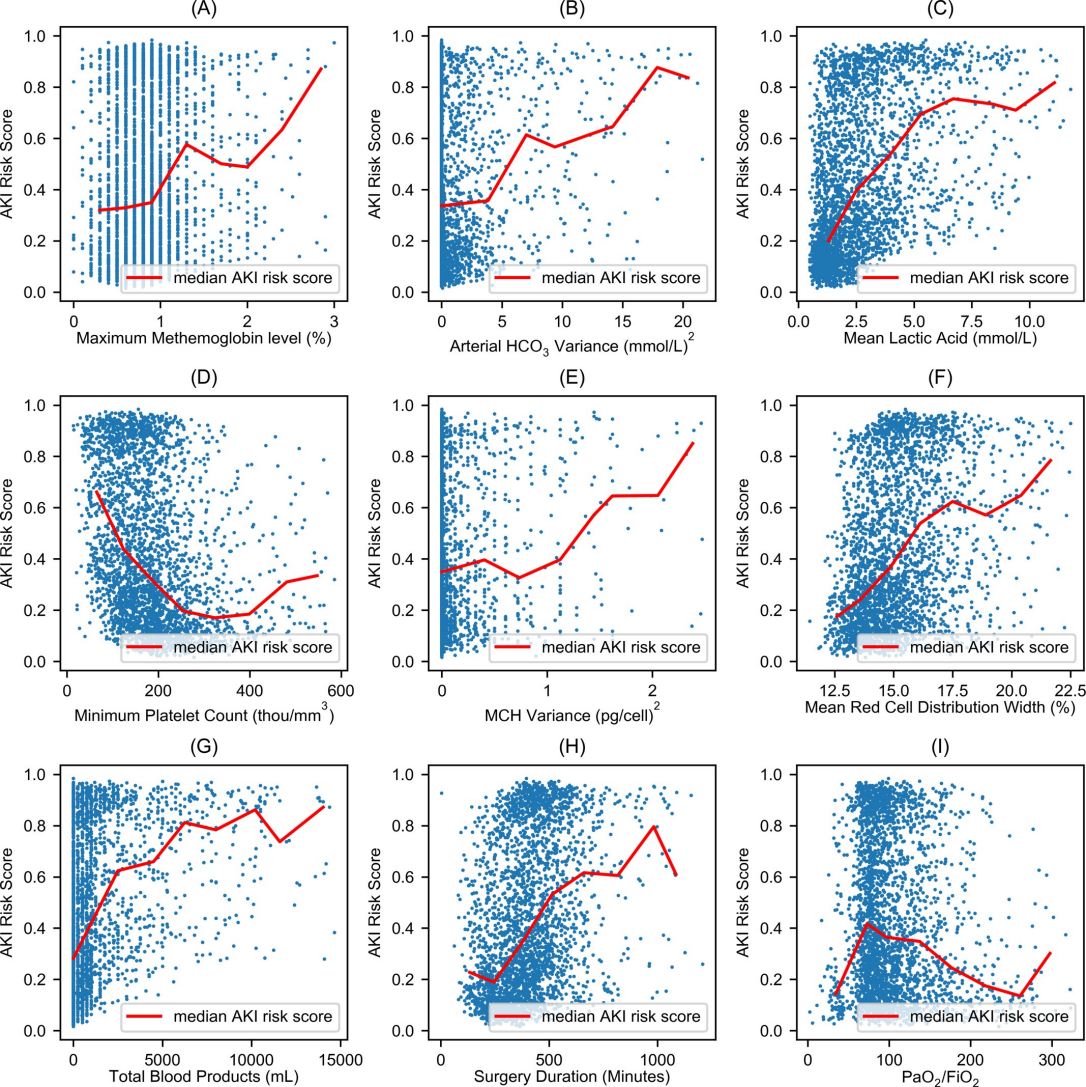
Nonlinear association between risk of postoperative acute kidney injury and intraoperative features. Each blue dot represents a patient that developed postoperative AKI. The y-axis represents the risk probability for postoperative acute kidney injury, which ranges from 0 to 1. The red line represents the median acute kidney injury risk score given the values of x. The x-axes represent (A) maximum intraoperative percentage of methemoglobin in arterial blood gasses, (B) intraoperative variance of bicarbonate (mmol/L)^2^ in arterial blood gasses, (C) intraoperative mean of lactic acid (mmol/L), (D) minimum intraoperative platelet count (thou/mm^3^), (E) intraoperative variance of mean corpuscular hemoglobin, MCH (pg/cell)^2^, (F) intraoperative mean of red cell distribution (%), (G) total of blood products administered during surgery (mL), and (H) duration of surgery (minutes).

The following intraoperative arterial blood gas panel measurements were found to be significantly higher in patients that developed AKI and were correlated with increased risk of developing AKI: partial pressure of carbon dioxide, percentage of carboxyhemoglobin, percentage of methemoglobin, and variance in bicarbonate. Additionally, arterial oxygen content was found to be significantly lower in AKI patients (Table 2 and S3 Table). Among other intraoperative laboratory tests, white blood cell count, red cell distribution width, and lactic acid were significantly higher in patients who developed AKI, while platelet count, red blood cell count, hemoglobin, and hematocrit were significantly lower in AKI patients (Table 2 and S3 Table). The correlation between important features and AKI risk probability was not linear (Fig 5). The total amount of blood products administered during surgery was significantly associated with progressive increases in AKI risk probability (Fig 5G). Patients who developed AKI were more likely to receive diuretics (18% vs 7%) and vasopressors (74% vs 62%) during surgery and were administered less intravenous fluid intraoperatively (Table 2 and S3 Table). The duration of surgery was significantly higher in patients with AKI and was highly correlated with the risk for AKI (Fig 5H). Interestingly, patients with AKI were more likely to have their operation performed between 7 pm and 7 am. Similar trends were observed for the secondary outcomes: AKI with the onset in the first three days of surgery and postoperative AKI occurring at any time during the hospitalization for the index surgery (S3 Table).

### Risk score stratification and model performance

**Fig 6:**
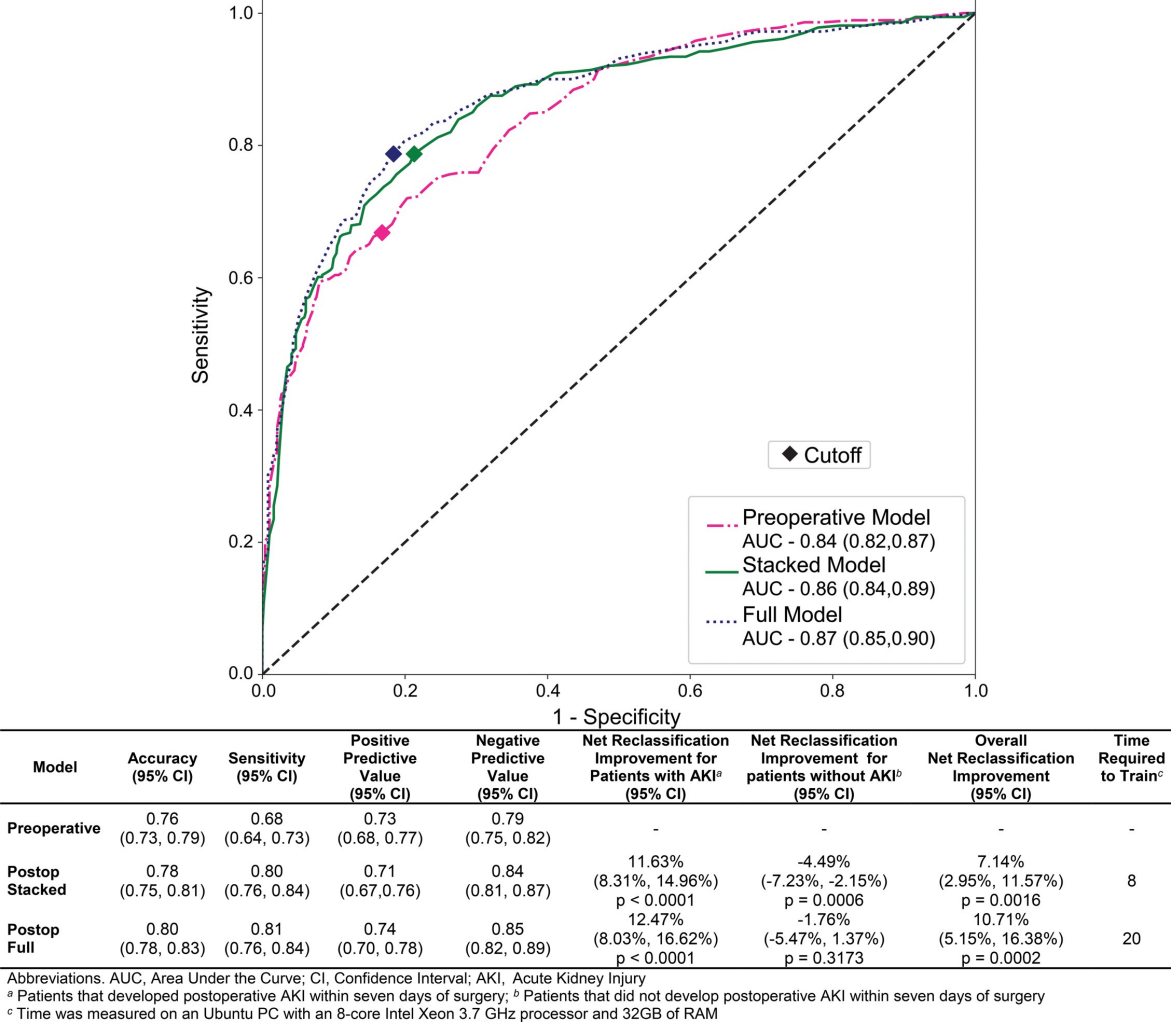
Receiver operating characteristic curves and other performance metrics for prediction of postoperative acute kidney injury. The graph at the top of the figure is the receiver operating characteristic curves for prediction of postoperative acute kidney injury (AKI) within the first seven days after surgery for the preoperative model (pink), the postoperative stacked model (green), and the postoperative full model (blue). The diamonds on each curve represent the cutoffs calculated by maximizing the Youden Index. The y-axis is sensitivity, which ranges from zero to one. The x-axis is one minus specificity, which ranges from zero to one. The table in the bottom of the figure contains the following performance metrics for the preoperative and both postoperative models: (A) accuracy (percentage of patients correctly classified), (B) sensitivity (percentage of patients that developed AKI that were classified as high-risk), (C) positive predictive value (percentage of high-risk patients that developed AKI), (D) negative predictive value (percentage of low-risk patients that did not develop AKI), (E) net reclassification improvement for patients that developed postoperative AKI (F) net reclassification improvement for patients that did not develop postoperative AKI, (H) overall net reclassification improvement.

The *IDEA* algorithm calculated the risk for developing AKI (ranging from 0 to 1) at two distinct time points: once preoperatively using only preoperative data and once immediately after surgery after enriching the preoperative data with physiological responses to surgery and intraoperative events. The algorithm automatically determined the optimal threshold for stratifying patients into low- and high-risk groups (Fig 2 and S2 Fig). The predictive performance for all three models in the validation dataset was very good (Fig 6) and both of the postoperative models (stacked model AUC 0.86, 95% CI 0.84–0.89 and full model AUC 0.87, 95% CI 0.85–0.90) performed better than the preoperative model (AUC 0.84, 95% CI 0.82–0.87). The sensitivity increased from 0.68 (95% CI 0.64–0.73) in the preoperative model to 0.81 (95% CI 0.76–0.84) in the full postoperative model. Even though the positive predictive values were in the same range for the preoperative and the postoperative models (0.68 to 0.78), the negative predictive value of the postoperative models (0.85, 95% CI 0.82–0.88) was significantly improved compared to the preoperative model (0.79, 95% CI 0.75–0.82). The data transformation step required 48 hours, training the postoperative full model required 20 hours, and training the postoperative stacked model required 8 hours. The data transformation and training were done on an Ubuntu PC with an Intel Xeon 3.7GHz Processor with 8 cores and 32GB RAM. The predictive performance for the two secondary outcomes (AKI onset within first three postoperative days and AKI onset before discharge) were similar and demonstrated the same trends as the primary outcome postoperative models, with AUC values ranging between 0.82 and 0.88 (S4 Table).

Most significantly, according to the feature importance scores from the trained postoperative random forest models (S5 Table), only three of the top ten important features were preoperative variables (zip code, chronic kidney disease, and attending surgeon) while all other important features were derived from the following intraoperative variables: lactic acid, total blood products administered during surgery, red cell distribution width, and diastolic blood pressure (S5 Table).

### Reclassification of risk groups with intraoperative data

The observed absolute risk (the percentage of patients in a risk group that developed AKI within the first seven days after surgery) was distinctly different between the low- and high-risk groups for the preoperative model and both postoperative models (Table 3). The relative risk (the ratio of the absolute risk between the high- and low-risk groups) was significantly higher for the postoperative stacked and full models, 4.6 (95% CI 3.7–5.7) and 5.1 (95% CI 4.1–6.4) respectively, compared to the preoperative model, 3.4 (95%CI 2.8–4.0).

**Table 3:** Absolute and relative risks associated with high- and low-risk groups for acute kidney injury onset within the first seven days after surgery stratified by predictive model.

	Absolute risk % ^a^ (95% Confidence Interval)		Relative risk ^b^ (95% Confidence Interval)
Models	Low-risk group^c^	High-risk group^c^	High- vs low-risk group
Preoperative model	21.5% (18.1%, 25.02%)	72.6% (67.8%, 77.3%)	3.4 (2.8, 4.0)
Postoperative stacked model	15.6% (12.3%, 18.9%)^d^	71.3% (66.9%, 75.7%)	4.6 (3.7, 5.7)
Postoperative full model	14.6% (11.4%, 17.7%)^d^	74.1% (69.7%, 78.4%)	5.1 (4.1, 6.4)

^a^ Absolute risk was calculated as the percentage of cases for which acute kidney injury occurred in the low- and high-risk groups, respectively.

^b^ Relative risk was calculated as the ratio of the absolute risk of the occurrence of acute kidney injury between the high- and low-risk groups.

^c^ Patients were classified as low-risk if their prediction score was less than or equal to cutoff and high-risk otherwise. The cutoff values were 0.43, 0.41, and 0.40 for preoperative model, postoperative stacked model, and postoperative full model, respectively. The cutoffs were determined using the maximum value of Youden Index.

^d^ Significantly different from preoperative model with adjusted p-value ≤ 0.05 when absolute risk of all pairs of three models were compared using the Bonferroni method.

**Fig 7:**
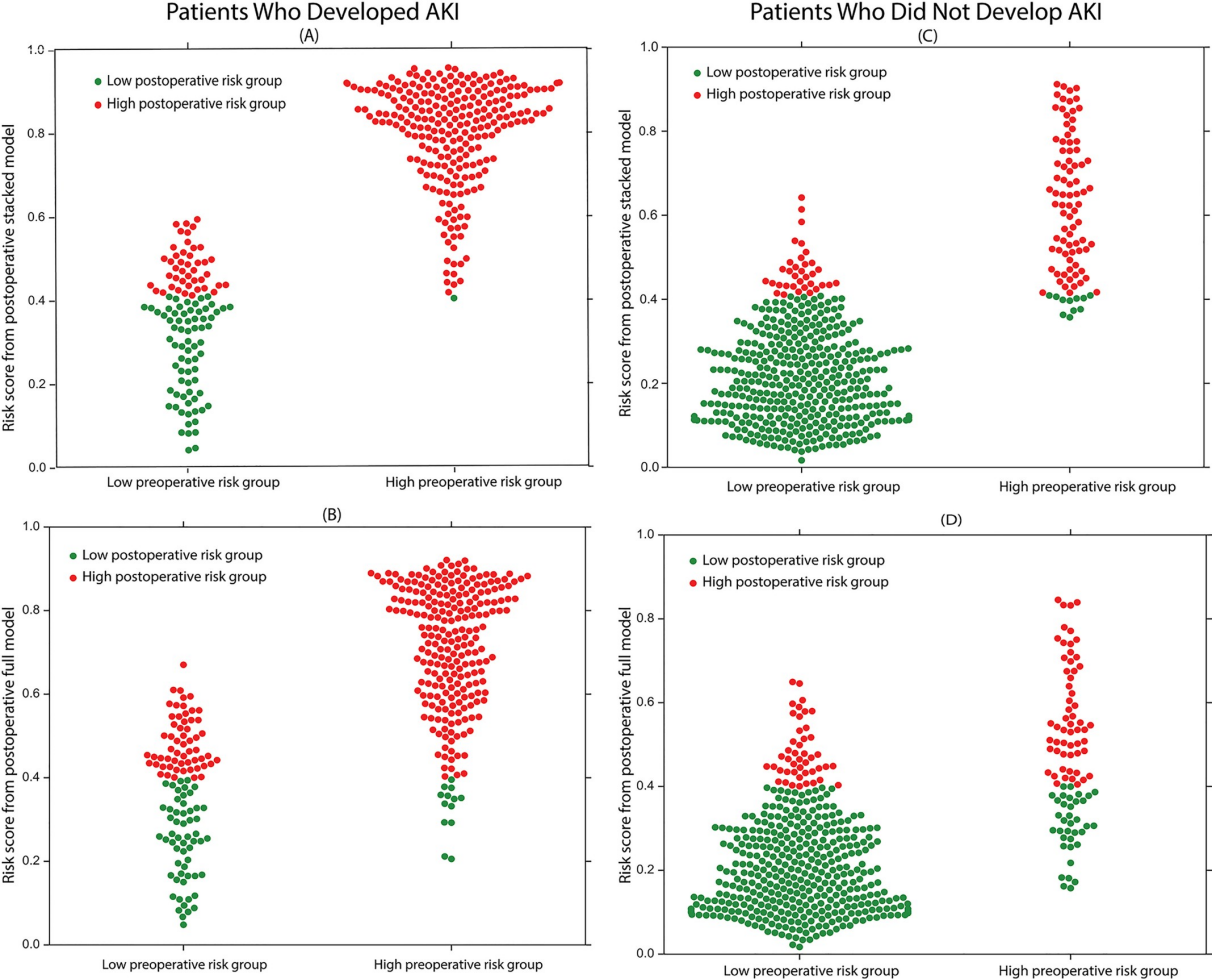
Reclassification performance of the postoperative stacked and full models for predicting postoperative acute kidney injury. The postoperative risk scores in the first column are from the stacked model, while the postoperative risk scores for the second column are from the full model. The patients in the first row developed postoperative AKI within seven days of surgery, whereas the patients in the second row did not develop postoperative AKI within seven days of surgery. The y-axis on each plot is the postoperative model acute kidney injury risk score, which ranges from zero to one. The x-axis on each plot is the preoperative risk group. The red dots are patients at high-risk for AKI according to the postoperative model, whereas the green dots are patients at low-risk for AKI according to the postoperative model. (A & B) The proposed postoperative stacked and full models effectively reclassified false negative patients from the preoperative model as high AKI risk patients.

To assess the incremental improvement of classifying patients into the correct risk groups by the addition of intraoperative data, the net reclassification improvement (NRI) was calculated for both the stacked and full postoperative models. The calculated NRI (net percentage of correctly reclassified cases after addition of the intraoperative data) showed a statistically significant improvement for both the stacked and the full postoperative models with 7% (95% CI (3%, 12%), p < 0.005) and 11% (95% CI (5%, 16%), p < 0.0005) of cases correctly reclassified, respectively (Fig 6). The NRI for patients that developed postoperative AKI within seven days after surgery was approximately 12% (95% CI (8%, 15%), p <0.0001) for both postoperative models, while the NRI for patients that did not develop postoperative AKI was -4% (95% CI (-4%, -7%), p<0.001) and -2% (95% CI (-5%, 1%), p>0.5) for the stacked and full models, respectively. Both postoperative models were able to reclassify approximately 40% of the false negative patients from the preoperative model correctly into the high-risk group (Fig 7). This demonstrates that intraoperative data can be used as a significant predictor to correctly assess patients’ increased risk for postoperative AKI within seven days of surgery (compared to preoperative risk). However, the addition of intraoperative data is unlikely to accurately lower patients’ risk for the development of postoperative AKI within seven days of surgery. The secondary outcomes, postoperative AKI onset within three days of surgery and postoperative AKI onset before discharge, also showed statistically significant NRIs comparing the postoperative models to the preoperative model. However, for the outcome predicting development of postoperative AKI within three days of surgery, both postoperative models were more effective at reclassifying false positive patients from the preoperative model compared to false negative patients (S3 Fig and S4 Table).

**Fig 8:**
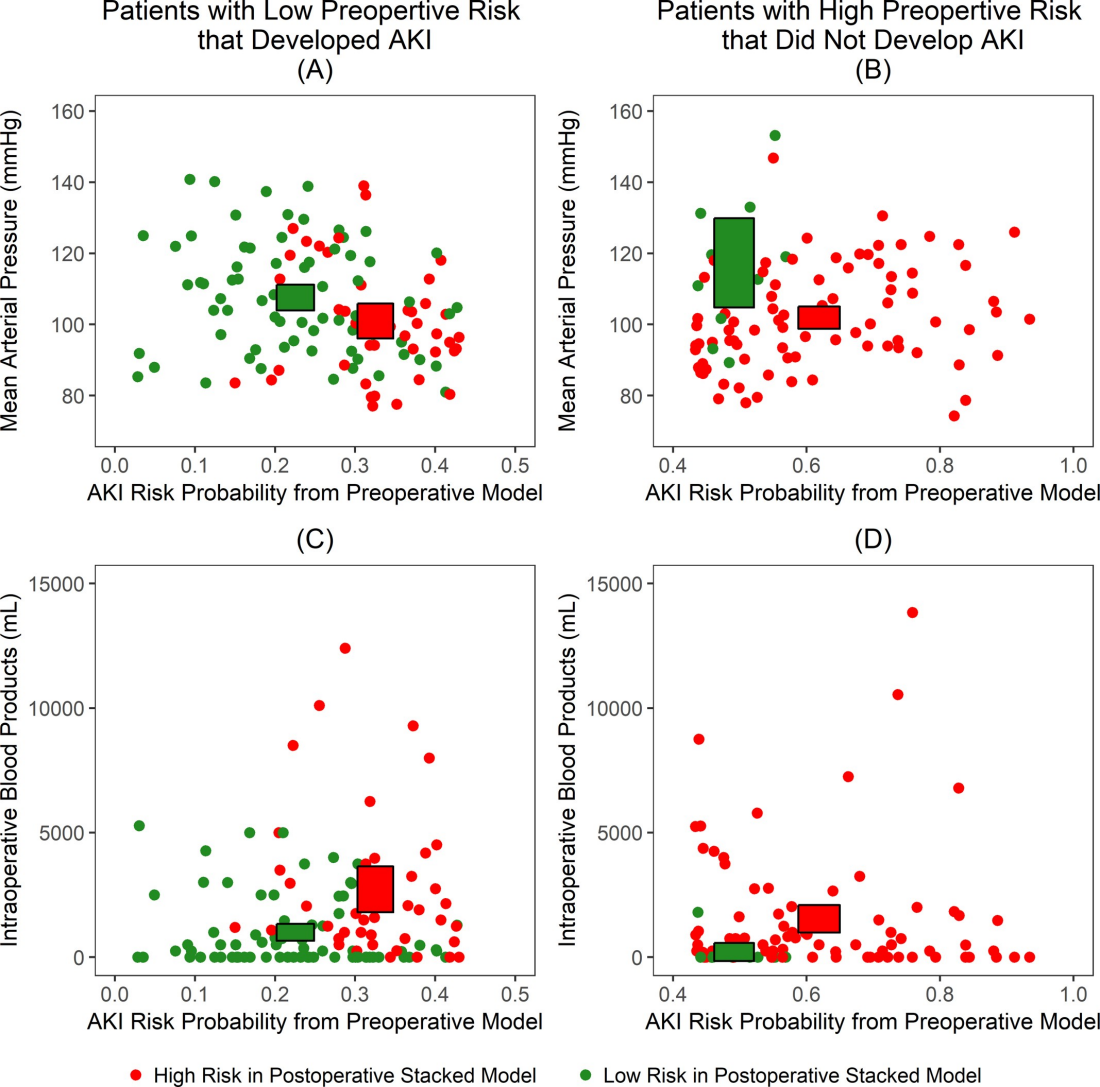
Association between intraoperative variables (mean arterial pressure and administered blood products) and postoperative risk group reclassification. The first column represents patients that developed postoperative acute kidney injury (AKI), yet were classified as low-risk by the preoperative model. The second column represents patients that did not develop postoperative AKI, but were classified as high-risk by the preoperative model. The boxes represent 95% confidence intervals for the given variables. (A) Patients correctly classified as having high-risk for AKI by the postoperative stacked model tended to have lower mean arterial pressure. (B) Patients correctly classified as having low-risk for AKI by the postoperative stacked model tended to have higher mean arterial pressure. (C) Patients correctly classified as having high-risk for AKI by the postoperative stacked model tended to have greater volumes of administered intraoperative blood products. (D) Patients correctly classified as having low-risk for AKI by the postoperative stacked model tended to have lesser volumes of administered intraoperative blood products.

Furthermore, the patients in the low preoperative risk group for postoperative AKI who developed AKI within seven days of surgery that were correctly reclassified into the high-risk group by the postoperative models, had lower intraoperative mean arterial blood pressure values and higher intraoperative blood product volumes compared to those who remained in the low-risk group (Fig 8). Conversely, the patients in the high preoperative risk group for postoperative AKI who did not develop AKI within seven days of surgery that were correctly reclassified into the low-risk group by the postoperative models, had higher intraoperative mean blood pressure values and lower intraoperative blood product volumes compared to those who remained in the high-risk group.

## Discussion

Using a large single-center cohort of surgical patients, we developed and validated a dynamic machine-learning algorithm that readjusts the preoperative risk for postoperative AKI using physiological time series data and other data collected during surgery to provide a personalized risk panel for acute kidney injury with both preoperative and immediate postoperative risk assessments. This work expands on our previously validated *MySurgeryRisk* algorithm which predicts preoperative risk for major postoperative complications, including AKI [18], to leverage temporal enrichment of the preoperative model with the new information related to patients’ changes in physiological status during surgery. The advantages of the algorithm include a) prediction entirely based on routinely available preoperative and intraoperative data, b) universal applicability to any surgical context, c) exportability to other EHR systems, and d) the ability to handle any data type in EHR (including time series and sparse data). Most importantly, the dynamic reassessment of the risk for postoperative AKI using temporal enrichment with intraoperative data allows for more precise reclassification of AKI risk based on how patients’ clinical trajectory progresses. While preoperative models mainly asses risk based on patients’ pre-existing health conditions and general risks associated with the type of planned procedures, the addition of intraoperative time series data reflects acute physiological responses to the stresses of surgery, which provides a better risk assessment for postoperative AKI to a physician who would want to use the model. For example, a patient without significant comorbidities who undergoes a moderate risk surgery would have low-risk for postoperative AKI based on a preoperative model. If that patient was to develop one or more complications during surgery, such as severe bleeding, adverse reaction to anesthetics, or treatment with nephrotoxic drugs, his/her physiological responses captured in the intraoperative data would reclassify him/her to the high-risk group. A change in classification such as this would be extremely valuable for a physician. The *IDEA* algorithm demonstrated the ability to integrate intraoperative data that not only resulted in an improved AUC compared to the preoperative model, but resulted in effectively reclassifying up to 40% of patients from the preoperative model into a new risk category based on intraoperative events.

While several preoperative factors, such as age, comorbidity, chronic kidney disease, and type of surgery have been identified as risk factors for AKI based on previous studies [43], the recognition that certain types of admission medications, or even the timing of the operation itself, are risk factors for AKI confirms some recent observations [44–46]. Furthermore, the dynamic changes in intraoperative blood pressure and heart rate were among the most important features contributing to the risk of developing postoperative AKI in our model. This indicates that the duration and magnitude of intraoperative hypotension as well as concomitant changes in heart rate significantly increase the risk for postoperative AKI. Intraoperative hypotension is a well-recognized risk factor for multiple postoperative complications [47–49], with biological plausibility that is well accepted among anesthesiologists and surgeons, yet no consensus exists regarding the optimal blood pressure target to support the perfusion of critical organs during surgery [50, 51]. The red cell distribution width, platelet count, and the duration of operation were among the most important intraoperative predictors for postoperative AKI, and while some of them have been previously reported as risk factors for AKI in other patient populations, this is the first report for surgical patients [52–54].

Most of the existing risk models for acute kidney injury are focused on the general hospital population [10, 11] or are designed for critically ill patients [15]. Many of the previous risk models for surgical AKI were limited to either a specific type of surgery or the use of preoperative risk factors for statistical modeling (mainly logistic regression) with reported AUCs between 0.77 and 0.84 [1, 2, 55, 56]. The risk prediction models that have incorporated intraoperative variables are scarce and mainly focused on patients undergoing cardiac surgery with reported AUCs ranging between 0.72 and 0.81 [13, 14, 57]. The fact that these models did not fully utilize the available time-varying physiological data during the surgery, or machine learning approach, may have contributed to their lower performance compared to our model.

This study has some limitations. Firstly, only the first surgery was used for patients that underwent multiple surgeries for building our proposed predictive models. Secondly, the algorithm was only provided with the data and outcomes in the training dataset, without explicit definitions of features. Thirdly, because the algorithm ‘‘learned” the features that were most predictive for the risk of developing postoperative AKI implicitly, it is possible that the algorithm is using features previously unknown to or ignored by physicians. Fourthly, the expansion of input features to include operative notes may increase the accuracy, but will require more elaborate computational approaches. Lastly, the algorithm has been trained to capture practice patterns for individual providers in the referral population of a large academic medical center in North Central Florida. Further training and validation of the algorithm is necessary in a dataset with different population characteristics and practice patterns.

## Conclusions

In a large single-center cohort of surgical patients, our proposed Intraoperative Data Embedded Analytics (*IDEA*) algorithm employed a machine learning approach based on a random forest classifier to improve patients’ postoperative acute kidney injury (AKI) risk prediction with high sensitivity and specificity by utilizing intraoperative data. The *IDEA* algorithm was able to correctly reclassify approximately 40% of patients who were considered low-risk for postoperative AKI by preoperative model to high-risk. This illustrates the importance of intraoperative data in AKI risk stratification. Given the association between AKI and increased morbidity, mortality, and cost, it is important for clinicians to have dynamic AKI risk prediction algorithms capable of adjusting AKI risk as new information becomes available. Further research can address other post-surgical complications as well as validation of the proposed algorithm on external datasets.

## Supporting information

S1 Methods (DOCX)

S1 Table Checklist for TRIPOD statement. (DOCX)

S2 Table Characteristics of input variables. (DOCX)

S3 Table Summary of clinical characteristics of the cohort and outcomes stratified by each of the acute kidney injury outcomes. (DOCX)

S4 Table Model performances for the secondary acute kidney injury outcomes. (DOCX)

S5 Table Important features from the postoperative models. (DOCX)

S1 Fig Model training flow diagram. The cohort of size 2,911 with 231 features was randomly split into training (70%) and testing (30%) cohorts. A random forest classifier was used to train the AKI prediction model (we used 5-fold cross validation for hyperparameter tuning and feature selection) for all three outcomes separately and performance was tested using the testing (validation) cohort.
(TIF)

S2 Fig Performance plots for the postoperative stacked model. The first column shows the optimization of the cutoff threshold by maximizing the Youden Index. The second column shows the relationship between the performance metrics (accuracy, positive predictive value, negative predictive value, and Youden Index) and threshold. (A & B) The first row is for the prediction of postoperative acute kidney Injury (AKI) within three days of surgery outcome. (C & D) The second row is for the prediction of postoperative AKI within seven days of surgery outcome. (E & F) The third row is for the prediction of postoperative AKI prior to discharge outcome.
(TIF)

S3 Fig Reclassification performance for the secondary outcomes. The first row represents the reclassification of (A) patients that developed postoperative acute kidney injury (AKI) within three days of surgery and (B) patients that did not develop postoperative AKI within three days of surgery for the three day secondary outcome. The second row represents the reclassification of (C) patients that developed postoperative AKI before discharge and (D) patients that did not develop postoperative AKI before discharge for the overall secondary outcome.
(TIF)

## References

[1] Hobson C, Singhania G, Bihorac A. Acute Kidney Injury in the Surgical Patient. Crit Care Clin. 2015;31(4):705–23. Epub 2015/09/28. 10.1016/j.ccc.2015.06.007 26410139 PMC4584402

[2] Hobson C, Lysak N, Huber M, Scali S, Bihorac A. Epidemiology, outcomes, and management of acute kidney injury in the vascular surgery patient. J Vasc Surg. 2018;68(3):916–28. Epub 2018/08/28. 10.1016/j.jvs.2018.05.017 30146038 PMC6236681

[3] Bihorac A, Yavas S, Subbiah S, Hobson CE, Schold JD, Gabrielli A, et al Long-term risk of mortality and acute kidney injury during hospitalization after major surgery. Annals of Surgery. 2009;249(5):851–8. Epub 2009/04/24. 10.1097/SLA.0b013e3181a40a0b . 19387314

[4] Hobson C, Ozrazgat-Baslanti T, Kuxhausen A, Thottakkara P, Efron PA, Moore FA, et al Cost and Mortality Associated With Postoperative Acute Kidney Injury. Ann Surg. 2015;261(6):1207–14. Epub 2014/06/03. 10.1097/SLA.0000000000000732 24887982 PMC4247993

[5] Huber M, Ozrazgat-Baslanti T, Thottakkara P, Efron PA, Feezor R, Hobson C, et al Mortality and Cost of Acute and Chronic Kidney Disease after Vascular Surgery. Ann Vasc Surg. 2016;30:72–81 e1-2. 10.1016/j.avsg.2015.04.092 26187703 PMC4691411

[6] Huber M, Ozrazgat-Baslanti T, Thottakkara P, Scali S, Bihorac A, Hobson C. Cardiovascular-specific mortality and kidney disease in patients undergoing vascular surgery. JAMA surgery. 2016;151(5):441–50. 10.1001/jamasurg.2015.4526 26720406 PMC4871734

[7] Ozrazgat-Baslanti T, Thottakkara P, Huber M, Berg K, Gravenstein N, Tighe P, et al Acute and Chronic Kidney Disease and Cardiovascular Mortality After Major Surgery. Annals of surgery. 2016. 10.1097/SLA.0000000000001582 PMC4936961 26756753

[8] Hobson C, Ozrazgat-Baslanti T, Kuxhausen A, Thottakkara P, Efron PA, Moore FA, et al Cost and Mortality Associated With Postoperative Acute Kidney Injury. Annals of Surgery. 2015;261(6):1207–14. 10.1097/SLA.0000000000000732 00000658-201506000-00027. 24887982 PMC4247993

[9] Lysak N, Bihorac A, Hobson C. Mortality and cost of acute and chronic kidney disease after cardiac surgery. Curr Opin Anaesthesiol. 2017;30(1):113–7. Epub 2016/11/15. 10.1097/ACO.0000000000000422 27841788 PMC5303614

[10] Koyner JL, Adhikari R, Edelson DP, Churpek MM. Development of a Multicenter Ward-Based AKI Prediction Model. Clin J Am Soc Nephrol. 2016;11(11):1935–43. Epub 2016/09/17. 10.2215/CJN.00280116 27633727 PMC5108182

[11] Hodgson LE, Sarnowski A, Roderick PJ, Dimitrov BD, Venn RM, Forni LG. Systematic review of prognostic prediction models for acute kidney injury (AKI) in general hospital populations. BMJ Open. 2017;7(9):e016591 Epub 2017/10/01. 10.1136/bmjopen-2017-016591 28963291 PMC5623486

[12] Bihorac A, Brennan M, Ozrazgat-Baslanti T, Bozorgmehri S, Efron PA, Moore FA, et al National surgical quality improvement program underestimates the risk associated with mild and moderate postoperative acute kidney injury. Crit Care Med. 2013;41(11):2570–83. Epub 2013/08/10. 10.1097/CCM.0b013e31829860fc 23928835 PMC3812338

[13] Birnie K, Verheyden V, Pagano D, Bhabra M, Tilling K, Sterne JA, et al Predictive models for kidney disease: improving global outcomes (KDIGO) defined acute kidney injury in UK cardiac surgery. Crit Care. 2014;18(6):606 Epub 2015/02/13. 10.1186/s13054-014-0606-x 25673427 PMC4258283

[14] Ng SY, Sanagou M, Wolfe R, Cochrane A, Smith JA, Reid CM. Prediction of acute kidney injury within 30 days of cardiac surgery. J Thorac Cardiovasc Surg. 2014;147(6):1875–83, 83 e1. Epub 2013/09/03. 10.1016/j.jtcvs.2013.06.049 . 23993316

[15] Flechet M, Guiza F, Schetz M, Wouters P, Vanhorebeek I, Derese I, et al AKIpredictor, an online prognostic calculator for acute kidney injury in adult critically ill patients: development, validation and comparison to serum neutrophil gelatinase-associated lipocalin. Intensive Care Med. 2017;43(6):764–73. Epub 2017/01/29. 10.1007/s00134-017-4678-3 . 28130688

[16] Haase M, Bellomo R, Story D, Letis A, Klemz K, Matalanis G, et al Effect of mean arterial pressure, haemoglobin and blood transfusion during cardiopulmonary bypass on post-operative acute kidney injury. Nephrol Dial Transplant. 2012;27(1):153–60. Epub 2011/06/17. 10.1093/ndt/gfr275 . 21677302

[17] Ozrazgat-Baslanti T, Blanc P, Thottakkara P, Ruppert M, Rashidi P, Momcilovic P, et al Preoperative assessment of the risk for multiple complications after surgery. Surgery. 2016;160(2):463–72. Epub 2016/05/31. 10.1016/j.surg.2016.04.013 27238354 PMC5114020

[18] Bihorac A, Ozrazgat-Baslanti T, Ebadi A, Motaei A, Madkour M, Pardalos PM, et al MySurgeryRisk: Development and Validation of a Machine-learning Risk Algorithm for Major Complications and Death After Surgery. Ann Surg. 2018 Epub 2018/03/01. 10.1097/SLA.0000000000002706 29489489 PMC6110979

[19] Collins GS, Reitsma JB, Altman DG, Moons KG. Transparent Reporting of a multivariable prediction model for Individual Prognosis or Diagnosis (TRIPOD): the TRIPOD statement. Annals of internal medicine. 2015;162(1):55–63. Epub 2015/01/07. 10.7326/M14-0697 . 25560714

[20] Mehta RL, McDonald B, Gabbai F, Pahl M, Farkas A, Pascual MT, et al Nephrology consultation in acute renal failure: does timing matter? Am J Med. 2002;113(6):456–61. Epub 2002/11/13. . 12427493 10.1016/s0002-9343(02)01230-5

[21] Kidney Disease: Improving Global Outcomes (KDIGO) Acute Kidney Injury Work Group—KDIGO Clinical Practice Guideline for Acute Kidney Injury. Kidney Int. 2012;suppl (2):1–138.

[22] Program UNAKI. Acute Kidney Injury (AKI) Algorithm. 2014.

[23] Holmes J, Roberts G, Meran S, Williams JD, Phillips AO, Welsh AKISG. Understanding Electronic AKI Alerts: Characterization by Definitional Rules. Kidney Int Rep. 2017;2(3):342–9. 10.1016/j.ekir.2016.12.001 29142963 PMC5678680

[24] Bellomo R, Ronco C, Kellum JA, Mehta RL, Palevsky P, Acute Dialysis Quality Initiative w. Acute renal failure—definition, outcome measures, animal models, fluid therapy and information technology needs: the Second International Consensus Conference of the Acute Dialysis Quality Initiative (ADQI) Group. Crit Care. 2004;8(4):R204–12. Epub 2004/08/18. 10.1186/cc2872 15312219 PMC522841

[25] Wald R, Waikar SS, Liangos O, Pereira BJ, Chertow GM, Jaber BL. Acute renal failure after endovascular vs open repair of abdominal aortic aneurysm. J Vasc Surg. 2006;43(3):460–6; discussion 6. Epub 2006/03/08. 10.1016/j.jvs.2005.11.053 . 16520155

[26] Wald R, Quinn RR, Luo J. Chronic dialysis and death among survivors of acute kidney injury requiring dialysis. JAMA. 2009;302:1179–85. 10.1001/jama.2009.1322 19755696

[27] Charlson ME, Pompei P, Ales KL, MacKenzie CR. A new method of classifying prognostic comorbidity in longitudinal studies: development and validation. Journal of Chronic Diseases. 1987;40(5):373–83. Epub 1987/01/01. . 3558716 10.1016/0021-9681(87)90171-8

[28] Korenkevych D, Ozrazgat-Baslanti T, Thottakkara P, Hobson CE, Pardalos P, Momcilovic P, et al The Pattern of Longitudinal Change in Serum Creatinine and 90-Day Mortality After Major Surgery. Ann Surg. 2016;263(6):1219–27. Epub 2015/07/17. 10.1097/SLA.0000000000001362 26181482 PMC4829495

[29] Levey AS, Stevens LA, Schmid CH, Zhang YL, Castro AF 3rd, Feldman HI et al A new equation to estimate glomerular filtration rate. Annals of internal medicine. 2009;150(9):604–12. Epub 2009/05/06. 150/9/604 [pii]. 19414839 10.7326/0003-4819-150-9-200905050-00006 PMC2763564

[30] Shickel B, Loftus TJ, Adhikari L, Ozrazgat-Baslanti T, Ebadi A, Bihorac A, et al DeepSOFA: A Real-Time Continuous Acuity Score Framework using Deep Learning. arXiv preprint arXiv:180210238. 2018.

[31] Thottakkara P, Ozrazgat-Baslanti T, Hupf BB, Rashidi P, Pardalos P, Momcilovic P, et al Application of Machine Learning Techniques to High-Dimensional Clinical Data to Forecast Postoperative Complications. PLoS One. 2016;11(5):e0155705 10.1371/journal.pone.0155705 27232332 PMC4883761

[32] Saria S, Rajani AK, Gould J, Koller D, Penn AA. Integration of early physiological responses predicts later illness severity in preterm infants. Sci Transl Med. 2010;2(48):48ra65 Epub 2010/09/10. 10.1126/scitranslmed.3001304 20826840 PMC3564961

[33] Wood SN. Thin plate regression splines. Journal of the Royal Statistical Society Series B-Statistical Methodology. 2003;65:95–114. 10.1111/1467-9868.00374 WOS:000180996800005.

[34] Breiman L. Random forest. Machine Learning. 2001;45(1):5–32. 10.1023/A:1010933404324 WOS:000170489900001.

[35] Pedregosa F, Varoquaux G, Gramfort A, Michel V, Thirion B, Grisel O, et al Scikit-learn: Machine Learning in Python. Journal of Machine Learning Research. 2011;12:2825–30. WOS:000298103200003.

[36] Angelo Canty BR. Boot: Functions and datasets for bootstrapping from the book “Bootstrap Methods and Their Application'' by A. C. Davison and D. V. Hinkley (1997, CUP), originally written by Angelo Canty for S. 2017.

[37] Youden WJ. Index for rating diagnostic tests. Cancer. 1950;3(1):32–5. Epub 1950/01/01. 10.1002/1097-0142(1950)3:1<32::Aid-Cncr2820030106>3.0.Co;2-3 . 15405679

[38] Pencina MJ, D'Agostino RB Sr., Steyerberg EW. Extensions of net reclassification improvement calculations to measure usefulness of new biomarkers. Stat Med. 2011;30(1):11–21. Epub 2011/01/05. 10.1002/sim.4085 21204120 PMC3341973

[39] Foundation PS. Python Language Reference, version 2.7. Available from: http://www.python.org.

[40] Jones E OE, Peterson P, et al SciPy: Open Source Scientific Tools for Python. 2001.

[41] team Rc. R: A language and environment for statistical computing. R Foundation for Statistical Computing 2017. Available from: http://www.R-project.org/.

[42] United States Census Bureau. American FactFinder 2010 [05/16/2017]. Available from: http://www2.census.gov/.

[43] Kane-Gill SL, Sileanu FE, Murugan R, Trietley GS, Handler SM, Kellum JA. Risk factors for acute kidney injury in older adults with critical illness: a retrospective cohort study. Am J Kidney Dis. 2015;65(6):860–9. Epub 2014/12/10. 10.1053/j.ajkd.2014.10.018 25488106 PMC4442750

[44] Coca SG, Garg AX, Swaminathan M, Garwood S, Hong K, Thiessen-Philbrook H, et al Preoperative angiotensin-converting enzyme inhibitors and angiotensin receptor blocker use and acute kidney injury in patients undergoing cardiac surgery. Nephrol Dial Transplant. 2013;28(11):2787–99. 10.1093/ndt/gft405 24081864 PMC3811062

[45] Roshanov PS, Rochwerg B, Patel A, Salehian O, Duceppe E, Belley-Côté EP, et al Withholding versus Continuing Angiotensin-converting Enzyme Inhibitors or Angiotensin II Receptor Blockers before Noncardiac SurgeryAn Analysis of the Vascular events In noncardiac Surgery patIents cOhort evaluatioN Prospective Cohort. Anesthesiology: The Journal of the American Society of Anesthesiologists. 2017;126(1):16–27. 10.1097/ALN.0000000000001404 27775997

[46] Roncero LMV, Poveda DS, García JJV, Barrado MES, Vecino JMC. Perioperative use of angiotensin-converting-enzyme inhibitors and angiotensin receptor antagonists. Journal of Clinical Anesthesia. 2017;40:91–8. 10.1016/j.jclinane.2017.04.018 28625460

[47] Li S, Krawczeski CD, Zappitelli M, Devarajan P, Thiessen-Philbrook H, Coca SG, et al Incidence, risk factors, and outcomes of acute kidney injury after pediatric cardiac surgery: A prospective multicenter study. Critical Care Medicine. 2011;39(6):1493–9. 10.1097/CCM.0b013e31821201d3 WOS:000290715000037. 21336114 PMC3286600

[48] Aronson S, Phillips-Bute B, Stafford-Smith M, Fontes M, Gaca J, Mathew JP, et al The association of postcardiac surgery acute kidney injury with intraoperative systolic blood pressure hypotension. Anesthesiol Res Pract. 2013;2013:174091 10.1155/2013/174091 24324489 PMC3845409

[49] Sun LY, Wijeysundera DN, Tait GA, Beattie WS. Association of intraoperative hypotension with acute kidney injury after elective noncardiac surgery. Anesthesiology. 2015;123(3):515–23. 10.1097/ALN.0000000000000765 . 26181335

[50] Bihorac A, Hobson CE. Acute kidney injury: Precision perioperative care protects the kidneys. Nat Rev Nephrol. 2017;14(1):8–10. Epub 2017/12/14. 10.1038/nrneph.2017.170 . 29234162 PMC6029882

[51] Futier E, Lefrant JY, Guinot PG, Godet T, Lorne E, Cuvillon P, et al Effect of Individualized vs Standard Blood Pressure Management Strategies on Postoperative Organ Dysfunction Among High-Risk Patients Undergoing Major Surgery: A Randomized Clinical Trial. JAMA. 2017;318(14):1346–57. Epub 2017/10/04. 10.1001/jama.2017.14172 28973220 PMC5710560

[52] Kertai MD, Zhou S, Karhausen JA, Cooter M, Jooste E, White WD, et al Platelet Counts, Acute Kidney Injury, and Mortality after Coronary Artery Bypass Grafting Surgery Reply. Anesthesiology. 2016;125(2):438–9. 10.1097/ALN.0000000000001190 WOS:000385022200043. 26599400 PMC5040517

[53] Oh HJ, Park JT, Kim JK, Yoo DE, Kim SJ, Han SH, et al Red blood cell distribution width is an independent predictor of mortality in acute kidney injury patients treated with continuous renal replacement therapy. Nephrology Dialysis Transplantation. 2012;27(2):589–94. 10.1093/ndt/gfr307 WOS:000300421300020. 21712489

[54] Borthwick E, Ferguson A. Perioperative acute kidney injury: risk factors, recognition, management, and outcomes. Bmj. 2010;341:c3365 10.1136/bmj.c3365 . 20603317

[55] Huen SC, Parikh CR. Predicting acute kidney injury after cardiac surgery: a systematic review. Ann Thorac Surg. 2012;93(1):337–47. S0003-4975(11)02155-2 [pii] 10.1016/j.athoracsur.2011.09.010 22186469 PMC3286599

[56] Hobson C, Ruchi R, Bihorac A. Perioperative Acute Kidney Injury: Risk Factors and Predictive Strategies. Crit Care Clin. 2017;33(2):379–96. 10.1016/j.ccc.2016.12.008 . 28284301 PMC5617733

[57] Kashani K, Steuernagle JHt, Akhoundi A, Alsara A, Hanson AC, Kor DJ. Vascular Surgery Kidney Injury Predictive Score: A Historical Cohort Study. J Cardiothorac Vasc Anesth. 2015;29(6):1588–95. Epub 2015/07/15. 10.1053/j.jvca.2015.04.013 . 26159745

