# Supplementary material for: Improved predictive models for acute kidney injury with IDEA: Intraoperative Data Embedded Analytics
Source: PLoS One. 2019 Apr 4;14(4):e0214904. doi: 10.1371/journal.pone.0214904 (PMC6448850; doi:10.1371/journal.pone.0214904)
Supplement: S1 Methods — (DOCX) [file pone.0214904.s001.docx]

**Supplementary Methods and Information**

This study was approved by the University of Florida Institutional Review Board and Privacy Office as an exempt study with a waiver of informed consent. We followed the recommendations for the Transparent Reporting of a multivariable prediction model for Individual Prognosis Or Diagnosis (TRIPOD) under the Type 2a analysis category (random split sample development and validation) (Table S1) [1].

***Data Source***

We used the University of Florida Health (UFH) Integrated Data Repository as an honest broker for data de-identification and created a single-center perioperative longitudinal cohort that integrated EHR with public datasets as previously described by Bihorac et al.[2] Using zip code of residency, we linked the cohort to United State Census data [3, 4] to calculate each patient’s residing neighborhood characteristics and distance from the hospital.[5] We included all inpatient operative procedures requiring at least a 24 hour hospital stay performed between January 1, 2000 and November 30, 2010. The date of death was determined using hospital records and searches of the Social Security Death Index and Florida Bureau of Vital Statistics in July 2014 to assess survival through January 31, 2014 using the full name, birth date, and social security number [6].

The dataset used for this analysis was a restricted dataset that included identifiers such as zip codes and dates which cannot be shared in its current form. However, it can be provided as a de-identified dataset from University of Florida Integrated Data Repository (contact is Gigi Lipori ([pflugg@shands.ufl.edu)](mailto:pflugg@shands.ufl.edu))) after IRB approval (University of Florida IRB contact is Peter Iafrate ([iafrate@ufl.edu](mailto:iafrate@ufl.edu))). The reference IRB number is IRB201703211.

***Participants***

We included all patients 18 years of age or older admitted to the hospital for longer than 24 hours following any type of inpatient operative procedure. If patients underwent multiple surgeries, only the first surgery was used in our analysis. We excluded patients with end stage renal disease prior to admission (n=1,935) and patients with missing serum creatinine (sCr) values during hospitalization (n=6,636). From the remaining cohort we included 2,911 patients who had complete intraoperative data for vital signs, laboratory values, and medications.

***Outcomes***

The main outcome of our model was the development of postoperative acute kidney injury (AKI) within the first seven days after surgery. The secondary analysis modeled a) the risk for the development of AKI within the first three days after surgery, and b) the risk of developing postoperative AKI at any point during the hospitalization for the index surgery. We defined AKI using the consensus Kidney Disease: Improving Global Outcomes (KDIGO) criteria as at least a 50% or 0.3 mg/dl increase in serum creatinine relative to the reference creatinine[7]. Reference creatinine was determined based on the availability of measured creatinine prior to admission. We used the minimum serum creatinine value if results were available within seven days of the index hospitalization. If not available, we used the median serum creatinine value obtained within 8-365 days prior to admission. For patients without a prior creatinine value within the year prior to admission and no history of chronic kidney disease, we estimated reference serum creatinine [8]. The estimated reference serum creatinine was calculated by solving the abbreviated “Modification of Diet in Renal Disease” equation for creatinine, assuming a glomerular filtration rate of 75 ml/minute/1.73 m^2^. After the first seven days of the index hospitalization, the minimum serum creatinine from the preceding seven days was used as the reference creatinine [2, 9]. Patients with chronic kidney disease and end stage renal disease prior to admission were identified using the validated combination of ICD-9-CM codes [10, 11].

***Predictor Variables***

We derived a set of 285 preoperative predictor features from 69 demographic, socio-economic, administrative, clinical, pharmacy, and laboratory variables. For each patient we considered all potential predictors. Patient comorbidities were derived using up to 50 preoperative International Classification of Diseases, Ninth Revision, Clinical Modification (ICD-9-CM) codes. We used validated methods to define binary comorbidity variables [12, 13] and the composite Charlson Comorbidity Index [14]. We extracted medications dispensed on the first admission day using RxNorms data grouped into drug classes according to the United States Department of Veterans Affairs National Drug File-Reference Terminology [15]. Five physiologic time series were used in the immediate postoperative risk assessment (mean arterial blood pressure (MAP), systolic blood pressure, diastolic blood pressure, minimum alveolar concentration (MAC), and heart rate (HR)). From these five time series, we extracted the following features:

- Base signal mean value
- Long-term variability (standard deviation of the base signal) [16]
- Short-term variability (standard deviation of the residual signal)
- Minimum value
- Maximum value
- Total duration of the time series
- Time spend (mins and as %) in the following ranges:
  - [mean – 2X standard deviation, mean - standard deviation]
  - [mean + standard deviation, mean + 2X standard deviation]
  - [mean - standard deviation, mean + standard deviation]
  - [mean - standard deviation, mean – 0.5X standard deviation]
  - [mean + 0.5X standard deviation, mean + standard deviation]
  - [mean - 0.5X standard deviation, mean + 0.5X standard deviation]
  - Less than (mean – 2X standard deviation)
  - Greater than (mean + 2X standard deviation)

We extracted the following intraoperative laboratory features from the 21 laboratory variables in S1 Table:

- Mean value
- Maximum value
- Minimum value
- Count
- Variance
- Abnormal value percentage (percentage of values outside the normal ranges from the Logical Observation Identifiers Names and Codes (LOINC) data table [https://loinc.org/downloads/])

We used binary variables to indicate if the patient took diuretic or pressor drugs during the surgery.

***Sample Size***

We included 2,911 patients in the cohort. The data was randomly split into 70% for training and 30% for validation. The algorithm was trained on the development cohort while results were reported from the validation cohort. By using 30% of the cohort for validation (n= 873), we estimated that the overall sample size allows for a maximum width of the 95% confidence interval for the area under the receiver operating characteristic curve (AUC) of 0.08, when prevalence of AKI is between 30% and 40%.

**Predictive Analytics Workflow**

The *IDEA* algorithm consists of two main layers, preoperative and intraoperative, (fig. 2) each containing two cores, *Data Transformer and Data Analytics*.

**Preoperative layer:**

This layer contains a real-time automated EHR algorithm that uses the intelligent perioperative platform developed by our group [17]. This platform resides in a secure environment, where in real-time, it integrates and transforms EHR data, runs predictive algorithms, produces outputs for physicians, collects their feedback, and prospectively collects data for the future retraining of prediction models (top layer of fig. 2). The preoperative layer consists of the *Data Transformer* and *Data Analytics* cores. The *Data Transformer core* integrates data from the various sources and then uses variable generation, data preprocessing, feature transformation, and feature selection to optimize the data for analysis. The *Data Analytics* core uses the generalized additive model (GAM) to compute risk probabilities for postoperative acute kidney injury for each patient.

*Data Transformer:* The *Data Transformer* core performs data preprocessing included data cleaning, outlier removal, and missing data imputation [18, 19]. We tagged the inconclusive test results as missing during the initial data cleaning. A set of automatic rules was used to remove outliers that were considered unreasonable observations by medical experts. The bottom and top 1% of values from the distributions of continuous variables were considered outliers and were imputed with a random number generated between the 0.5th and 5th percentiles or between 95th and 99.5th percentiles, respectively. All missing observations were imputed using an automated algorithm. We replaced missing nominal variables with a distinct ‘‘missing’’ category, whereas missing continuous variables were replaced by the median value for a given variable.

Feature transformation was applied to reduce the dimensionality of the data and decrease the chance of overfitting. We optimized categorical and nominal variables with multiple levels (such as surgeon’s ID or zip code) using conditional probabilities for a patient to have a particular variable value conditioning on each outcome [20]. We substituted the value of categorical variables with the ratio , where

and then treated each categorical variable as an ordered variable. Such substitutions, in case of classification trees, produce the optimal splits in terms of cross-entropy and Gini index. Based on our previous experiments, such modeling of categorical variables provides less overfitting than when using binary dummy variables.

In order to obtain a reliable estimate of , categorical risk factors within categories with fewer than 100 records were grouped together and labeled "other." This "other" group was further split into several subgroups where each subgroup contained categories with similar proportions of patients from different classes. This was achieved by performing k-means clustering [21] on the set of categories in the "other" group. We set the number of clusters to five. The computations were implemented in Python software using the k means function from the sc-kit learn package. The rationale for this approach was to reduce the over-fitting of the data, since large clusters are less affected by random data splits than small individual categories. Computational results showed that indeed this technique improved the stability of the model and reduced the effects of over-fitting.

Surgical procedure codes were optimized using a forest of trees approach to reduce the 4-digit primary procedure ICD-9-CM codes that correspond to the anatomical location of surgery. Although the full procedure codes may be important features for risk stratification, their high dimensionality renders them challenging to implement in predictive models. In addition, procedure codes that were only associated with a few patients in the cohort would create unreliable estimates of probabilities by counting the number of such patients in each class. The forest of trees optimization was done by creating trees, where each node, n, corresponds to a certain group of procedures and is described by a sequence of digits (Sn length varies from 2 to 4). Each successor of a given node has a code generated by adding one additional digit from the right to Sn. For each leaf node we assigned a number of patients who had a type of surgical procedure described by the node’s code, and for each non-leaf node (such nodes represent general classes of procedures) we assigned a number of patients whose type of surgical procedure belongs to this class. Procedures were aggregated up to the top level of the ICD-9-CM hierarchy (18 basic procedures classes) such that each procedure/group of procedures contained at least 100 patients. The value of this parameter was selected based on a grid search through the values 50, 100, 150, 250, and 500. We enumerated the obtained set of procedures or groups of procedures and the enumeration index was taken as a discrete feature in our model. The grouping method reduced the number of levels in procedures from 1,536 to 187 and improved the proportion of low frequency procedures.

Supervised feature selection was performed using the variance inflation factors to evaluate collinearity and remove highly collinear predictors (VIF package in R [22]). Due to the nature of clinical data, many predictors are derived from a single attribute thus the likelihood of collinearity among variables is high.

*Data Analytics:* A generalized additive model with logistic link function was trained to calculate patient-level risk probabilities for all three acute kidney injury outcomes [20, 23]. All models were adjusted for nonlinearity of all covariates using nonlinear risk functions estimated with cubic splines [20]. For each complication we used risk probabilities, calculated by the GAM algorithm, to define the optimal cutoff values that best categorized the patients into the high- and low-risk risk categories by maximizing the Youden Index [24].

The generalized additive model is explained in detail here. We estimated the probability of outcome (, otherwise ) by using a generalized additive model:

(1)

where is the number of risk factors, are the risk factors, are the values of these factors, is a nonlinear risk function associated with the th risk factor and is a free term. Nonlinear risk functions were estimated for each feature with cubic splines via a local scoring algorithm [25]. The degrees of freedom for each spline were estimated by maximizing restricted likelihood function [26]. Degrees of freedom characterize a curvature of a spline, with value 1 corresponding to a linear function. Risk predictors with estimated degrees of freedom close to 1 were not smoothed in the final model; instead the original values of risk predictors were used. Therefore, the final model has the following form as in equation (2) where is a set of risk predictors with estimated degrees of freedom close to 1 and is the linear weight of the risk predictors.

(2)

**Intraoperative layer:**

The intraoperative layer also consists of a *Data Transformer* and *Data Analytics* core. The *Data Transformer core* integrates data from various sources and then uses variable generation and data preprocessing for analysis. The *Data Analytics* layer trains a *random forest classifier* to compute risk probabilities for postoperative acute kidney injury for each individual patient.

*Data Transformer:* The postoperative *Data Transformer* is very similar to the preoperative *Data Transformer*, however, only intraoperative data is used for variable generation and data preprocessing. More importantly, the postoperative *Data Transformer* core produces two datasets: (1) the full perioperative dataset, which combines the preoperative and intraoperative features, and (2) the stacked dataset, which combines the preoperative prediction scores with the intraoperative features.

Common rules used for cleaning and preprocessing all vital signs:

1. Determine the time frame of the observations using the surgery date and time and considering only the first surgery for this analysis.

2. Order observations based on the time. If there are multiple observations at the same time point, then we computed the average value for that time point (NA values were removed when averaging the values).

3. Remove accounts with less than 30 observations from the data set.

**Cleaning blood pressure data:**

| **Variable Name** | **Range (Values Outside this are Considered an Outlier)** |
| --- | --- |
| Systolic Blood Pressure | (0,300) (mm Hg) |
| Diastolic Blood Pressure | (0,200) (mm Hg) |
| Mean Blood Pressure (MAP) | (0,200) (mm Hg) |

1. Mark observations outside of the ranges above as outliers and replace the corresponding values with NA.

2. Remove observations that are lacking all three blood pressure measurements.

3. Impute missing blood pressure values:

- If only one of the 3 blood pressure values are missing from an observation, then the missing value is calculated using the formula: MAP = 2/3*diastolic + 1/3*systolic.
- If two values are missing, then the missing values were estimated from the standard chart for blood pressure values.

4. Find isolated peaks and valleys and replace them using neighboring blood pressure values. We assumed the isolated peaks and valleys were due to mechanical malfunction as opposed to accurate readings. To identify the isolated peaks and valleys, a moving average filter was used to create a baseline time series. The absolute difference between the original and baseline time series was then calculated. A difference of 30 mmHg was used as the threshold to distinguish isolated peaks and valleys. The isolated peaks and valleys were then replaced by the corresponding value from the baseline time series.

5. Remove blood pressure data from the dataset for accounts containing less than 20 observations.

**Cleaning heart rate data:**

1. Replace extreme heart rate values with NA (allowable range: 1 to 219)

2. Trim observations that are part of a group of more than three consecutive NA heart rate observations. Substitute the remaining NA values with the average of their five nearest neighbors.

3. Find isolated peaks and valleys and replace them using neighboring heart rate values. We assumed the isolated peaks and valleys were due to mechanical malfunction as opposed to accurate readings. To identify the isolated peaks and valleys, a moving average filter was used to create a baseline time series. The absolute difference between the original and baseline time series was then calculated. A difference of 30 beats per minute was used as the threshold to distinguish isolated peaks and valleys. The isolated peaks and valleys were then replaced by the corresponding value from the baseline time series.

4. Remove heart rate data from the dataset for accounts containing less than 20 observations.

**Cleaning minimum alveolar concentration (MAC):**

1. Replace extreme MAC component values with NA (allowable range: 0 to 5 %)

2. Replace missing values with the average of the five closest observations.

3. Normalize the data using one of the following formulas:

MAC = expired isofluran/1.17 or MAC=expired sevoflurane/1.8

4. Find isolated peaks and valleys and replace them using neighboring MAC values. We assumed the isolated peaks and valleys were due to mechanical malfunction as opposed to accurate readings. To identify the isolated peaks and valleys, a moving average filter was used to create a baseline time series. The absolute difference between the original and baseline time series was then calculated. A difference of three percent was used as the threshold to distinguish isolated peaks and valleys. The isolated peaks and valleys were then replaced by the corresponding value from the baseline time series.

5. Remove MAC data from the dataset for accounts containing less than 15 observations.

*Data Analytics:* Random forest is a supervised machine learning algorithm that generates and utilizes a collection of numerous classification or regression trees and aggregates the results [27]. Each of the generated classifiers are trained on a bootstrap sample from the training data. Each tree generated by the random forest predicts an outcome, the model then generates the final outcome by aggregating the outcomes of all trees and selecting the most prevalent outcome. Random forest employs bootstrap aggregation [28] and random feature selection [29] to construct a set of decision trees, thus applying controlled variation [30]. We used the Python scikit-learn implementation of the random forest classifier [31]. The inter-tree variability of the forest was used to determine features importance.

**Validation**

All preoperative and postoperative models were validated using the validation data cohort of size 873 patients (that is 30% from the full data cohort). The results were reported using 2,000 nonparametric bootstrap replicates generated from R *boot* package [32]. The prediction results obtained from the 1,000 bootstrap cohorts were used to calculate nonparametric confidence intervals for each of the performance metrics.

**Model Performance**

We assessed each model’s discrimination using the AUC and model accuracy by determining the fraction of correct classifications for each model. Stratification into high- and low-risk groups was done by calculating the optimal cut-off point based on the maximum Youden Index [33] computed during the training process for each outcome. Using the optimal thresholds for risk probabilities, we built the classification table from which we calculated sensitivity, specificity, and positive and negative predictive values for each model. Model calibration was tested using the Hosmer-Lemeshow statistic [34]. Absolute risk was calculated as the percentage of cases for which acute kidney injury (AKI) occurred in high- and low-risk risk groups, respectively. Relative risk was calculated as the ratio of the absolute risk of AKI between high- and low-risk groups. The absolute risk was calculated for high- and low-risk groups for all three models and were compared using the R package “DTComPair” adjusting for multiple comparisons using the Bonferroni method. The Net Reclassification Index (NRI) [35] was used to quantify how well the postoperative model reclassifies AKI patients compared to the preoperative model. We used bootstrap sampling and nonparametric methods to obtain 95% confidence intervals for all performance measures. All analyses were performed using Python 2.7 [36], SciPy 1.0.0 [37], R 3.4 [38], and SAS 9.4 (Cary, NC) software.

**References**

1. Collins GS, Reitsma JB, Altman DG, Moons KG. Transparent Reporting of a multivariable prediction model for Individual Prognosis or Diagnosis (TRIPOD): the TRIPOD statement. Ann Intern Med. 2015;162(1):55-63. Epub 2015/01/07. doi: 10.7326/M14-0697. PubMed PMID: 25560714.

2. Bihorac A, Korenkevych D, Ozrazgat Baslanti T, Momcilovic P, Pardalos P, Segal MS, et al. Database communication enables machine learning classifiers to predict postoperative acute kidney injury. Critical care medicine. 2013;41(12):714.

3. United States Census Bureau. American FactFinder 2010 [05/16/2017]. Available from: <http://www2.census.gov/>.

4. DeNavas-Walt C, Proctor BD, Smith JC. Income, Poverty, and Health Insurance Coverage in the United States: 2009: U.S. Government Printing Office, Washington, DC.; 2010. 60-238 p.

5. Pebesma EJ, Bivand RS. Classes and methods for spatial data in R. R News [Internet]. 2005; 5(2). Available from: <http://cran.r-project.org/doc/Rnews/>. .

6. Bihorac A, Ozrazgat-Baslanti T, Mahanna E, Malik S, White P, Sorensen M, et al. Long-Term Outcomes for Different Forms of Stress Cardiomyopathy After Surgical Treatment for Subarachnoid Hemorrhage. Anesth Analg. 2016;122(5):1594-602. doi: 10.1213/ANE.0000000000001231. PubMed PMID: 27007075; PubMed Central PMCID: PMCPMC4857194.

7. Mehta RL, McDonald B, Gabbai F, Pahl M, Farkas A, Pascual MT, et al. Nephrology consultation in acute renal failure: does timing matter? Am J Med. 2002;113:456-61.

8. Holmes J, Roberts G, Meran S, Williams JD, Phillips AO, Welsh AKISG. Understanding Electronic AKI Alerts: Characterization by Definitional Rules. Kidney Int Rep. 2017;2(3):342-9. doi: 10.1016/j.ekir.2016.12.001. PubMed PMID: 29142963; PubMed Central PMCID: PMCPMC5678680.

9. Bellomo R, Kellum JA, Ronco C. Defining acute renal failure: physiological principles. Intensive Care Med. 2004;30:33-7.

10. Wald R, Waikar SS, Liangos O, Pereira BJ, Chertow GM, Jaber BL. Acute renal failure after endovascular vs open repair of abdominal aortic aneurysm. J Vasc Surg. 2006;43(3):460-6; discussion 6. Epub 2006/03/08. doi: 10.1016/j.jvs.2005.11.053. PubMed PMID: 16520155.

11. Wald R, Quinn RR, Luo J. Chronic dialysis and death among survivors of acute kidney injury requiring dialysis. Jama. 2009;302:1179-85.

12. Elixhauser A, Steiner C, Harris DR, Coffey RM. Comorbidity Measures for Use with Administrative Data. Medical Care. 1998;36(1):8-27.

13. Wald R, Deshpande R, Bell CM, Bargman JM. Survival to discharge among patients treated with continuous renal replacement therapy. Hemodialysis International. 2006;10(1):82-7.

14. Charlson ME, Pompei P, Ales KL, MacKenzie CR. A new method of classifying prognostic comorbidity in longitudinal studies: development and validation. J Chronic Dis. 1987;40(5):373-83. Epub 1987/01/01. PubMed PMID: 3558716.

15. U.S. Department of Veterans Affairs VHA. National Drug File – Reference Terminology (NDF-RT™) Documentation 2015 [05/16/2017]. Available from: <http://evs.nci.nih.gov/ftp1/NDF-RT/NDF-RT%20Documentation.pdf>

16. Saria S, Rajani AK, Gould J, Koller D, Penn AA. Integration of early physiological responses predicts later illness severity in preterm infants. Sci Transl Med. 2010;2(48):48ra65. Epub 2010/09/10. doi: 10.1126/scitranslmed.3001304. PubMed PMID: 20826840; PubMed Central PMCID: PMCPMC3564961.

17. Feng Z, Bhat RR, Yuan X, Freeman D, Baslanti T, Bihorac A, et al., editors. Intelligent Perioperative System: Towards Real-Time Big Data Analytics in Surgery Risk Assessment. 2017 IEEE 15th Intl Conf on Dependable, Autonomic and Secure Computing, 15th Intl Conf on Pervasive Intelligence and Computing, 3rd Intl Conf on Big Data Intelligence and Computing and Cyber Science and Technology Congress(DASC/PiCom/DataCom/CyberSciTech); 2017 6-10 Nov. 2017.

18. Boulle M. A grouping method for categorical attributes having very large number of values. Lect Notes Artif Int. 2005;3587:228-42. PubMed PMID: ISI:000230895100023.

19. Pyle D. Data preparation for data mining: Morgan Kaufmann; 1999.

20. Korenkevych D, Ozrazgat-Baslanti T, Thottakkara P, Hobson CE, Pardalos P, Momcilovic P, et al. The Pattern of Longitudinal Change in Serum Creatinine and 90-Day Mortality After Major Surgery. Annals of surgery. 2016;263(6):1219-27. Epub 2015/07/17. doi: 10.1097/SLA.0000000000001362. PubMed PMID: 26181482; PubMed Central PMCID: PMCPMC4829495.

21. Bishop CM. Pattern recognition and machine learning. New York: Springer; 2006. xx, 738 p. p.

22. Dongyu Lin DPF, and Lyle H. Ungar. VIF Regression: A Fast Regression Algorithm for Large Data. Journal of the American Statistical Association. 1.0 edMarch 2011.

23. Thottakkara P, Ozrazgat-Baslanti T, Hupf BB, Rashidi P, Pardalos P, Momcilovic P, et al. Application of Machine Learning Techniques to High-Dimensional Clinical Data to Forecast Postoperative Complications. PLoS One. 2016;11(5):e0155705. doi: 10.1371/journal.pone.0155705. PubMed PMID: 27232332; PubMed Central PMCID: PMCPMC4883761.

24. Fluss R, Faraggi D, Reiser B. Estimation of the Youden Index and its associated cutoff point. Biometrical Journal. 2005;47(4):458-72.

25. Hastie T, Tibshirani, R. Generalized Additive Models. 1st ed: Chapman and Hall; 1990.

26. Wood SN. Fast stable restricted maximum likelihood and marginal likelihood estimation of semiparametric generalized linear models. J Roy Stat Soc B. 2011;73:3-36. doi: DOI 10.1111/j.1467-9868.2010.00749.x. PubMed PMID: ISI:000285970300002.

27. Breiman L. Random forests. Mach Learn. 2001;45(1):5-32. doi: Doi 10.1023/A:1010933404324. PubMed PMID: WOS:000170489900001.

28. Breiman L. Bagging predictors. Mach Learn. 1996;24(2):123-40.

29. Ho TK. The random subspace method for constructing decision forests. IEEE transactions on pattern analysis and machine intelligence. 1998;20(8):832-44.

30. Gray KR, Aljabar P, Heckemann RA, Hammers A, Rueckert D, Initiative AsDN. Random forest-based similarity measures for multi-modal classification of Alzheimer's disease. NeuroImage. 2013;65:167-75.

31. Pedregosa F, Varoquaux G, Gramfort A, Michel V, Thirion B, Grisel O, et al. Scikit-learn: Machine Learning in Python. J Mach Learn Res. 2011;12:2825-30. PubMed PMID: WOS:000298103200003.

32. Angelo Canty BR. Boot: Functions and datasets for bootstrapping from the book ``Bootstrap Methods and Their Application'' by A. C. Davison and D. V. Hinkley (1997, CUP), originally written by Angelo Canty for S. 2017.

33. Youden WJ. Index for rating diagnostic tests. Cancer. 1950;3(1):32-5. Epub 1950/01/01. doi: Doi 10.1002/1097-0142(1950)3:1<32::Aid-Cncr2820030106>3.0.Co;2-3. PubMed PMID: 15405679.

34. Pepe MS, Feng Z, Huang Y, Longton G, Prentice R, Thompson IM, et al. Integrating the predictiveness of a marker with its performance as a classifier. Am J Epidemiol. 2008;167(3):362-8. doi: 10.1093/aje/kwm305. PubMed PMID: 17982157; PubMed Central PMCID: PMCPMC2939738.

35. Pencina MJ, D'Agostino RB, Sr., Steyerberg EW. Extensions of net reclassification improvement calculations to measure usefulness of new biomarkers. Stat Med. 2011;30(1):11-21. Epub 2011/01/05. doi: 10.1002/sim.4085. PubMed PMID: 21204120; PubMed Central PMCID: PMCPMC3341973.

36. Foundation PS. Python Language Reference, version 2.7. Available from: <http://www.python.org>.

37. Jones E OE, Peterson P, et al. SciPy: Open Source Scientific Tools for Python. 2001.

38. team Rc. R: A language and environment for statistical computing. R Foundation for Statistical Computing 2017. Available from: <http://www.R-project.org/>.
