# Supplementary material for: Improved predictive models for acute kidney injury with IDEA: Intraoperative Data Embedded Analytics
Source: PLoS One. 2019 Apr 4;14(4):e0214904. doi: 10.1371/journal.pone.0214904 (PMC6448850; doi:10.1371/journal.pone.0214904)
Supplement: S2 Table — (DOCX) [file pone.0214904.s003.docx]

**S2 Table. Characteristics of input variables.**

| **Variable** | **Type of Variable** | **Data Source** | **Number of categories** | **Type of Preprocessing** |
| --- | --- | --- | --- | --- |
| **Demographic variables** |  |  |  |  |
| Age (years) | Continuous | Derived |  | Imputation of outliers^a^; Nonlinear function^b^ |
| Gender | Binary | Raw | 2 |  |
| Race | Nominal | Raw | 5 | Optimization of categorical features^c^ |
| **Socioeconomic variables** |  |  |  |  |
| Primary Insurance | Nominal | Raw | 4 | Optimization of categorical features^c^ |
| Residency area characteristics |  |  |  |  |
| Zip code | Nominal | Raw | 10,000 | Transformation through link to Census data^d^ |
| County | Nominal | Raw | 71 | Optimization of categorical features^c^ |
| Rural area | Binary | Derived | 2 |  |
| Total Population | Continuous | Derived |  | Obtained using residency zip code with linkage to US Census data^d^; Imputation of outliers^a^ |
| Median Income | Continuous | Derived |  | Obtained using residency zip code with linkage to US Census data^d^; Imputation of outliers^a^ |
| Total Proportion of African- Americans | Continuous | Derived |  | Obtained using residency zip code with linkage to US Census data^d^; Imputation of outliers^a^ |
| Total Proportion of Hispanic | Continuous | Derived |  | Obtained using residency zip code with linkage to US Census data^d^; Imputation of outliers^a^ |
| Population Proportion Below Poverty | Continuous | Derived |  | Obtained using residency zip code with linkage to US Census data^d^; Imputation of outliers^a^ |
| Distance from Residency to Hospital (km) | Continuous | Derived |  | Calculated using residency zip code; Imputation of outliers^a^ |
| **Operative characteristics** |  |  |  |  |
| Day of admission | Nominal | Derived | 7 | Optimization of categorical features^c^ |
| Month of admission | Nominal | Derived | 12 | Optimization of categorical features^c^ |
| Year of admission | Nominal | Derived | 11 | Optimization of categorical features^c^ |
|  |  |  |  |  |
| Weekend admission | Binary | Derived | 2 |  |
| Attending Surgeon | Nominal | Raw | 520 | Optimization of categorical features^c^ |
| Admission Source | Nominal | Raw | 3 | Optimization of categorical features^c^ |
| Admission Type (Emergent/Elective) | Binary | Raw | 2 |  |
| Admitting type (Medicine/Surgery) | Binary | Derived | 2 |  |
| Admitting Service | Nominal | Derived | 46 | Optimization of categorical features^c^ |
| Surgery Type | Nominal | Derived | 5 | Optimization of categorical features^c^ |
| Time of surgery from admission (days) | Continuous | Derived |  | Imputation of outliers^a^; Nonlinear function^b^ |
| Diagnosis/Procedure |  |  |  |  |
| Primary surgical procedure | Nominal | Derived | 1555 | Forest tree analysis of ICD9 codes^e^ |
| Major Diagnosis Category | Nominal | Raw | 28 | Optimization of categorical features^c^ |
| **Comorbidities** |  |  |  |  |
| Charlson's comorbidity index | Nominal | Derived | 18 | Optimization of categorical features^c^ |
| Number of diagnosis | Continuous | Derived |  | Imputation of outliers^a^; Nonlinear function^b^ |
| Myocardial Infarction | Binary | Derived | 2 |  |
| Congestive Heart Failure | Binary | Derived | 2 |  |
| Peripheral Vascular Disease | Binary | Derived | 2 |  |
| Cerebrovascular Disease | Binary | Derived | 2 |  |
| Chronic Pulmonary Disease | Binary | Derived | 2 |  |
| Diabetes | Binary | Derived | 2 |  |
| Cancer | Binary | Derived | 2 |  |
| Liver Disease | Binary | Derived | 2 |  |
| Valvular disease | Binary | Derived | 2 |  |
| Hypothyroidism | Binary | Derived | 2 |  |
| Coagulopthy | Binary | Derived | 2 |  |
| Obesity | Binary | Derived | 2 |  |
| Weight loss | Binary | Derived | 2 |  |
| Fluid and electrolyte disorders | Binary | Derived | 2 |  |
| Chronic anemia | Binary | Derived | 2 |  |
| Alcohol or drug abuse | Binary | Derived | 2 |  |
| Depression | Binary | Derived | 2 |  |
| Hypertension | Binary | Derived | 2 |  |
| Chronic kidney disease | Binary | Derived | 2 |  |
| End Stage Renal disease | Binary | Derived | 2 |  |
| **Admission day Medications^f^** |  |  |  |  |
| Number of Medications on Admission | Continuous | Derived |  | Imputation of outliers^a^; Nonlinear function^b^ |
| Betablockers | Binary | Derived | 2 |  |
| Diuretics | Binary | Derived | 2 |  |
| Statin | Binary | Derived | 2 |  |
| Aspirin | Binary | Derived | 2 |  |
| Angiotensin-Converting-Enzyme Inhibitors | Binary | Derived | 2 |  |
| Vasopressors or inotropes | Binary | Derived | 2 |  |
| Bicarbonate | Binary | Derived | 2 |  |
| Antiemetic | Binary | Derived | 2 |  |
| Aminoglycosides | Binary | Derived | 2 |  |
| Steroids | Binary | Derived | 2 |  |
| Vancomycin | Binary | Derived | 2 |  |
| Nonsteroidal anti-inflammatory drug | Binary | Derived | 2 |  |
| Number of Nephrotoxic Medications | Continuous | Derived |  | Imputation of outliers^a^; Nonlinear function^b^ |
| **Preoperative laboratory results** |  |  |  |  |
| Reference estimated glomerular filtration rate | Continuous | Derived |  | Imputation of outliers^a^ ; Nonlinear function^b^ |
| Ratio of reference creatinine to MDRD creatinine | Continuous | Derived |  | Imputation of outliers^a^ |
| Hemoglobin, g/dl | Continuous | Raw |  | Imputation of outliers^a^ ; Nonlinear function^b^ |
| Automated urinalysis, urine protein, mg/dL | Nominal | Derived | 3 | Optimization of categorical features^c^ |
| Automated urinalysis, urine hemoglobin, mg/dL | Nominal | Derived | 3 | Optimization of categorical features^c^ |
| Automated urinalysis, urine glucose, mg/dL | Nominal | Derived | 3 | Optimization of categorical features^c^ |
| Number of complete blood count tests | Nominal | Derived | 3 | Optimization of categorical features^c^ |
| **Physiologic intraoperative time series** |  |  |  |  |
| Mean arterial blood pressure (Invasive), mmHg | Continuous | Raw |  | Data cleaning^g^; Imputation of outliers^h^; Statistical features extraction^i^ |
| Systolic blood pressure (Invasive), mmHg | Continuous | Raw |  | Data cleaning^g^; Imputation of outliers^h^; Statistical features extraction^i^ |
| Diastolic blood pressure (Invasive), mmHg | Continuous | Raw |  | Data cleaning^g^; Imputation of outliers^h^; Statistical features extraction^i^ |
| Minimum alveolar concentration | Continuous | Raw |  | Data cleaning^g^; Imputation of outliers^h^; Statistical features extraction^i^ |
| Heart rate, bpm | Continuous | Raw |  | Data cleaning^g^; Imputation of outliers^h^; Statistical features extraction^i^ |
| Peripheral capillary oxygen saturation (SPO2) | Continuous | Raw |  | Data cleaning^g^; Imputation of outliers^h^ |
|  |  |  |  |  |
| **Laboratory results on surgery** |  |  |  |  |
| pH | Continuous | Raw |  | Missing data imputation^j^; Statistical features extraction^k^; Outlier removal^a^ |
| Lactic acid, mmol/L | Continuous | Raw |  | Missing data imputation^j^; Statistical features extraction^k^; Outlier removal^a^ |
| Hemoglobin, g/dL | Continuous | Raw |  | Missing data imputation^j^; Statistical features extraction^k^; Outlier removal^a^ |
| Hematocrit, vol% | Continuous | Raw |  | Missing data imputation^j^; Statistical features extraction^k^; Outlier removal^a^ |
| Partial pressure of oxygen (PO2)- Arterial, mmHg | Continuous | Raw |  | Missing data imputation^j^; Statistical features extraction^k^; Outlier removal^a^ |
| Fraction of inspired oxygen (FIO2) | Continuous | Raw |  | Missing data imputation^j^; Statistical features extraction^k^; Outlier removal^a^ |
| Partial pressure of oxygen and Fraction of inspired oxygen (PF) ratio^l^ | Continuous | Derived |  | Missing data imputation^l^; Outlier removal^a^ |
| Mean corpuscular hemoglobin, pg/[red cell] | Continuous | Raw |  | Missing data imputation^j^; Statistical features extraction^k^; Outlier removal^a^ |
| Mean corpuscular hemoglobin concentration, g/dL | Continuous | Raw |  | Missing data imputation^j^; Statistical features extraction^k^; Outlier removal^a^ |
| Mean corpuscular volume, fl/[red cell]L | Continuous | Raw |  | Missing data imputation^j^; Statistical features extraction^k^; Outlier removal^a^ |
| Red Blood Cells, million cells/mcL | Continuous | Raw |  | Missing data imputation^j^; Statistical features extraction^k^; Outlier removal^a^ |
| Red Cell Distribution Width, % | Continuous | Raw |  | Missing data imputation^j^; Statistical features extraction^k^; Outlier removal^a^ |
| White Blood Cells, 10*9/L | Continuous | Raw |  | Missing data imputation^j^; Statistical features extraction^k^; Outlier removal^a^ |
| Mean Platelet Volume, fL | Continuous | Raw |  | Missing data imputation^j^; Statistical features extraction^k^; Outlier removal^a^ |
| Platelets Count, 10*9/L | Continuous | Raw |  | Missing data imputation^j^; Statistical features extraction^k^; Outlier removal^a^ |
| Carboxyhemoglobin-Arterial, % | Continuous | Raw |  | Missing data imputation^j^; Statistical features extraction^k^; Outlier removal^a^ |
| Oxygen Content-Arterial, % | Continuous | Raw |  | Missing data imputation^j^; Statistical features extraction^k^; Outlier removal^a^ |
| Oxygen saturation, % | Continuous | Raw |  | Missing data imputation^j^; Statistical features extraction^k^; Outlier removal^a^ |
| Bicarbonate-Arterial, mmol/L | Continuous | Raw |  | Missing data imputation^j^; Statistical features extraction^k^; Outlier removal^a^ |
| Methemoglobin, % | Continuous | Raw |  | Missing data imputation^j^; Statistical features extraction^k^; Outlier removal^a^ |
| Partial pressure of carbon dioxide, mmHg | Continuous | Raw |  | Missing data imputation^j^; Statistical features extraction^k^; Outlier removal^a^ |
|  |  |  |  |  |
| **Medications during surgery** |  |  |  |  |
| Vasopressors | Binary | Derived | 2 |  |
| Diuretics | Binary | Derived | 2 |  |
| **Other operative characteristics** |  |  |  |  |
| Anesthesia type | Nominal | Raw | 2 |  |
| Duration of surgery, min | Continuous | Derived |  | Outlier removal^a^ |
| Estimated blood loss, mL | Continuous | Raw |  | Outlier removal^a^ |
| Urine output, mL | Continuous | Raw |  | Outlier removal^a^ |
| Intravenous Fluid, mL | Continuous | Raw |  | Outlier removal^a^ |
| Blood product, mL | Continuous | Raw |  | Outlier removal^a^ |

Abbreviation: ICD9, International Classification of Diseases, Ninth Revision; MDRD, Modification of Diet in Renal Disease.

MDRD creatinine was calculated by solving the abbreviated “Modification of diet in renal disease” equation for creatinine assuming a glomerular filtration rate of 75 ml/minute/1.73 m^2^ with formula (186/GFR) * (0.742*[if female])*(1.21*[if black])* age^(-0.203)^] ^(1/1.154)^ .[1]

Reference estimated glomerular filtration rate (eGFR) using standardized reference serum creatinine, sex, race, and age. [2]

Different set of variables were kept in final models (preoperative or intraoperative) from the input set provided in the table.

^a^ For continuous variables, observations that fell in the top and bottom 1% of the distribution were considered as outliers and imputed by neighborhood values (i.e., above 99% are imputed randomly from a uniform distribution defined over [95%, 99.5%] percentiles and below 1% are imputed randomly from another uniform distribution defined over [0.5%, 5%] percentiles. [3, 4]

^b^ Nonlinear risk function was calculated for continuous functions entered to the preoperative models.[4]

^c^ For categorical variables with more than two levels, levels were transformed to a numeric value as detailed in Methods section.

^d^ Using residency zip code, we linked to US Census data to calculate residing neighborhood characteristics and distance from hospital.

^e^ Surgical procedure codes were optimized using forest tree analysis of ICD-9-CM codes as detailed in Methods section.

^f^ Medications were dispensed on the first admission day using RxNorms data grouped into drug classes according to the US, Department of Veterans Affairs National Drug File-Reference Terminology.

^g^ We used observations for the first surgery, in case multiple surgeries exist. We averaged values if multiple observations exist at a time point. Only accounts with more than 30 observations were considered.

^h^ Values out of the predefined ranges were removed. Additionally, values in the top and bottom 0.5% of each of the time series distributions were removed.

^i^ We extracted several descriptive statistical measures including mean and standard deviation of base signal and standard deviation of residual signal of time series, minimum and maximum values observed, time/percentage of time a patient spent in a specific range of values for each of the time series. [5]

^j^ Observations during the first surgery have been considered. Missing values were imputed using surgery day observations for the given account.

^k^ We extracted descriptive statistical features including minimum, mean, maximum, count, variance, and abnormal observation percentage (calculated as = abnormal value count/ total observations * 100).

^l^ PF-ratio (ratio of arterial oxygen partial pressure to fractional inspired oxygen) was calculated using PO2/FIO2. Missing PF-ratios are replaced by ((SF-ratio) – 64)/0.84, where SF-ratio = SPO2/FIO2.[6]

References:

1. Bellomo R, Ronco C, Kellum JA, Mehta RL, Palevsky P. Acute renal failure - definition, outcome measures, animal models, fluid therapy and information technology needs: the Second International Consensus Conference of the Acute Dialysis Quality Initiative (ADQI) Group. Crit Care. 2004;8(4):R204-12. Epub 2004 May 24.

2. Levey AS, Stevens LA, Schmid CH, Zhang YL, Castro AF, 3rd, Feldman HI, et al. A new equation to estimate glomerular filtration rate. Annals of internal medicine. 2009;150(9):604-12. Epub 2009/05/06. doi: 150/9/604 [pii]. PubMed PMID: 19414839; PubMed Central PMCID: PMC2763564.

3. Thottakkara P, Ozrazgat-Baslanti T, Hupf BB, Rashidi P, Pardalos P, Momcilovic P, et al. Application of Machine Learning Techniques to High-Dimensional Clinical Data to Forecast Postoperative Complications. PLoS One. 2016;11(5):e0155705. doi: 10.1371/journal.pone.0155705. PubMed PMID: 27232332; PubMed Central PMCID: PMCPMC4883761.

4. Bihorac A, Ozrazgat-Baslanti T, Ebadi A, Motaei A, Madkour M, Pardalos PM, et al. MySurgeryRisk: Development and Validation of a Machine-learning Risk Algorithm for Major Complications and Death After Surgery. Ann Surg. 2018. Epub 2018/03/01. doi: 10.1097/SLA.0000000000002706. PubMed PMID: 29489489; PubMed Central PMCID: PMCPMC6110979.

5. Saria S, Rajani AK, Gould J, Koller D, Penn AA. Integration of early physiological responses predicts later illness severity in preterm infants. Sci Transl Med. 2010;2(48):48ra65. Epub 2010/09/10. doi: 10.1126/scitranslmed.3001304. PubMed PMID: 20826840; PubMed Central PMCID: PMCPMC3564961.

6. Rice TW, Wheeler AP, Bernard GR, Hayden DL, Schoenfeld DA, Ware LB. Comparison of the SpO2/FIO2 ratio and the PaO2/FIO2 ratio in patients with acute lung injury or ARDS. Chest. 2007;132(2):410-7. Epub 2007/06/19. doi: 10.1378/chest.07-0617. PubMed PMID: 17573487.
