# Supplementary material for: Improved predictive models for acute kidney injury with IDEA: Intraoperative Data Embedded Analytics
Source: PLoS One. 2019 Apr 4;14(4):e0214904. doi: 10.1371/journal.pone.0214904 (PMC6448850; doi:10.1371/journal.pone.0214904)
Supplement: S3 Table — (DOCX) [file pone.0214904.s004.docx]

**S3 Table. Preoperative and intraoperative clinical characteristics and outcomes of patients stratified by the occurrence of acute kidney injury.**

|  |  | **Acute Kidney Injury with onset in the first three postoperative days** | | **Acute Kidney Injury with onset in the first seven postoperative days** | | | **Acute Kidney Injury at any time** | | | |  |
| --- | --- | --- | --- | --- | --- | --- | --- | --- | --- | --- | --- |
|  |  | **No** | **Yes** | **No** | | **Yes** | **No** | | **Yes** | |  |
|  | **Overall cohort (N=2,911)** | **(N=1,913, 66%)** | **(N=998, 34%)** | **(N=1,748, 60%))** | | **(N=1,163, 40%)** | **(N=1,572, 54%)** | | **(N=1,339, 46%)** | |  |
| **Demographic features** |  |  |  |  | |  |  | |  | |  |
| Age, median (25th-75th) | 60 (49, 69) | 58 (47, 68) | 63 (52, 72)^a^ | 58 (47, 67) | | 63 (52, 72)^a^ | 58 (46, 67) | | 63 (52, 71)^a^ | |  |
| Male gender, n (%) | 1760 (60) | 1101 (58) | 659 (66)^a^ | 1007 (58) | | 753 (65) ^a^ | 898 (57) | | 862 (64)^a^ | |  |
| Race, n (%) |  |  |  |  | |  |  | |  | |  |
| White | 2374 (82) | 1579 (83 ) | 795 (80) | 1440 (82) | | 934 (80) | 1295 (82) | | 1079 (81) | |  |
| African American | 265 (9) | 167 (9) | 98 (10) | 150 (9) | | 115 (10) | 133 (8) | | 132 (10) | |  |
| Hispanic | 117 (4) | 66 (3) | 51 (5) | 64 (4) | | 53 (5) | 57 (4) | | 60 (4.48) | |  |
| Missing | 87 (3) | 52 (3) | 35 (4) | 50 (3) | | 37 (3) | 44 (3) | | 43 (3.2) | |  |
| Other | 68 (2) | 49 (3) | 19 (2) | 44 (3) | | 24 (2) | 43 (3) | | 25 (2) | |  |
| Primary insurance, n (%)^a^ |  |  |  |  | |  |  | |  | |  |
| Private | 1208 (42) | 844 (44) | 364 (36) | 797 (46) | | 411 (35) | 719 (46) | | 489 (37) | |  |
| Medicare | 1204 (41) | 726 (38) | 478 (48) | 635 (36) | | 569 (49) | 565 (36) | | 639 (48) | |  |
| Medicaid | 340 (12) | 223 (12) | 117 (12) | 198 (11) | | 142 (12) | 177 (11) | | 163 (12.17) | |  |
| Uninsured | 159 (5) | 120 (6) | 39 (4) | 118 (7) | | 41 (4) | 111 (7) | | 48 (4) | |  |
| **Socio-economic features** |  |  |  |  | |  |  | |  | |  |
| Neighborhood characteristics |  |  |  |  | |  |  | |  | |  |
| Rural area, n (%) | 767 (26) | 503 (26) | 264 (27) | 467 (27) | | 300 (26) | 431 (27) | | 336 (25.1) | |  |
| Total population, median (25th-75th) | 19162 (10639, 30611) | 18931 (10510, 30611) | 19363 (11056, 30570) | 18931 (10510, 30611) | | 19287 (11056, 30533) | 18931 (10448, 30459) | | 19363 (11116, 30649) | |  |
| Median income, median (25th-75th) | 34372 (29980, 41410) | 34289 (29854, 41410) | 34476 (30166, 41191) | 34328 (29854, 41410) | | 34459 (30084, 41410) | 34285 (29689, 41363) | | 34476 (30284, 41410) | |  |
| Total proportion of African-Americans (%), median (25th-75th) | 9.6 (3.9, 17.6) | 9.5 (4.0, 16.8) | 9.6 (3.8, 18.9) | 9.5 (3.9, 16.5) | | 9.6 (3.7, 19.5) | 9.5 (3.9, 16.6) | | 9.6 (3.9, 19.5) | |  |
| Total proportion of Hispanic (%),median (25th-75th) | 4.3 (2.5, 6.8) | 4.3 (2.6, 6.7) | 4.1 (2.5, 7.4) | 4.3 (2.6, 6.7) | | 4.1 (2.5, 7.1) | 4.3 (2.7, 6.7) | | 4.2 (2.5, 7.2) | |  |
| Distance from residency to hospital (km),  median (25th-75th) | 68 (29, 143) | 62 (28, 131) | 74 (31, 156)^a^ | 61 (28, 132) | | 73 (31, 153)^a^ | 58 (28, 130) | | 72 (31, 153)^a^ | |  |
| Population proportion below poverty (%), median (25th-75th) | 12.0 (8.2, 17.4) | 12.0 (8.2, 17.4) | 11.8 (8.0, 17.2) | 12.1 (8.3, 17.2) | | 11.8 (8.0, 17.4) | 12.1 (8.3, 17.4) | | 11.8 (8.0, 17.3) | |  |
| County (top 3 categories), n (%) |  |  |  |  | |  |  | |  | |  |
| Alachua | 383 (13) | 261 (14) | 122 (12)^a^ | 244 (14) | | 139 (12) | 221 (14) | | 162 (12) | |  |
| Marion | 250 (9) | 190 (10) | 60 (6)^a^ | 177 (10) | | 73 (6) | 162 (10) | | 88 (7) | |  |
| Georgia | 142 (5) | 88 (5) | 54 (5)^a^ | 79 (5) | | 63 (5) | 68 (4) | | 74 (5.5) | |  |
| **Comorbidity features** |  |  |  |  | |  |  | |  | |  |
| Charlson's comorbidity index (CCI), median (25th-75th) | 2 (1, 3) | 1 (0, 3) | 2 (1, 3)^a^ | 1 (0, 3) | | 2 (1, 3)^a^ | 1 (0, 3) | | 2 (1, 3)^a^ | |  |
| Chronic kidney disease, n (%) | 346 (12) | 94 (5) | 252 (25)^a^ | 73 (4) | | 273 (23)^a^ | 52 (3) | | 294 (22)^a^ | |  |
| Cancer, n (%) | 418 (14) | 323 (17) | 95 (10)^a^ | 303 (17) | | 115 (10)^a^ | 269 (17) | | 149 (11.13)^a^ | |  |
| Diabetes, n (%) | 539 (19) | 330 (17) | 209 (21)^a^ | 297 (17) | | 242 (21)^a^ | 267 (17) | | 272 (20.31)^a^ | |  |
| Chronic pulmonary disease, n (%) | 656 (23) | 377 (20) | 279 (28)^a^ | 331 (19) | | 325 (28)^a^ | 291 (19) | | 365 (27.26)^a^ | |  |
| Peripheral vascular disease, n (%) | 692 (24) | 386 (20) | 306 (31)^a^ | 334 (19) | | 358 (31)^a^ | 302 (19) | | 390 (29.13)^a^ | |  |
| Cerebrovascular disease, n (%) | 248 (9) | 170 (9) | 78 (8) | 154 (9) | | 94 (8) | 129 (8) | | 119 (8.89) | |  |
| Congestive heart failure, n (%) | 510 (18) | 228 (12) | 282 (28)^a^ | 180 (10) | | 330 (28)^a^ | 151 (10) | | 359 (26.81)^a^ | |  |
| Myocardial infarction, n (%) | 308 (11) | 158 (8) | 150 (15)^a^ | 140 (8) | | 168 (14)^a^ | 118 (8) | | 190 (14.19)^a^ | |  |
| Liver disease, n (%) | 393 (14) | 198 (10) | 195 (20)^a^ | 170 (10) | | 223 (19)^a^ | 135 (9) | | 258 (19.27)^a^ | |  |
| ***Preoperative characteristics of patients stratified by acute kidney injury*** | | | |  |  | |  |  | | |  |
| **Operative features** |  |  |  |  | |  |  | |  | |  |
| **Admission** |  |  |  |  | |  |  | |  | |  |
| Weekend admission, n (%) | 472 (16) | 281 (15) | 191 (19)^a^ | 255 (15) | | 217 (19)^a^ | 230 (15) | | 242 (18)^a^ | |  |
| Admission source, n (%)^a^ |  |  |  |  | |  |  | |  | |  |
| Outpatient setting | 1753 (61) | 1202 (63) | 551 (55) | 1105 (64) | | 648 (56) | 1017 (65) | | 736 (55) | |  |
| Emergency room | 583 (20) | 384 (20) | 199 (20) | 362 (21) | | 221 (19) | 325 (21) | | 258 (19.3) | |  |
| Transfer | 560 (19) | 313 (16) | 247 (25) | 268 (15) | | 292 (25) | 219 (14) | | 341 (26) | |  |
| Admission month (top 3 categories), n (%) |  |  |  |  | |  |  | |  | |  |
| September | 279 (10) | 184 (10) | 95 (10) | 168 (10) | | 111 (10) | 149 (10) | | 130 (9.7) | |  |
| October | 273 (9) | 195 (10) | 78 (8) | 174 (10) | | 99 (9) | 160 (10) | | 113 (8) | |  |
| June | 254 (9) | 178 (9) | 76 (8) | 162 (9) | | 92 (8) | 148 (9) | | 106 (8) | |  |
| Number of operating surgeons, n | 129 | 114 | 75 | 112 | | 82 | 106 | | 91 | |  |
| Number of procedures per operating Surgeon, n (%)^a^ |  |  |  |  | |  |  | |  | |  |
| First rank | 422 (15) | 211 (11) | 211 (21) | 181 (10) | | 241 (21) | 162 (10) | | 260 (19) | |  |
| Second rank | 267 (9) | 207 (11) | 60 (6) | 197 (11) | | 70 (6) | 178 (11) | | 89 (7) | |  |
| Third rank | 258 (9) | 144 (8) | 114 (11) | 129 (7) | | 129 (11) | 113 (7) | | 145 (11) | |  |
| Admitting type, n (%)^a^ |  |  |  |  | |  |  | |  | |  |
| Surgery | 2545 (87) | 1725 (90) | 820 (82) | 1588 (91) | | 957 (82) | 1437 (91) | | 1108 (83) | |  |
| Medicine | 366 (13) | 188 (10) | 178 (18) | 160 (9) | | 206 (18) | 135 (9) | | 231 (17) | |  |
| Emergent surgery, n (%) | 1352 (46) | 825 (43) | 527 (53)^a^ | 748 (43) | | 604 (52)^a^ | 644 (41) | | 708 (53)^a^ | |  |
| Surgery type, n (%)^a^ |  |  |  |  | |  |  | |  | |  |
| Cardiothoracic Surgery | 1415 (49) | 782 (41) | 633 (63) | 676 (39) | | 739 (64) | 596 (38) | | 819 (61) | |  |
| Non-Cardiac General Surgery | 952 (33) | 659 (34) | 293 (29) | 614 (35) | | 338 (29) | 552 (35) | | 400 (30) | |  |
| Neurologic Surgery | 301 (10) | 279 (15) | 22 (2) | 271 (16) | | 30 (3) | 243 (15) | | 58 (4) | |  |
| Specialty Surgeries ^b^ | 243 (8) | 193 (10) | 50 (5) | 187 (11) | | 56 (5) | 181 (11) | | 62 (5) | |  |
| Time between admission and operation (days) | 0 (0, 2) | 0 (0, 1) | 1 (0, 4)^a^ | 0 (0, 1) | | 1 (0, 4)^a^ | 0 (0, 1) | | 1 (0, 4)^a^ | |  |
| **Admission day medications** |  |  |  |  | |  |  | |  | |  |
| Diuretics | 600 (21) | 323 (17) | 277 (28)^a^ | 285 (16) | | 315 (27)^a^ | 252 (16) | | 348 (26)^a^ | |  |
| Bicarbonate | 295 (10) | 162 (8) | 133 (13)^a^ | 144 (8) | | 151 (13)^a^ | 126 (8) | | 169 (13)^a^ | |  |
| Angiotensin-Converting-Enzyme Inhibitors | 351 (12) | 205 (11) | 146 (15)^a^ | 182 (10) | | 169 (15)^a^ | 163 (10) | | 188 (14)^a^ | |  |
| Antiemetic | 1413 (49) | 1008 (53) | 405 (41)^a^ | 946 (54) | | 467 (40)^a^ | 861 (55) | | 552 (41)^a^ | |  |
| Betablockers | 872 (30) | 561 (29) | 311 (31) | 513 (29) | | 359 (31) | 460 (29) | | 412 (31) | |  |
| Statin | 502 (17) | 298 (16) | 204 (20)^a^ | 272 (16) | | 230 (20)^a^ | 254 (16) | | 248 (19) | |  |
| Pressors or inotropes | 424 (15) | 228 (12) | 196 (20)^a^ | 201 (12) | | 223 (20)^a^ | 173 (11) | | 251 (19)^a^ | |  |
| ***Intraoperative characteristics of patients stratified by acute kidney injury*** | | | |  |  | |  |  | | |  |
| **Physiologic intraoperative time series variables, mean (SD)** |  |  |  |  | |  |  | |  |  |  |
| **Systolic Blood Pressure (mm Hg)** |  |  |  |  | |  |  | |  |  |  |
| Maximum | 225.31 (33.62) | 224.4 (34.39) | 227.04 (32.04)^a^ | 223.96 (34.75) | | 227.34 (31.76)^a^ | 223.51 (34.87) | | 227.42 (31.97)^a^ |  |  |
| Minimum | 41.16 (21.68) | 42.77 (23.02) | 38.08 (18.46)^a^ | 43.83 (23.09) | | 37.15 (18.68)^a^ | 43.78 (23.21) | | 38.09 (19.3)^a^ |  |  |
| Average of base signal | 106.77 (16.69) | 109.16 (16.68) | 102.2 (15.73)^a^ | 109.92 (16.63) | | 102.03 (15.64)^a^ | 110.18 (16.66) | | 102.76 (15.82)^a^ |  |  |
| Long-term variability | 20.27 (6.61) | 19.68 (6.57) | 21.4 (6.55)^a^ | 19.47 (6.51) | | 21.48 (6.59)^a^ | 19.52 (6.61) | | 21.15 (6.51)^a^ |  |  |
| Short-term variability | 7.86 (2.63) | 7.77 (2.68) | 8.05 (2.53)^a^ | 7.72 (2.69) | | 8.07 (2.53)^a^ | 7.72 (2.73) | | 8.03 (2.51)^a^ |  |  |
| **Diastolic Blood Pressure (mm Hg)** |  |  |  |  | |  |  | |  |  |  |
| Maximum | 133.86 (24.43) | 133.02 (24.52) | 135.47 (24.2)^a^ | 132.55 (24.49) | | 135.83 (24.22)^a^ | 132.4 (24.61) | | 135.58 (24.12)^a^ |  |  |
| Minimum | 19.89 (14.61) | 20.66 (15.32) | 18.43 (13.04)^a^ | 21.13 (15.45) | | 18.03 (13.05)^a^ | 21.01 (15.62) | | 18.58 (13.22)^a^ |  |  |
| Average of base signal | 59.83 (8.84) | 61.08 (8.94) | 57.42 (8.13)^a^ | 61.49 (8.98) | | 57.32 (8.0)^a^ | 61.63 (9.06) | | 57.71 (8.08)^a^ |  |  |
| Long-term variability | 11.02 (3.65) | 10.92 (3.69) | 11.23 (3.55)^a^ | 10.83 (3.67) | | 11.31 (3.6)^a^ | 10.84 (3.72) | | 11.23 (3.56)^a^ |  |  |
| Short-term variability | 4.89 (1.65) | 4.82 (1.68) | 5.04 (1.59)^a^ | 4.78 (1.68) | | 5.05 (1.6)^a^ | 4.79 (1.71) | | 5.02 (1.58)^a^ |  |  |
| **Mean Blood Pressure (mm Hg)** |  |  |  |  | |  |  | |  |  |  |
| Maximum | 167.7 (27.5) | 166.9 (27.9) | 169.4 (26.4)^a^ | 166.5 (28.2) | | 169.6 (26.3)^a^ | 166.3 (28.5) | | 169.4 (26.2)^a^ |  |  |
| Minimum | 22.4 (20.1) | 24.0 (21.3) | 19.3 (17.0)^a^ | 24.5 (21.7) | | 19.1 (16.7)^a^ | 24.5 (22.0) | | 19.8 (17.3)^a^ |  |  |
| Average of base signal | 76.3 (12.4) | 78.1 (12.9) | 72.7 (10.4)^a^ | 78.3 (13.0) | | 72.5 (10.3)^a^ | 79.0 (13.2) | | 73.1 (10.4)^a^ |  |  |
| Long-term variability | 14.5 (5.3) | 14.3 (5.5) | 14.8 (5.1)^a^ | 14.2 (5.4) | | 14.9 (5.2)^a^ | 14.2 (5.5) | | 14.8 (5.1)^a^ |  |  |
| Short-term variability | 6.0 (2.1) | 5.9 (2.1) | 6.2 (1.9)^a^ | 5.9 (2.1) | | 6.2 (2.0)^a^ | 5.9 (2.1) | | 6.2 (1.9)^a^ |  |  |
| **Heart rate (beats/minute)** |  |  |  |  | |  |  | |  |  |  |
| Maximum | 149.2 (30.24) | 147.95 (29.95) | 151.57 (30.65)^a^ | 147.22 (29.73) | | 152.16 (30.76)^a^ | 146.62 (29.62) | | 152.22 (30.68)^a^ |  |  |
| Minimum | 38.1 (20.97) | 40.24 (20.83) | 34.02 (20.64)^a^ | 40.96 (20.87) | | 33.81 (20.4)^a^ | 41.1 (20.66) | | 34.59 (20.8)^a^ |  |  |
| Average of base signal | 83.9 (14.61) | 83.49 (14.6) | 84.69 (14.61)^a^ | 83.32 (14.52) | | 84.78 (14.71)^a^ | 82.95 (14.5) | | 85.02 (14.66)^a^ |  |  |
| Long-term variability | 14.84 (8.4) | 14.18 (8.13) | 16.1 (8.77)^a^ | 13.87 (7.88) | | 16.29 (8.94)^a^ | 13.73 (7.83) | | 16.14 (8.85)^a^ |  |  |
| Short-term variability | 5.29 (1.76) | 5.17 (1.78) | 5.53 (1.7)^a^ | 5.12 (1.79) | | 5.56 (1.69)^a^ | 5.08 (1.79) | | 5.55 (1.69)^a^ |  |  |
| **Minimum alveolar concentration** |  |  |  |  | |  |  | |  |  |  |
| Maximum | 2.63 (0.89) | 2.58 (0.9) | 2.72 (0.87)^a^ | 2.56 (0.91) | | 2.73 (0.87)^a^ | 2.55 (0.91) | | 2.71 (0.87)^a^ |  |  |
| Minimum | 0.02 (0.06) | 0.02 (0.06) | 0.02 (0.05) | 0.02 (0.06) | | 0.02 (0.05) | 0.02 (0.06) | | 0.02 (0.05) |  |  |
| Average of base signal | 0.58 (0.17) | 0.58 (0.17) | 0.58 (0.18) | 0.58 (0.17) | | 0.58 (0.18) | 0.58 (0.17) | | 0.58 (0.18) |  |  |
| Long-term variability | 0.35 (0.19) | 0.33 (0.18) | 0.37 (0.2)^a^ | 0.33 (0.18) | | 0.37 (0.2)^a^ | 0.33 (0.18) | | 0.37 (0.2)^a^ |  |  |
| Short-term variability | 0.09 (0.05) | 0.09 (0.05) | 0.09 (0.05) | 0.09 (0.05) | | 0.09 (0.05) | 0.09 (0.05) | | 0.09 (0.05) |  |  |
| **Intraoperative laboratory results, median (25th-75th)** |  |  |  |  | |  |  | |  |  |  |
| **Arterial Blood Gas Panel** |  |  |  |  | |  |  | |  |  |  |
| **pH** |  |  |  |  | |  |  | |  |  |  |
| Maximum | 7.38 (7.33, 7.43) | 7.38 (7.34, 7.43) | 7.38 (7.32, 7.43) | 7.38 (7.34, 7.43) | | 7.38 (7.32, 7.43) | 7.38 (7.34, 7.43) | | 7.38 (7.33, 7.43) |  |  |
| Mean | 7.36 (7.32, 7.4) | 7.36 (7.33, 7.41) | 7.36 (7.31, 7.4)^a^ | 7.36 (7.33, 7.41) | | 7.36 (7.32, 7.4)^a^ | 7.36 (7.33, 7.41) | | 7.36 (7.32, 7.4)^a^ |  |  |
| Minimum | 7.34 (7.3, 7.39) | 7.34 (7.31, 7.39) | 7.34 (7.29, 7.38)^a^ | 7.34 (7.31, 7.39) | | 7.34 (7.3, 7.38)^a^ | 7.35 (7.31, 7.39) | | 7.34 (7.29, 7.38)^a^ |  |  |
| **Partial pressure of carbon dioxide (mm Hg)** |  |  |  |  | |  |  | |  |  |  |
| Maximum | 42.8 (38.4, 47.2) | 42.8 (38.2, 47.0) | 43.4 (38.7, 47.6)^a^ | 42.8 (38.2, 47.0) | | 43.1 (38.8, 47.5) | 42.8 (38.2, 46.9) | | 43.1 (38.7, 47.5)^a^ |  |  |
| Mean | 40.8 (36.9, 45.2) | 40.8 (36.8, 45.2) | 40.9 (37.1, 45.2) | 40.8 (36.7, 45.0) | | 40.9 (37.2, 45.4)^a^ | 40.8 (36.7, 45.0) | | 40.8 (37.2, 45.4) |  |  |
| Minimum | 38.9 (34.6, 44.6) | 38.8 (34.5, 44.5) | 39.3 (34.7, 44.8) | 38.8 (34.4, 44.4) | | 39.5 (34.8, 44.8)^a^ | 38.8 (34.3, 44.4) | | 39.2 (34.7, 44.8) |  |  |
| Variance | 0 (0, 6.5) | 0 (0, 5.9) | 0 (0, 7.2) | 0 (0, 6.3) | | 0 (0, 6.5) | 0 (0, 5.9) | | 0 (0, 6.9) |  |  |
| **Bicarbonate in Arterial (mmol/L)** |  |  |  |  | |  |  | |  |  |  |
| Maximum | 23.3 (21.7,25.0) | 23.3 (21.7, 24.9) | 23.3 (21.6, 25.2) | 23.3 (21.7, 24.9) | | 23.3 (21.7, 25.2) | 23.3 (21.7, 24.9) | | 23.3 (21.6, 25.2) |  |  |
| Mean | 22.7 (21.2, 24.4) | 22.7 (21.3, 24.4) | 22.5 (21.0, 24.4) | 22.7 (21.2, 24.4) | | 22.7 (21.1, 24.4) | 22.7 (21.3, 24.4) | | 22.7 (21.0, 24.4) |  |  |
| Minimum | 22.0 (20.4, 24.1) | 22.7 (21.3, 24.4) | 22.5 (21.0, 24.4) | 22.1 (20.6, 24.1) | | 22.0 (20.3, 24.0) | 22.7 (21.3, 24.4) | | 22.7 (21.0, 24.4) |  |  |
| Variance | 0 (0, 0.8) | 0 (0, 0.7) | 0 (0, 1.3) | 0 (0, 0.7) | | 0 (0, 1.2) | 0 (0, 0.7) | | 0 (0, 1.2) |  |  |
| **O2 saturation (%)** |  |  |  |  | |  |  | |  |  |  |
| Maximum | 99.8 (99.0, 100) | 99.8 (99.0, 100) | 99.9 (99.0, 100) | 99.8 (99, 100) | | 99.9 (99, 100) | 99.8 (98.9, 100) | | 99.9 (99.0, 100)^a^ |  |  |
| Mean | 99.5 (98.4, 100) | 99.45 (98.4, 100) | 99.6 (98.4, 100) | 99.4 (98.4, 100) | | 99.6 (98.41, 100) | 99.4 (98.3, 100) | | 99.6 (98.5, 100)^a^ |  |  |
| Minimum | 99.3 (97.8, 100) | 99.3 (97.8, 100) | 99.4 (97.7, 100) | 99.2 (97.8, 100) | | 99.4 (97.8, 100) | 99.2 (97.7, 100) | | 99.4 (97.8, 100)^a^ |  |  |
| Variance | 0 (0, 0.02) | 0 (0, 0.02) | 0 (0, 0.01) | 0 (0, 0.02) | | 0 (0, 0.01) | 0 (0, 0.02) | | 0 (0, 0.01) |  |  |
| **O2 Content, Arterial (mL/dL)** |  |  |  |  | |  |  | |  |  |  |
| Maximum | 15.3 (14.0, 16.9) | 15.3 (14.0, 17.0) | 15.2 (13.9, 16.9) | 15.4 (14.1, 17.0) | | 15.2 (13.8, 16.9)^a^ | 15.4 (14.1, 17.0) | | 15.2 (13.9, 16.9)^a^ |  |  |
| Mean | 14.7 (13.4, 16.3) | 14.8 (13.5, 16.4) | 14.5 (13.2, 16.1)^a^ | 14.8 (13.5, 16.4) | | 14.5 (13.2, 16.1)^a^ | 14.8 (13.5, 16.5) | | 14.5 (13.2, 16.1)^a^ |  |  |
| Minimum | 14.2 (12.5, 16.0) | 14.3 (12.8, 16.1) | 13.9 (12.2, 15.8)^a^ | 14.3 (12.8, 16.1) | | 14 (12.3, 15.8)^a^ | 14.4 (12.9, 16.1) | | 14.0 (12.3, 15.8)^a^ |  |  |
| Variance | 0 (0, 0.9) | 0 (0, 0.8) | 0 (0, 1.2) | 0 (0, 0.8) | | 0 (0, 1.0) | 0 (0, 0.7) | | 0 (0, 1.1) |  |  |
| **Carboxyhemoglobin in arterial (%)** |  |  |  |  | |  |  | |  |  |  |
| Maximum | 2.2 (1.4, 3.0) | 2.1 (1.3, 2.9) | 2.3 (1.5, 3.2)^a^ | 2.1 (1.3, 2.9) | | 2.3 (1.5, 3.1)^a^ | 2.1 (1.3, 2.9) | | 2.3 (1.4, 3.1)^a^ |  |  |
| Mean | 2.0 (1.3, 2.8) | 1.9 (1.2, 2.7) | 2.1 (1.4, 2.9)^a^ | 1.9 (1.2, 2.7) | | 2.1 (1.4, 2.9)^a^ | 1.8 (1.2, 2.7) | | 2.1 (1.4, 2.8)^a^ |  |  |
| Minimum | 1.6 (1.1, 2.6) | 1.5 (1.0, 2.5) | 1.8 (1.2, 2.7)^a^ | 1.5 (1.0,2.5) | | 1.8 (1.2, 2.7)^a^ | 1.5 (1.0, 2.5) | | 1.8 (1.1, 2.6)^a^ |  |  |
| **Methemoglobin (%)** |  |  |  |  | |  |  | |  |  |  |
| Maximum | 0.8 (0.6, 1.0) | 0.8 (0.6, 0.9) | 0.8 (0.6, 1.0)^a^ | 0.8 (0.6, 0.9) | | 0.8 (0.6, 1.0)^a^ | 0.8 (0.6, 0.9) | | 0.8 (0.6, 1.0)^a^ |  |  |
| Mean | 0.7 (0.5, 0.9) | 0.7 (0.5, 0.8) | 0.7 (0.6, 0.9)^a^ | 0.7 (0.5, 0.8) | | 0.7 (0.6, 0.9)^a^ | 0.7 (0.5, 0.8) | | 0.7 (0.6, 0.9)^a^ |  |  |
| Minimum | 0.6 (0.4, 0.8) | 0.6 (0.4, 0.8) | 0.6 (0.4, 0.9)^a^ | 0.6 (0.4, 0.8) | | 0.6 (0.4, 0.9)^a^ | 0.6 (0.4, 0.8) | | 0.6 (0.4, 0.9)^a^ |  |  |
| Variance | 0 (0, 0.02) | 0 (0, 0.02) | 0 (0, 0.03) | 0 (0, 0.022) | | 0 (0, 0.024) | 0 (0, 0.02) | | 0 (0, 0.03) |  |  |
| **Complete Blood Count** |  |  |  |  | |  |  | |  |  |  |
| **White blood cells (thou/mm^3^)** |  |  |  |  | |  |  | |  |  |  |
| Maximum | 13.6 (9.9, 18.3) | 13.3 (9.9, 17.9) | 14.1 (9.7, 19.1)^a^ | 13.3 (9.9, 17.8) | | 14.0 (9.8, 19.3)^a^ | 13.2 (9.8, 17.7) | | 13.9 (9.9, 19.1)^a^ |  |  |
| Mean | 12.9 (9.3, 17.5) | 12.7 (9.3, 17.1) | 13.3 (9.1, 17.9) | 12.5 (9.3, 16.9) | | 13.3 (9.2, 18.3)^a^ | 12.4 (9.3, 16.8) | | 13.3 (9.3, 18.3)^a^ |  |  |
| Minimum | 12.1 (8.5, 16.9) | 11.8 (8.6, 16.6) | 12.6 (8.3, 17.5) | 11.7 (8.5, 16.3) | | 12.6 (8.4, 17.7)^a^ | 11.6 (8.5, 16.3) | | 12.7 (8.4, 17.6)^a^ |  |  |
| Variance | 0 (0, 0.85) | 0 (0, 0.86) | 0 (0, 0.85) | 0 (0, 1.07) | | 0 (0, 0.54)^a^ | 0 (0, 1.12) | | 0 (0, 0.54)^a^ |  |  |
| **Red blood cells (million/mcL)** |  |  |  |  | |  |  | |  |  |  |
| Maximum | 3.59 (3.23, 3.97) | 3.6 (3.25, 3.98) | 3.58 (3.22, 3.96) | 3.6 (3.25, 3.98) | | 3.57 (3.22, 3.94) | 3.6 (3.26, 3.99) | | 3.57 (3.21, 3.94)^a^ |  |  |
| Mean | 3.5 (3.16, 3.87) | 3.51 (3.18, 3.88) | 3.48 (3.11, 3.86)^a^ | 3.52 (3.19, 3.9) | | 3.46 (3.12, 3.85)^a^ | 3.53 (3.19, 3.9) | | 3.46 (3.12, 3.85)^a^ |  |  |
| Minimum | 3.42 (3.07, 3.83) | 3.44 (3.1, 3.84) | 3.38 (3.0, 3.81)^a^ | 3.45 (3.11, 3.85) | | 3.38 (3.0, 3.8)^a^ | 3.46 (3.12, 3.86) | | 3.38 (3.01, 3.8)^a^ |  |  |
| Variance | 0 (0, 0.02) | 0 (0, 0.01) | 0 (0, 0.02) | 0 (0, 0.01) | | 0 (0, 0.02) | 0 (0, 0.01) | | 0 (0, 0.02) |  |  |
| **Hemoglobin in arterial (g/dL)** |  |  |  |  | |  |  | |  |  |  |
| Maximum | 11.0 (10.0, 12.2) | 11.1 (10.1, 12.2) | 11.0 (9.9, 12.2) | 11.1 (10.1, 12.2) | | 11.0 (9.9, 12.2) | 11.1 (10.2, 12.3) | | 11.0 (9.9, 12.2)^a^ |  |  |
| Mean | 10.6 (9.6, 11.8) | 10.6 (9.7, 11.85) | 10.5 (9.5, 11.6)^a^ | 10.7 (9.7, 11.9) | | 10.5 (9.5, 11.6)^a^ | 10.7 (9.76, 11.9) | | 10.5 (9.5, 11.6)^a^ |  |  |
| Minimum | 10.2 (9.0, 11.6) | 10.3 (9.1, 11.6) | 10.1 (8.7, 11.5)^a^ | 10.3 (9.2, 11.7) | | 10.1 (8.8, 11.5)^a^ | 10.4 (9.2, 11.7) | | 10.1 (8.8, 11.4)^a^ |  |  |
| Variance | 0 (0, 0.4) | 0 (0, 0.4) | 0 (0, 0.6) | 0 (0, 0.4) | | 0 (0, 0.6) | 0 (0, 0.36) | | 0 (0, 0.6) |  |  |
| **Hemoglobin (g/dL)** |  |  |  |  | |  |  | |  |  |  |
| Maximum | 10.8 (9.8,12.0) | 10.8 (9.9,12.0) | 10.8 (9.8,11.98) | 10.8 (9.9,12.0) | | 10.8 (9.8,11.9) | 10.8 (9.9,12.1) | | 10.8 (9.8,11.9)^a^ |  |  |
| Mean | 10.3 (9.3,11.6) | 10.4 (9.3,11.6) | 10.2 (9.2,11.4)^a^ | 10.45 (9.3,11.7) | | 10.2 (9.2,11.4)^a^ | 10.5 (9.4,11.7) | | 10.2 (9.2,11.4)^a^ |  |  |
| Minimum | 10.6 (9.6,11.7) | 10.6 (9.6,11.8) | 10.5 (9.6,11.6)^a^ | 10.6 (9.65,11.8) | | 10.5 (9.6,11.56)^a^ | 10.7 (9.65,11.8) | | 10.5 (9.55,11.6)^a^ |  |  |
| Variance | 0.0 (0.0,0.13) | 0.0 (0.0,0.12) | 0.0 (0.0,0.18) | 0.0 (0.0,0.12) | | 0.0 (0.0,0.18) | 0.0 (0.0,0.12) | | 0.0 (0.0,0.17) |  |  |
| **Hematocrit whole blood (%)** |  |  |  |  | |  |  | |  |  |  |
| Maximum | 31.5 (28.7, 34.9) | 31.5 (28.8, 34.9) | 31.4 (28.5, 34.9) | 31.6 (28.8, 35.5) | | 31.4 (28.5, 34.8) | 31.7 (28.9, 35.1) | | 31.4 (28.5, 34.7)^a^ |  |  |
| Mean | 30.8 (28.0, 34.1) | 30.9 (28.2, 34.2) | 30.6 (27.7, 34.0)^a^ | 30.9 (28.2, 34.3) | | 30.6 (27.8, 33.8)^a^ | 30.9 (28.2, 34.3) | | 30.6 (27.8, 33.8)^a^ |  |  |
| Minimum | 30.2 (27.1, 33.6) | 30.4 (27.4, 33.7) | 29.8 (26.6, 33.4)^a^ | 30.5 (27.4, 33.9) | | 29.9 (26.7, 33.3)^a^ | 30.6 (27.5, 33.9) | | 29.9 (26.7, 33.3)^a^ |  |  |
| Variance | 0 (0, 1.1) | 0 (0, 1.0) | 0 (0, 1.5) | 0 (0, 1.1) | | 0 (0, 1.3) | 0 (0, 1.1) | | 0 (0, 1.2) |  |  |
| **Mean corpuscular volume (fL/red cell)** |  |  |  |  | |  |  | |  |  |  |
| Maximum | 88.6 (85.2, 92.2) | 88.4 (85.1, 92.3) | 88.7 (85.4, 92.0) | 88.4 (85.2, 92.0) | | 88.7 (85.3, 92.2) | 88.4 (85.2, 92.2) | | 88.7 (85.3, 92.2) |  |  |
| Mean | 88.2 (85.0, 91.8) | 88.2 (85.0, 92.0) | 88.3 (85.2, 91.4) | 88.2 (85.1, 91.9) | | 88.3 (85.0, 91.6) | 88.1 (85.1, 91.9) | | 88.3 (85.0, 91.7) |  |  |
| Minimum | 87.9 (84.7, 91.4) | 87.9 (84.7, 91.8) | 87.9 (84.8, 90.9) | 87.9 (84.8, 91.7) | | 87.9 (84.7, 91.0) | 87.9 (84.9, 91.6) | | 88.0 (84.7, 91.3) |  |  |
| **Mean corpuscular hemoglobin (pg)** |  |  |  |  | |  |  | |  |  |  |
| Maximum | 30.6 (29.4, 31.8) | 30.6 (29.4, 31.8) | 30.5 (29.4, 31.8) | 30.6 (29.4, 31.8) | | 30.5 (29.4, 31.8) | 30.6 (29.4, 31.8) | | 30.5 (29.4, 31.8) |  |  |
| Mean | 30.4 (29.3, 31. 7) | 30.5 (29.2, 31.7) | 30.4 (29.3, 31.6) | 30.5 (29.3, 31.7) | | 30.4 (29.3, 31.6) | 30.4 (29.3, 31.7) | | 30.4 (29.3, 31.6) |  |  |
| Minimum | 30.3 (29.1, 31.5) | 30.3 (29.1,31.6) | 30.2 (29.2, 31.5) | 30.3 (29.1, 31.52) | | 30.2 (29.1, 31.5) | 30.3 (29.1, 31.5) | | 30.3 (29.1, 31.5) |  |  |
| Variance | 0 (0, 0.04) | 0 (0, 0.04) | 0 (0, 0.05) | 0 (0, 0.05) | | 0 (0, 0.04) | 0 (0, 0.05) | | 0 (0, 0.04) |  |  |
| **Mean corpuscular hemoglobin concentration (g/dL)** |  |  |  |  | |  |  | |  |  |  |
| Maximum | 34.5 (33.5, 35.5) | 34.4 (33.5, 35.5) | 34.5 (33.6, 35.5) | 34.4 (33.5, 35.5) | | 34.5 (33.6, 35,5) | 34.4 (33.5, 35.5) | | 34.5 (33.5, 35.5) |  |  |
| Mean | 34.3 (33.4, 35.3) | 34.3 (33.35, 35.3) | 34.4 (33.5, 35.3) | 34.3 (33.3, 35.3) | | 34.3 (33.5, 35.3) | 34.3 (33.4, 35.3) | | 34.3 (33.5, 35.3) |  |  |
| Minimum | 34.1 (33.2, 35.1) | 34.1 (33.2, 35.1) | 34.2 (33.3, 35.1) | 34.1 (33.2, 35.1) | | 34.2 (33.3, 35.1) | 34.1 (33.2, 35.1) | | 34.2 (33.3, 35.1) |  |  |
| **Red cell distribution width (%)** |  |  |  |  | |  |  | |  |  |  |
| Maximum | 14.9 (13.9, 16.1) | 14.6 (13.7, 15.8) | 15.3 (14.4, 16.7)^a^ | 14.6 (13.7, 15.7) | | 15.3 (14.4, 16.7)^a^ | 14.5 (13.6, 15.6) | | 15.3 (14.4, 16.6)^a^ |  |  |
| Mean | 14.8 (13.8, 15.9) | 14.6 (13.7, 15.7) | 15.2 (14.4, 16.5)^a^ | 14.5 (13.6, 15.5) | | 15.2 (14.4, 16.5)^a^ | 14.4 (13.6, 15.5) | | 15.2 (14.3, 16.5)^a^ |  |  |
| Minimum | 14.7 (13.8,15.9) | 14.5 (13.6, 15.6) | 15.1 (14.2,16.3)^a^ | 14.4 (13.6, 15.5) | | 15.1 (14.2, 16.4)^a^ | 14.3 (13.6, 15.3) | | 15.1 (14.2, 16.3)^a^ |  |  |
| Variance | 0 (0, 0.01) | 0 (0, 0.01) | 0 (0, 0.02) | 0 (0, 0.01) | | 0 (0, 0.02) | 0 (0, 0.01) | | 0 (0, 0.01) |  |  |
| **Platelet count (thou/mm^3^)** |  |  |  |  | |  |  | |  |  |  |
| Maximum | 180 (133, 239) | 194 (143, 251) | 161 (116, 207)^a^ | 197 (145, 253) | | 163 (117, 209)^a^ | 198 (147, 254) | | 165 (118, 211)^a^ |  |  |
| Mean | 176 (128, 229) | 189 (139, 243) | 154 (109, 201)^a^ | 190 (140, 245) | | 156 (110, 203)^a^ | 192 (142, 246) | | 159 (112, 207)^a^ |  |  |
| Minimum | 171 (121, 223) | 183 (133, 238) | 149 (102, 194)^a^ | 184 (135, 240) | | 151 (104, 198)^a^ | 185 (137, 242) | | 155 (106, 202)^a^ |  |  |
| Variance | 0 (0, 78) | 0 (0, 74) | 0 (0, 83) | 0 (0, 85) | | 0 (0, 61)^a^ | 0 (0, 98) | | 0 (0, 61)^a^ |  |  |
| **Lactic acid (mmol/L)** |  |  |  |  | |  |  | |  |  |  |
| Maximum | 2.5 (1.5, 4.2) | 2.1 (1.4, 3.4) | 3.6 (2.1, 5.4)^a^ | 2.1 (1.3, 3.3) | | 3.4 (2.0, 5.2)^a^ | 2.0 (1.3, 3.2) | | 3.3 (1.9, 5.2)^a^ |  |  |
| Mean | 2.2 (1.4, 3.6) | 1.8 (1.2, 2.9) | 2.9 (1.9, 4.7)^a^ | 1.8 (1.2, 2.8) | | 2.8 (1.8, 4.6)^a^ | 1.7 (1.2, 2.8) | | 2.8 (1.8, 4.4)^a^ |  |  |
| Minimum | 1.6 (1.1, 2.9) | 1.4 (1.0, 2.5) | 2.2 (1.3, 4.2)^a^ | 1.4 (1.0, 2.4) | | 2.2 (1.3, 4.0)^a^ | 1.4 (1.0, 2.3) | | 2.1 (1.3, 3.7)^a^ |  |  |
| Variance | 0 (0, 0.3) | 0 (0, 0.2) | 0 (0, 0.5)^a^ | 0 (0, 0.2) | | 0 (0, 0.5) | 0 (0, 0.2) | | 0 (0, 0.5)^a^ |  |  |
| **Mean Platelet Volume (fL)** |  |  |  |  | |  |  | |  |  |  |
| Maximum | 8.0 (7.5, 8.7) | 7.9 (7.4, 8.5) | 8.2 (7.6, 8.9)^a^ | 7.9 (7.4, 8.5) | | 8.2 (7.6, 8.8)^a^ | 7.9 (7.4, 8.5) | | 8.2 (7.6, 8.8)^a^ |  |  |
| Mean | 7.9 (7.4, 8.5) | 7.8 (7.4, 8.4) | 8.1 (7.5, 8.7)^a^ | 7.8 (7.3, 8.4) | | 8.1 (7.5, 8.7)^a^ | 7.8 (7.3, 8.4) | | 8.0 (7.5, 8.7)^a^ |  |  |
| Minimum | 7.8 (7.3, 8.4) | 7.7 (7.2, 8.3) | 7.9 (7.3, 8.6)^a^ | 7.7 (7.2, 8.3) | | 7.9 (7.4, 8.6)^a^ | 7.7 (7.2, 8.2) | | 7.9 (7.4, 8.6)^a^ |  |  |
| Variance | 0 (0, 0.03) | 0 (0, 0.03) | 0 (0, 0.02) | 0 (0, 0.04) | | 0 (0, 0.02) | 0 (0, 0.04) | | 0 (0, 0.02) |  |  |
|  |  |  |  |  | |  |  | |  |  |  |
| **Ratio of partial pressure arterial oxygen and fraction of inspired oxygen (mmHg)** | 90 (75, 118) | 92 (75, 122) | 87.6 (74, 113)^a^ | 92 (76, 122) | | 88 (74, 114)^a^ | 91 (75, 122) | | 89 (74, 115)^a^ |  |  |
|  |  |  |  |  | |  |  | |  |  |  |
|  |  |  |  |  | |  |  | |  |  |  |
|  |  |  |  |  | |  |  | |  |  |  |
|  |  |  |  |  | |  |  | |  |  |  |
| **Intraoperative medications, n (%)** |  |  |  |  | |  |  | |  |  |  |
| Total administered intravenous fluids (ml) | 2300 (1400, 3600) | 2400  (1500, 3600) | 2200 (1250, 3500) | 2435 (1500, 3700) | | 2200 (1250, 3500)^a^ | 2400 (1500, 3550) | | 2200 (1250, 3600) |  |  |
| Total administered blood products (ml) | 318 (0, 1250) | 0 (0, 750) | 793 (0, 2750)^a^ | 0 (0, 750) | | 750 (0, 2500)^a^ | 0 (0, 727) | | 750 (0, 2486)^a^ |  |  |
| Diuretic (vs. No) | 329 (11) | 141 (7) | 188 (19)^a^ | 119 (7) | | 210 (18)^a^ | 100 (6) | | 229 (17)^a^ |  |  |
| Vasopressors (vs. No) | 1942 (67) | 1201 (63) | 741 (74)^a^ | 1084 (62) | | 858 (74)^a^ | 970 (62) | | 972 (73)^a^ |  |  |
| **Other variables** |  |  |  |  | |  |  | |  |  |  |
| General anesthesia, n (%) | 2881 (99) | 1893 (99) | 988 (99) | 1728 (100) | | 1153 (99) | 1553 (99) | | 1328 (99.18) |  |  |
| Duration of surgery (min), median (25th-75th) | 387 (294, 483) | 366 (275, 455) | 427 (338, 525)^a^ | 357 (271, 449) | | 425 (340, 521)^a^ | 352 (265, 443) | | 424 (338, 52)^a^ |  |  |
| Surgery performed between 7 pm and 7 am , n (%) | 394 (14) | 238 (12) | 156 (16)^a^ | 210 (12) | | 184 (16)^a^ | 167 (11) | | 227 (17)^a^ |  |  |
|  |  |  |  |  | |  |  | |  |  |  |
| Total estimated blood loss in ml | 150 (150, 500) | 150 (150, 500) | 150 (150, 388)^a^ | 150 (150, 500) | | 150 (150, 350)^a^` | 150 (150, 500) | | 150 (150, 400)^a^ |  |  |
| Total urine output (ml) | 650 (300, 1150) | 650 (300, 1100) | 650 (346, 1200) | 600 (300, 1100) | | 700 (350, 1200)^a^ | 600 (300, 1100) | | 700 (350, 1200)^a^ |  |  |
| **Outcomes** |  |  |  |  | |  |  | |  |  |  |
| Acute kidney injury |  |  |  |  | |  |  | |  |  |  |
| Onset within 3 days of surgery | 998 (34) | 0 (0) | 998 (100) | 0 (0) | | 998 (86) | 0 (0) | | 998 (75) |  |  |
| Onset within 7 days of surgery | 1163 (40) | 165 (9) | 998 (100) | 0 (0) | | 1163 (100) | 0 (0) | | 1163 (87) |  |  |
| At any time after surgery | 1339 (46) | 341 (18) | 998 (100) | 176 (10) | | 1163 (100) | 0 (0) | | 1339 (100) |  |  |
| Worst Stage of acute kidney injury^c^ |  |  |  |  | |  |  | |  |  |  |
| Stage 1 | 695 (52) |  | 627 (63) |  | | 685 (59) |  | | 695 (52) |  |  |
| Stage 2 | 303 (23) |  | 186 (19) |  | | 237 (20) |  | | 303 (23) |  |  |
| Stage 3 | 338 (25) |  | 182 (18) |  | | 238 (20) |  | | 338 (25) |  |  |
| Renal replacement therapy | 154 (12) |  | 137 (14) |  | | 142 (12) |  | | 154 (12) |  |  |
| Intensive care unit admission > 48 hours | 1800 (62) | 1018 (53) | 782 (78) | 894 (51) | | 906 (78) | 745 (47) | | 1055 (79) |  |  |
| Mechanical ventilation > 48 hours | 833 (29) | 380 (20) | 453 (45) | 322 (18) | | 511 (44) | 226 (14) | | 607 (45) |  |  |
| Wound complications | 73 (3) | 38 (2) | 35 (4) | 29 (2) | | 44 (4) | 13 (1) | | 60 (4) |  |  |
| Neurological complications or delirium | 246 (8) | 156 (8) | 90 (9) | 142 (8) | | 104 (9) | 112 (7) | | 134 (10) |  |  |
| Cardiovascular complications | 425 (15) | 209 (11) | 216 (22) | 168 (10) | | 257 (22) | 132 (8) | | 293 (22) |  |  |
| Sepsis | 266 (9) | 124 (6) | 142 (14) | 99 (6) | | 167 (14) | 57 (4) | | 209 (16) |  |  |
| Venous thromboembolism | 133 (5) | 69 (4) | 64 (6) | 61 (3) | | 72 (6) | 41 (3) | | 92 (7) |  |  |

^a^ p-value < 0.05 compared to the group who did not develop acute kidney injury.

^b^ Specialty surgery includes urological, orthopedics, gynecologic, ear nose throat surgeries, ophthalmology and plastic surgery.

^c^ Percentages are among patients with acute kidney injury. For overall cohort, numbers for acute kidney injury at any time during hospitalization are reported. Stage 3 includes cases with and without dialysis.
