# Supplementary material for: Improved predictive models for acute kidney injury with IDEA: Intraoperative Data Embedded Analytics
Source: PLoS One. 2019 Apr 4;14(4):e0214904. doi: 10.1371/journal.pone.0214904 (PMC6448850; doi:10.1371/journal.pone.0214904)
Supplement: S4 Table — (DOCX) [file pone.0214904.s005.docx]

**S4 Table. Model Performance for secondary outcomes.**

| **Outcome** | **Model** | **AUC**  **(95% CI)** | **Accuracy**  **(95% CI)** | **Sensitivity**  **(95% CI)** | **Specificity**  **(95% CI)** | **Positive Predicted Value**  **(95% CI)** | **Negative Predicted Value**  **(95% CI)** | **Net reclassification improvement (95% CI) for patients with AKI** | **Net reclassification improvement (95% CI) for patients without AKI** | **Overall Net reclassification improvement (95% CI)** |
| --- | --- | --- | --- | --- | --- | --- | --- | --- | --- | --- |
| **AKI onset within first three postoperative days** | Preop model | 0.83  (0.80, 0.86) | 0.70  (0.67, 0.73) | 0.82  (0.78, 0.87) | 0.64  (0.60, 0.68) | 0.55  (0.50, 0.60) | 0.87  (0.83,0.90) | - | - | - |
|  | Postop stacked model | 0.85  (0.82, 0.87) | 0.77  (0.74, 0.80) | 0.78  (0.73, 0.82) | 0.77  (0.73, 0.80) | 0.64  (0.60,0.69) | 0.86  (0.83, 0.89) | -4.90%  (-8.8%, -2%)  p-value = 0.004 | 13.05%  (10%, 16.2%)  p-value < 0.0001 | 8.15%  (4%, 13%)  p-value = 0.0006 |
|  | Postop full model | 0.85  (0.82, 0.87) | 0.79  (0.76, 0.82) | 0.77  (0.72, 0.81) | 0.80  (0.77, 0.84) | 0.68  (0.63, 0.73) | 0.87  (0.83, 0.89) | -5.56%  (-10.5%, -1%)  p-value = 0.02 | 16.58%  (12.9%, 20.1%)  p-value < 0.0001 | 11.02%  (5%, 17%)  p-value = 0.0004 |
| **AKI onset at any time after surgery** | Preop model | 0.84  (0.81, 0.86) | 0.76  (0.73, 0.79) | 0.70  (0.64, 0.74) | 0.83  (0.80, 0.86) | 0.79  (0.74, 0.83) | 0.75  (0.71, 0.79) | - | - | - |
|  | Postop stacked model | 0.85  (0.83, 0.88) | 0.78  (0.75, 0.80) | 0.80  (0.76, 0.83) | 0.76  (0.72, 0.80) | 0.76  (0.71, 0.79) | 0.80  (0.76, 0.84) | 9.55%  (6.7%, 12.4%)  p-value < 0.0001 | -5.73%  (-8.6%, -3.8%)  p-value < 0.0001 | 3.82%  (0.2%, 8%)  p-value = 0.04 |
|  | Postop full model | 0.86  (0.83, 0.88) | 0.79  (0.76, 0.82) | 0.75  (0.71, 0.79) | 0.83  (0.80, 0.87) | 0.81  (0.76, 0.84) | 0.78  (0.74, 0.82) | 5.25%  (1.7%, 9%)  p-value = 0.005 | 0.66%  (-3%, 3.96%)  p-value = 0.7 | 5.91%  (1%, 11%)  p-value = 0.02 |

Abbreviations. AUC, Area under curve, AKI, Acute kidney injury.
