# Supplementary material for: Improved predictive models for acute kidney injury with IDEA: Intraoperative Data Embedded Analytics
Source: PLoS One. 2019 Apr 4;14(4):e0214904. doi: 10.1371/journal.pone.0214904 (PMC6448850; doi:10.1371/journal.pone.0214904)
Supplement: S5 Table — (DOCX) [file pone.0214904.s006.docx]

**S5 Table. Summary of important features derived from postoperative models predicting acute kidney injury occurring within the seven days after surgery.**

| Postoperative stacked model | | | Postoperative full model | | |
| --- | --- | --- | --- | --- | --- |
| Rank | Variable | Feature importance score from Random Forest | Rank | Variable | Feature importance score from Random Forest |
| 1 | Risk probability for AKI from preoperative model | 0.40006 | 1 | Zip code | 0.21769 |
| 2 | Lactic acid - mean | 0.03299 | 2 | Chronic kidney disease | 0.05737 |
| 3 | Lactic acid - maximum | 0.03215 | 3 | Attending Surgeon | 0.04138 |
| 4 | Total blood products in ml | 0.02229 | 4 | Lactic acid - mean | 0.03581 |
| 5 | Red cell distribution width - mean | 0.01548 | 5 | Lactic acid - maximum | 0.03509 |
| 6 | Diastolic BP - time spent between 26 and 43 mmHg | 0.01499 | 6 | Total blood products in ml | 0.03003 |
| 7 | Diastolic BP - time spent between 43 and 51 mmHg | 0.01341 | 7 | Red cell distribution width - mean | 0.02013 |
| 8 | Red cell distribution width - maximum | 0.01258 | 8 | Red cell distribution width - maximum | 0.01690 |
| 9 | Platelet count - minimum | 0.01203 | 9 | Diastolic BP - time spent between 26 and 43 mmHg | 0.01404 |
| 10 | Diastolic BP - % time spent between 26 and 43 mmHg | 0.01198 | 10 | Red cell distribution width - minimum | 0.01398 |
| 11 | Platelet count - mean | 0.01086 | 11 | Age | 0.01336 |
| 12 | Red cell distribution width - minimum | 0.00976 | 12 | Diastolic BP - time spent between 43 and 51 mmHg | 0.01274 |
| 13 | Mean Arterial BP - time spent between 75 and 91 mmHg | 0.00936 | 13 | Diastolic BP - % time spent between 26 and 43 mmHg | 0.01116 |
| 14 | Platelet count - maximum | 0.00886 | 14 | Time of surgery from admission (days) | 0.01056 |
| 15 | Systolic BP - time spent between 75 and 91 mmHg | 0.00831 | 15 | Platelet count - mean | 0.01015 |
| 16 | Lactic acid - minimum | 0.00807 | 16 | Platelet count - minimum | 0.00994 |
| 17 | Systolic BP - time spent between 67 and 75 mmHg | 0.00796 | 17 | Mean Arterial BP - time spent between 75 and 91 mmHg | 0.00865 |
| 18 | Heart rate - time spent between 104 and 127 mmHg | 0.00785 | 18 | Lactic acid - minimum | 0.00824 |
| 19 | Total surgery time in mins | 0.00773 | 19 | Diastolic BP - base signal mean | 0.00775 |
| 20 | Heart rate - % time spent between 59 and 70 mmHg | 0.00762 | 20 | Diastolic BP - % time spent between 67 and 75 mmHg | 0.00767 |
| 21 | Heart rate - long-term variability | 0.00750 | 21 | Platelet count - maximum | 0.00733 |
| 22 | Heart rate - % time spent between 59 and 104 mmHg | 0.00724 | 22 | Bicarbonate in Arterial - min | 0.00723 |
| 23 | Diastolic BP - % time spent between 67 and 75 mmHg | 0.00692 | 23 | PaO2/FiO2 (PF) ratio | 0.00706 |
| 24 | Diastolic BP - % time spent between 75 and 91 mmHg | 0.00681 | 24 | Systolic BP - time spent between 75 and 91 mmHg | 0.00697 |
| 25 | Diastolic BP - base signal mean | 0.00677 | 25 | Charlson's comorbidity index | 0.00687 |
| 26 | Diastolic BP - % time spent between 43 and 51 mmHg | 0.00658 | 26 | Distance from Residency to Hospital (km) | 0.00655 |
| 27 | Diastolic BP - time spent between 67 and 75 mmHg | 0.00657 | 27 | Diastolic BP - % time spent between 43 and 51 mmHg | 0.00634 |
| 28 | Mean Platelet Volume - mean | 0.00651 | 28 | Systolic BP - time spent between 67 and 75 mmHg | 0.00602 |
| 29 | Diastolic BP - time spent between 75 and 91 mmHg | 0.00631 | 29 | Mean Arterial BP - time spent between 67 and 75 mmHg | 0.00519 |
| 30 | Heart rate - time spent between 93 and 104 bpm | 0.00606 | 30 | Diastolic BP - % time spent between 75 and 91 mmHg | 0.00512 |
| 31 | Diastolic BP - short-term variability | 0.00601 | 31 | Partial pressure of carbon dioxide - mean | 0.00503 |
| 32 | MAC - time spent <= 1.478 | 0.00586 | 32 | Total surgery time in mins | 0.00501 |
| 33 | Carboxyhemoglobin in arterial - maximum | 0.00580 | 33 | Diastolic BP - time spent between 67 and 75 mmHg | 0.00480 |
| 34 | Heart rate - short-term variability | 0.00574 | 34 | Partial pressure of carbon dioxide - maximum | 0.00476 |
| 35 | Carboxyhemoglobin in arterial - mean | 0.00570 | 35 | Systolic BP - time spent between 43 and 75 mmHg | 0.00470 |
| 36 | Mean Arterial BP - time spent between 67 and 75 mmHg | 0.00561 | 36 | Systolic BP - % time spent between 51 and 67 mmHg | 0.00468 |
| 37 | Heart rate - time spent > 127 | 0.00557 | 37 | Heart rate - maximum during the surgery | 0.00463 |
| 38 | MAC - % time spent <= 1.478 | 0.00555 | 38 | Heart rate - % time spent between 59 and 70 bpm | 0.00462 |
| 39 | Mean Arterial BP - time spent > 116 | 0.00551 | 39 | Mean Arterial BP - % time spent between 51 and 67 mmHg | 0.00452 |
| 40 | Mean Platelet Volume - maximum | 0.00547 | 40 | Heart rate - long-term variability | 0.00447 |
| 41 | Diastolic BP - long-term variability | 0.00544 | 41 | Mean Platelet Volume - mean | 0.00442 |
| 42 | Mean Arterial BP - % time spent > 116 | 0.00543 | 42 | Mean Arterial BP - time spent between 43 and 75 mmHg | 0.00440 |
| 43 | Carboxyhemoglobin in arterial - minimum | 0.00535 | 43 | Systolic BP - % time spent between 43 and 75 mmHg | 0.00437 |
| 44 | MAC - duration in mins | 0.00530 | 44 | MAC - time spent <= 1.478 | 0.00435 |
| 45 | Heart rate - duration in bpm | 0.00500 | 45 | Total urine output in ml | 0.00431 |
| 46 | Mean Platelet Volume - minimum | 0.00496 | 46 | Mean Arterial BP - % time spent between 43 and 75 mmHg | 0.00430 |
| 47 | Diastolic BP - time spent between 51 and 67 mmHg | 0.00492 | 47 | Diastolic BP - time spent between 75 and 91 mmHg | 0.00422 |
| 48 | Systolic BP - short-term variability | 0.00490 | 48 | Primary insurance group | 0.00417 |
| 49 | Bicarbonate in Arterial - variance | 0.00488 | 49 | pH - mean | 0.00414 |
| 50 | Mean Arterial BP - short-term variability | 0.00483 | 50 | White blood cells - min | 0.00409 |
| 51 | Mean Arterial BP - long-term variability | 0.00483 | 51 | Heart rate - % time spent between 104 and 127 bpm | 0.00409 |
| 52 | Diastolic BP - % time spent > 91 mmHg | 0.00483 | 52 | Heart rate - time spent between 104 and 127 bpm | 0.00401 |
| 53 | Systolic BP - long-term variability | 0.00473 | 53 | Mean Arterial BP - long-term variability | 0.00383 |
| 54 | Heart rate - minimum during the surgery | 0.00471 | 54 | MAC - % time spent <= 1.478 | 0.00381 |
| 55 | Diastolic BP - time spent between 43 and 75 mmHg | 0.00469 | 55 | Heart rate - % time spent between 70 and 93 bpm | 0.00371 |
| 56 | Methemoglobin - mean | 0.00460 | 56 | Heart rate - % time spent between 59 and 104 bpm | 0.00369 |
| 57 | Systolic BP - % time spent between 75 and 91 mmHg | 0.00447 | 57 | Mean Platelet Volume - maximum | 0.00369 |
| 58 | Mean Arterial BP - % time spent between 75 and 91 mmHg | 0.00434 | 58 | Red blood cells - minimum | 0.00368 |
| 59 | Mean Arterial BP - time spent between 43 and 75 mmHg | 0.00429 | 59 | Diastolic BP - % time spent between 51 and 67 mmHg | 0.00365 |
| 60 | Mean Arterial BP - minimum during the surgery | 0.00425 | 60 | Carboxyhemoglobin in arterial - minimum | 0.00364 |
| 61 | MAC - time spent between 0 and 1 | 0.00421 | 61 | Mean Arterial BP - time spent > 116 | 0.00360 |
| 62 | Systolic BP - time spent between 43 and 75 mmHg | 0.00412 | 62 | Mean Platelet Volume - minimum | 0.00359 |
| 63 | MAC - time spent between 0 and 1.1 | 0.00403 | 63 | Carboxyhemoglobin in arterial - maximum | 0.00354 |
| 64 | Systolic BP - minimum during the surgery | 0.00401 | 64 | Systolic BP - long-term variability | 0.00353 |
| 65 | Systolic BP - base signal mean | 0.00400 | 65 | Diastolic BP - time spent between 43 and 75 mmHg | 0.00352 |
| 66 | Mean Arterial BP - base signal mean | 0.00396 | 66 | Carboxyhemoglobin in arterial - mean | 0.00351 |
| 67 | Mean Arterial BP - % time spent between 51 and 67 mmHg | 0.00391 | 67 | Mean Arterial BP - % time spent between 75 and 91 mmHg | 0.00350 |
| 68 | Systolic BP - % time spent between 51 and 67 mmHg | 0.00374 | 68 | Heart rate - base signal mean | 0.00344 |
| 69 | Mean Arterial BP - % time spent between 43 and 75 mmHg | 0.00373 | 69 | White blood cells - maximum | 0.00344 |
| 70 | Methemoglobin - max | 0.00363 | 70 | Heart rate - duration in bpm | 0.00343 |
| 71 | Systolic BP - % time spent between 43 and 75 mmHg | 0.00360 | 71 | Mean Arterial BP - % time spent > 116 | 0.00341 |
| 72 | Mean Arterial BP - % time spent between 67 and 75 mmHg | 0.00346 | 72 | White blood cells - mean | 0.00335 |
| 73 | Systolic BP - % time spent between 67 and 75 mmHg | 0.00330 | 73 | Heart rate - minimum during the surgery | 0.00326 |
| 74 | Lactic acid -variance | 0.00307 | 74 | Systolic BP - % time spent between 75 and 91 mmHg | 0.00325 |
| 75 | Methemoglobin - minimum | 0.00306 | 75 | pH - minimum | 0.00324 |
| 76 | Lactic acid - abnormal percentage | 0.00303 | 76 | MAC - duration in mins | 0.00319 |
| 77 | Diastolic BP - duration in mmHg | 0.00296 | 77 | Diastolic BP - short-term variability | 0.00319 |
| 78 | Mean Arterial BP - duration in mmHg | 0.00295 | 78 | Mean Arterial BP - % time spent between 67 and 75 mmHg | 0.00317 |
| 79 | Systolic BP - Duration in mmHg | 0.00289 | 79 | Heart rate - short-term variability | 0.00316 |
| 80 | MAC - maximum during the surgery | 0.00286 | 80 | Heart rate - time spent between 36 and 59 bpm | 0.00314 |
| 81 | Diastolic BP - % time spent <= 26 mmHg | 0.00274 | 81 | O2 Content, Arterial - mean | 0.00313 |
| 82 | Mean corpuscular volume - variance | 0.00272 | 82 | Systolic BP - % time spent between 67 and 75 mmHg | 0.00310 |
| 83 | Hematocrit - variance | 0.00267 | 83 | Heart rate - % time spent > 127 | 0.00308 |
| 84 | Red blood cells - variance | 0.00262 | 84 | Mean Arterial BP - base signal mean | 0.00304 |
| 85 | Systolic BP - % time spent between 43 and 51 mmHg | 0.00252 | 85 | Mean Arterial BP - short-term variability | 0.00295 |
| 86 | Heart rate - % time spent <= 36 | 0.00248 | 86 | Diastolic BP - long-term variability | 0.00293 |
| 87 | Diastolic BP - minimum during the surgery | 0.00247 | 87 | Systolic BP - % time spent > 163 mmHg | 0.00292 |
| 88 | Mean Arterial BP - % time spent between 43 and 51 mmHg | 0.00239 | 88 | Systolic BP - base signal mean | 0.00288 |
| 89 | Mean Arterial BP - time spent between 51 and 67 mmHg | 0.00232 | 89 | Month of admission | 0.00287 |
| 90 | Systolic BP - time spent between 51 and 67 mmHg | 0.00214 | 90 | MAC - % time spent between 0 and 1 | 0.00285 |
| 91 | Heart rate - time spent <= 36 | 0.00208 | 91 | Diastolic BP - time spent between 51 and 67 mmHg | 0.00285 |
| 92 | Diastolic BP - time spent <= 26 mmHg | 0.00206 | 92 | Heart rate - % time spent between 93 and 104 bpm | 0.00285 |
| 93 | Systolic BP - % time spent <= 45 mmHg | 0.00204 | 93 | Systolic BP - short-term variability | 0.00277 |
| 94 | Mean corpuscular hemoglobin concentration - abnormal percentage | 0.00198 | 94 | Hematocrit -minimum | 0.00275 |
| 95 | Hemoglobin - variance | 0.00196 | 95 | O2 Content, Arterial - minimum | 0.00275 |
| 96 | Systolic BP - % time spent between 26 and 43 mmHg | 0.00183 | 96 | Methemoglobin - mean | 0.00272 |
| 97 | Mean Arterial BP - % time spent between 26 and 43 mmHg | 0.00181 | 97 | Heart rate - time spent between 93 and 104 bpm | 0.00271 |
| 98 | Mean Arterial BP - time spent between 43 and 51 mmHg | 0.00179 | 98 | Bicarbonate in Arterial - variance | 0.00266 |
| 99 | Systolic BP - time spent between 43 and 51 mmHg | 0.00178 | 99 | MAC - % time spent between 0 and 1.1 | 0.00263 |
| 100 | Red cell distribution width - variance | 0.00164 | 100 | Lactic acid - abnormal percentage | 0.00259 |
| 101 | Systolic BP - time spent <= 45 mmHg | 0.00163 | 101 | Systolic BP - time spent between 51 and 67 mmHg | 0.00254 |
| 102 | MAC - long-term variability | 0.00161 | 102 | Diastolic BP - duration in mmHg | 0.00254 |
| 103 | Mean Arterial BP - time spent between 26 and 43 mmHg | 0.00114 | 103 | Systolic BP - minimum during the surgery | 0.00252 |
| 104 | Diuretic | 0.00111 | 104 | Systolic BP - Duration in mmHg | 0.00250 |
| 105 | Systolic BP - time spent between 26 and 43 mmHg | 0.00105 | 105 | Mean Arterial BP - minimum during the surgery | 0.00250 |
| 106 | Mean Platelet Volume - abnormal percentage | 0.00099 | 106 | Hemoglobin in arterial - mean | 0.00244 |
| 107 | Mean corpuscular volume - abnormal percentage | 0.00085 | 107 | Admission Source | 0.00242 |
| 108 | Mean Arterial BP - time spent <= 33 | 0.00065 | 108 | Mean Arterial BP - time spent between 51 and 67 mmHg | 0.00239 |
| 109 | Red cell distribution width - abnormal percentage | 0.00063 | 109 | MAC - time spent between 0 and 1.1 | 0.00235 |
| 110 | Pressors | 0.00061 | 110 | Diastolic BP - % time spent > 91 mmHg | 0.00235 |
|  |  |  | 111 | Hemoglobin in arterial - minimum | 0.00230 |
|  |  |  | 112 | MAC - time spent between 0 and 1 | 0.00229 |
|  |  |  | 113 | Diastolic BP - time spent > 91 mmHg | 0.00224 |
|  |  |  | 114 | Hemoglobin - minimum | 0.00220 |
|  |  |  | 115 | Lactic acid -variance | 0.00218 |
|  |  |  | 116 | Mean Arterial BP - duration in mmHg | 0.00216 |
|  |  |  | 117 | Heart rate - time spent > 127 | 0.00208 |
|  |  |  | 118 | Heart rate - % time spent <= 36 | 0.00198 |
|  |  |  | 119 | Diuretic | 0.00194 |
|  |  |  | 120 | Mean Arterial BP - % time spent between 43 and 51 mmHg | 0.00190 |
|  |  |  | 121 | Hematocrit whole blood - mean | 0.00181 |
|  |  |  | 122 | Mean corpuscular volume - variance | 0.00174 |
|  |  |  | 123 | Hematocrit - variance | 0.00172 |
|  |  |  | 124 | Methemoglobin - minimum | 0.00171 |
|  |  |  | 125 | Diastolic BP - % time spent <= 26 mmHg | 0.00170 |
|  |  |  | 126 | Hemoglobin - variance | 0.00165 |
|  |  |  | 127 | Red blood cells - variance | 0.00162 |
|  |  |  | 128 | Heart rate - time spent <= 36 | 0.00161 |
|  |  |  | 129 | Systolic BP - % time spent between 43 and 51 mmHg | 0.00160 |
|  |  |  | 130 | Red cell distribution width - variance | 0.00159 |
|  |  |  | 131 | Methemoglobin - max | 0.00156 |
|  |  |  | 132 | Hematocrit whole blood - minimum | 0.00154 |
|  |  |  | 133 | Diastolic BP - minimum during the surgery | 0.00154 |
|  |  |  | 134 | Mean Arterial BP - time spent between 43 and 51 mmHg | 0.00153 |
|  |  |  | 135 | County | 0.00153 |
|  |  |  | 136 | Systolic BP - time spent between 43 and 51 mmHg | 0.00152 |
|  |  |  | 137 | MAC - maximum during the surgery | 0.00140 |
|  |  |  | 138 | Systolic BP - % time spent <= 45 mmHg | 0.00138 |
|  |  |  | 139 | Mean corpuscular hemoglobin concentration - abnormal percentage | 0.00136 |
|  |  |  | 140 | Diastolic BP - time spent <= 26 mmHg | 0.00132 |
|  |  |  | 141 | Diastolic BP - maximum during the surgery | 0.00130 |
|  |  |  | 142 | Systolic BP - % time spent between 26 and 43 mmHg | 0.00126 |
|  |  |  | 143 | Mean Arterial BP - % time spent between 26 and 43 mmHg | 0.00125 |
|  |  |  | 144 | Systolic BP - time spent <= 45 mmHg | 0.00119 |
|  |  |  | 145 | MAC - long-term variability | 0.00112 |
|  |  |  | 146 | Admitting Service | 0.00108 |
|  |  |  | 147 | Mean Platelet Volume - abnormal percentage | 0.00108 |
|  |  |  | 148 | Mean corpuscular hemoglobin - variance | 0.00093 |
|  |  |  | 149 | Admitting type (Medicine/Surgery) | 0.00087 |
|  |  |  | 150 | Admission Type (Emergent/Elective) | 0.00085 |
|  |  |  | 151 | Carboxyhemoglobin in arterial - variance | 0.00080 |
|  |  |  | 152 | Systolic BP - time spent between 26 and 43 mmHg | 0.00078 |
|  |  |  | 153 | Mean Arterial BP - time spent between 26 and 43 mmHg | 0.00076 |
|  |  |  | 154 | Mean corpuscular volume - abnormal percentage | 0.00069 |
|  |  |  | 155 | Red cell distribution width - abnormal percentage | 0.00065 |
|  |  |  | 156 | Carboxyhemoglobin in arterial - abnormal percentage | 0.00064 |
|  |  |  | 157 | Antiemetic on admission day | 0.00064 |
|  |  |  | 158 | Mean Arterial BP - time spent <= 33 | 0.00061 |
|  |  |  | 159 | Diuretics on admission day | 0.00048 |
|  |  |  | 160 | Bicarbonate on admission day | 0.00047 |
|  |  |  | 161 | White blood cells - abnormal percentage | 0.00044 |
|  |  |  | 162 | Gender | 0.00043 |
|  |  |  | 163 | Pressors | 0.00041 |
|  |  |  | 164 | Methemoglobin - count | 0.00040 |
|  |  |  | 165 | Hemoglobin in arterial - count | 0.00039 |
|  |  |  | 166 | Bicarbonate in Arterial - count | 0.00038 |
|  |  |  | 167 | O2 Content, Arterial - count | 0.00037 |
|  |  |  | 168 | Weekend admission | 0.00035 |
|  |  |  | 169 | pH - count | 0.00034 |
|  |  |  | 170 | Carboxyhemoglobin in arterial - count | 0.00033 |
|  |  |  | 171 | O2 saturation - count | 0.00033 |
|  |  |  | 172 | Statin on admission day | 0.00029 |
|  |  |  | 173 | Hemoglobin - abnormal percentage | 0.00027 |
|  |  |  | 174 | Is night surgery? | 0.00026 |
|  |  |  | 175 | Partial pressure of carbon dioxide - count | 0.00025 |
|  |  |  | 176 | Red blood cells - abnormal percentage | 0.00021 |
|  |  |  | 177 | Pressors or inotropes on admission day | 0.00014 |
|  |  |  | 178 | MAC - short-term variability | 0.00008 |
|  |  |  | 179 | Angiotensin-Converting-Enzyme Inhibitors on admission day | 0.00004 |
|  |  |  | 180 | Methemoglobin - abnormal percentage | 0.00002 |

Abbreviations. BP, blood pressure; MAC, minimum alveolar concentration; AKI , acute kidney injury; O2, oxygen; PaO2, Partial pressure of oxygen; FIO2, Fraction of inspired oxygen.
