# Supplementary figures and images for: Improved predictive models for acute kidney injury with IDEA: Intraoperative Data Embedded Analytics
Source: PLoS One. 2019 Apr 4;14(4):e0214904. doi: 10.1371/journal.pone.0214904 (PMC6448850; doi:10.1371/journal.pone.0214904)

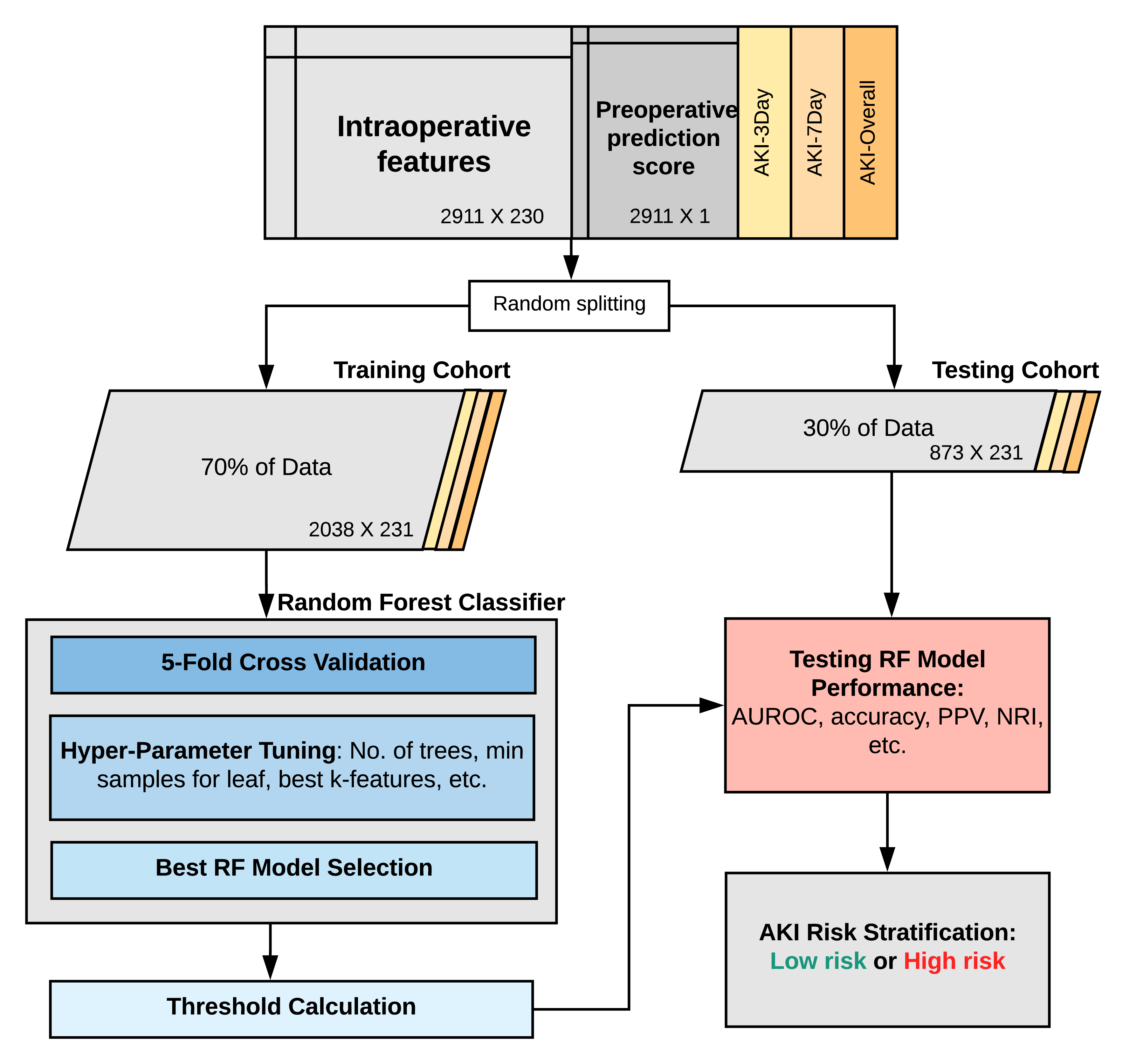

Supplement: S1 Fig — The cohort of size 2,911 with 231 features was randomly split into training (70%) and testing (30%) cohorts. A random forest classifier was used to train the AKI prediction model (we used 5-fold cross validation for hyperparameter tuning and feature selection) for all three outcomes separately and performance was tested using the testing (validation) cohort. (TIF) [file pone.0214904.s007.tif]

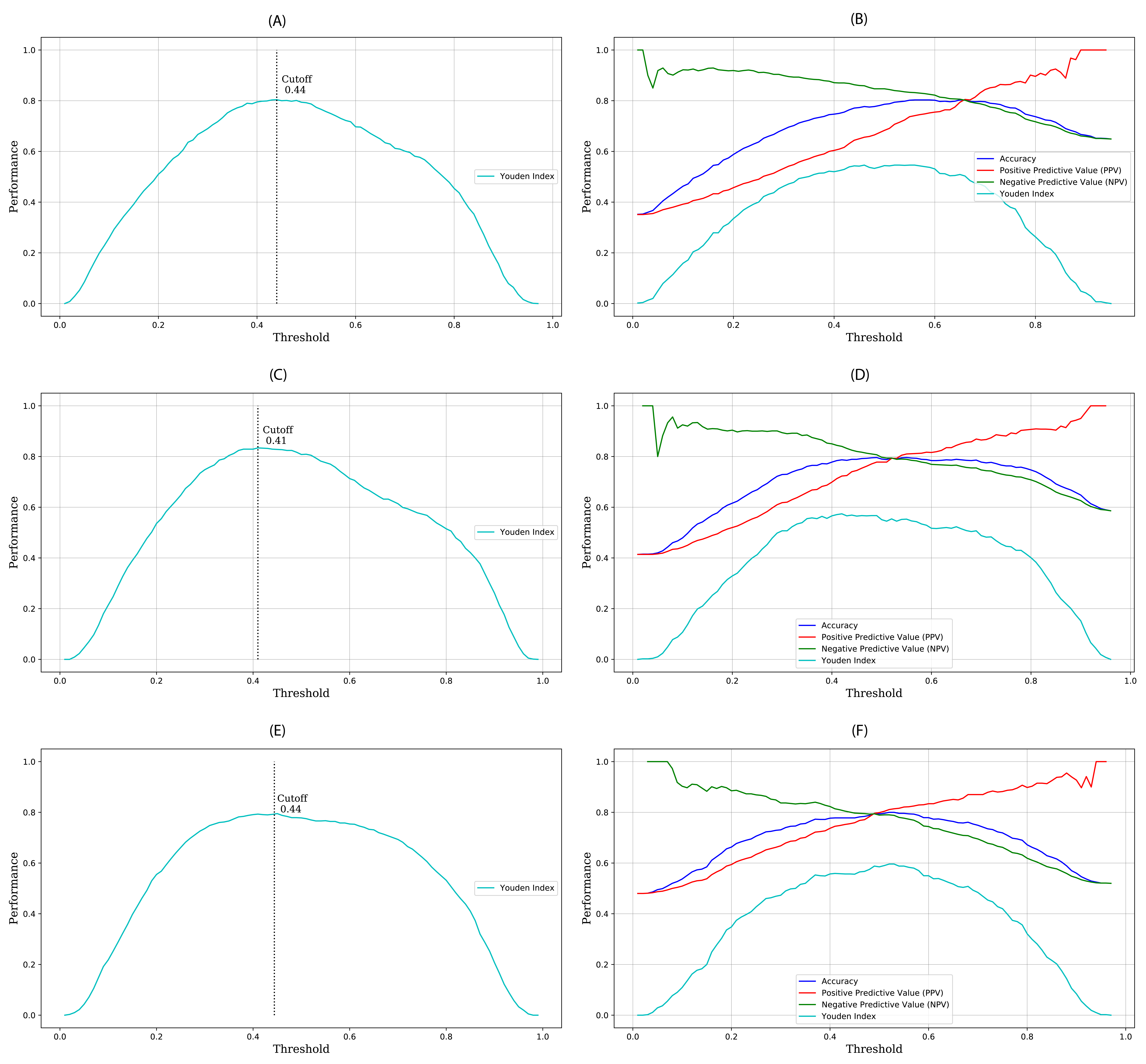

Supplement: S2 Fig — The first column shows the optimization of the cutoff threshold by maximizing the Youden Index. The second column shows the relationship between the performance metrics (accuracy, positive predictive value, negative predictive value, and Youden Index) and threshold. (A & B) The first row is for the prediction of postoperative acute kidney Injury (AKI) within three days of surgery outcome. (C & D) The second row is for the prediction of postoperative AKI within seven days of surgery outcome. (E & F) The third row is for the prediction of postoperative AKI prior to discharge outcome. (TIF) [file pone.0214904.s008.tif]

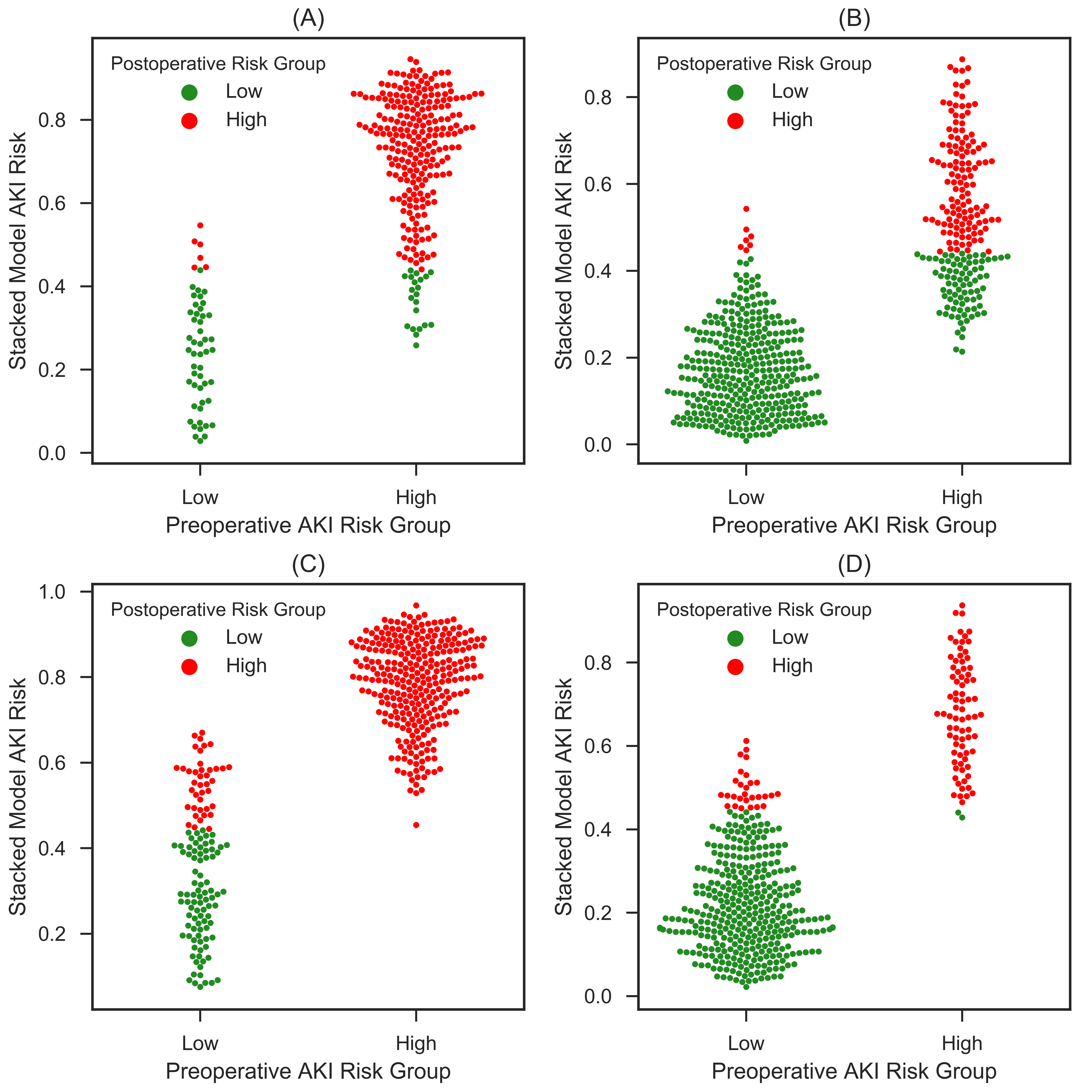

Supplement: S3 Fig — The first row represents the reclassification of (A) patients that developed postoperative acute kidney injury (AKI) within three days of surgery and (B) patients that did not develop postoperative AKI within three days of surgery for the three day secondary outcome. The second row represents the reclassification of (C) patients that developed postoperative AKI before discharge and (D) patients that did not develop postoperative AKI before discharge for the overall secondary outcome. (TIF) [file pone.0214904.s009.tif]
